# Mapping protein binding sites by photoreactive fragment pharmacophores

**DOI:** 10.1038/s42004-024-01252-w

**Published:** 2024-07-31

**Authors:** Péter Ábrányi-Balogh, Dávid Bajusz, Zoltán Orgován, Aaron B. Keeley, László Petri, Nikolett Péczka, Tibor Viktor Szalai, Gyula Pálfy, Márton Gadanecz, Emma K. Grant, Tímea Imre, Tamás Takács, Ivan Ranđelović, Marcell Baranyi, András Marton, Gitta Schlosser, Qirat F. Ashraf, Elvin D. de Araujo, Tamás Karancsi, László Buday, József Tóvári, András Perczel, Jacob T. Bush, György M. Keserű

**Affiliations:** 1 Medicinal Chemistry Research Group, HUN-REN Research Centre for Natural Sciences, Budapest, Hungary; 2 National Drug Research and Development Laboratory, HUN-REN Research Centre for Natural Sciences, Budapest, Hungary; 3 Department of Organic Chemistry and Technology, Faculty of Chemical Technology and Biotechnology, Budapest University of Technology and Economics, Budapest, Hungary; 4 Department of Inorganic and Analytical Chemistry, Faculty of Chemical Technology and Biotechnology, Budapest University of Technology and Economics, Budapest, Hungary; 5 Laboratory of Structural Chemistry and Biology & HUN-REN–ELTE Protein Modelling Research Group, Eötvös Loránd University, Budapest, Hungary; 6 Hevesy György PhD School of Chemistry, Eötvös Loránd University, Budapest, Hungary; 7 GlaxoSmithKline, Hertfordshire, UK; 8 MS Metabolomics Research Group, HUN-REN Research Centre for Natural Sciences, Budapest, Hungary; 9 Signal Transduction and Functional Genomics Research Group, HUN-REN Research Centre for Natural Sciences, Budapest, Hungary; 10 Doctoral School of Biology, Institute of Biology, ELTE Eötvös Loránd University, Budapest, Hungary; 11 National Tumor Biology Laboratory and Department of Experimental Pharmacology, National Institute of Oncology, Budapest, Hungary; 12 KINETO Lab Ltd, Budapest, Hungary; 13 Department of Pathology, Forensic and Insurance Medicine, Semmelweis University, Budapest, Hungary; 14 Department of Chemical and Environmental Process Engineering, Faculty of Chemical Technology and Biotechnology, Budapest University of Technology and Economics, Budapest, Hungary; 15 Waters Research Center, Budapest, Hungary; 16 MTA-ELTE Lendület Ion Mobility Mass Spectrometry Research Group, Eötvös Loránd University, Budapest, Hungary; 17 Department of Chemical & Physical Sciences, University of Toronto Mississauga, Mississauga, ON Canada; 18 Centre for Medicinal Chemistry, University of Toronto at Mississauga, Mississauga, ON Canada

**Keywords:** Chemical libraries, Drug discovery and development, Screening, Chemical tools

## Abstract

Fragment screening is a popular strategy of generating viable chemical starting points especially for challenging targets. Although fragments provide a better coverage of chemical space and they have typically higher chance of binding, their weak affinity necessitates highly sensitive biophysical assays. Here, we introduce a screening concept that combines evolutionary optimized fragment pharmacophores with the use of a photoaffinity handle that enables high hit rates by LC-MS-based detection. The sensitivity of our screening protocol was further improved by a target-conjugated photocatalyst. We have designed, synthesized, and screened 100 diazirine-tagged fragments against three benchmark and three therapeutically relevant protein targets of different tractability. Our therapeutic targets included a conventional enzyme, the first bromodomain of BRD4, a protein-protein interaction represented by the oncogenic KRas^G12D^ protein, and the yet unliganded *N*-terminal domain of the STAT5B transcription factor. We have discovered several fragment hits against all three targets and identified their binding sites via enzymatic digestion, structural studies and modeling. Our results revealed that this protocol outperforms screening traditional fully functionalized and photoaffinity fragments in better exploration of the available binding sites and higher hit rates observed for even difficult targets.

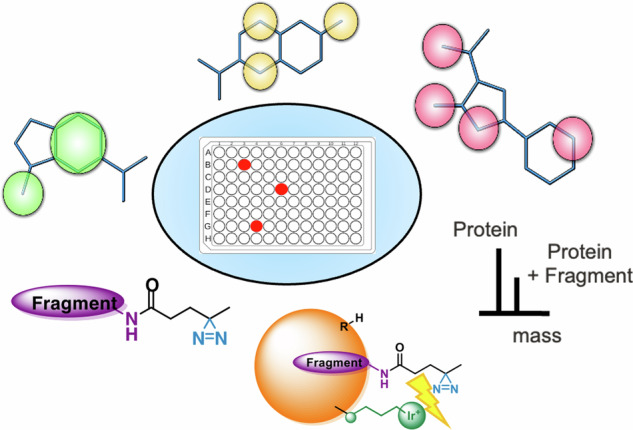

## Abstract (web summary)

Fragment screening is a popular strategy for generating viable chemical starting points for drug targets, however, weak affinity to targets, as well as the exploration of the binding site, remain challenging. Here, the authors develop pharmacophore-optimized photoaffinity fragments that can effectively explore the available binding site and enable a high hit rate and better sensitivity.

## Introduction

Fragment-based drug discovery (FBDD) has been a definitive trend of the past thirty years of drug discovery^1^, and has come of age with the approval of vemurafenib^2^ and further drugs that originated from FBDD programs^3–5^. The main rationale in generating chemical starting points by screening moderate-sized libraries of less complex, small and polar compounds comes from the realization that screening a few thousand fragments (≤16 heavy atoms) provides a better sampling of the respective chemical space^6^ than screening millions of larger compounds (≤36 heavy atoms), translating to substantially higher hit rates, typically at a smaller cost^1^. FBDD comes with its own challenges though, particularly in the experimental detection of the targeted binding site(s) of the respective hit(s). Biochemical screens are usually less sensitive to weak fragment binders and do not provide structural information on their binding site. Biophysical approaches such as the thermal shift assay, ligand-observed nuclear magnetic resonance (NMR) spectroscopy, surface plasmon resonance are typically used in primary fragment screening. Other techniques might offer more detailed results, but are more resource-intensive, e.g., X-ray screening needs the crystals of the target protein available for high-concentration soaking experiments mostly at synchrotrons or protein observed NMR that requires labeled protein samples and access to high-field NMR facilities.

For the simple and efficient detection of fragment hits, the concept of photoaffinity labeling has gained significant ground in the past years. Photoaffinity probes usually contain a pharmacophore pattern recognized by the target protein that forms non-covalent interactions, and a photoreactive group for the light-induced anchorage of the probe after the binding event^7,8^. The captured compound can be identified directly on the labeled protein by mass spectrometry (MS)^9^, or the probe might be equipped with a biorthogonal handle to enable downstream workflows^10,11^. This latter concept was pioneered at the Cravatt group introducing fully-functionalized fragments (FFFs)^12,13^ that were successfully utilized in diverse screening campaigns^14^, as well as chemical proteomics^15,16^. Notably, the concept has since been endorsed by several major chemical vendors that distribute commercial libraries of FFFs. Recently, a GSK team developed a screening platform (PhotoAffinity Bits, or PhABits), that captures fragment–protein interactions by photoaffinity labeling^17,18^. In our setup, members of the fragment library constitute the variable, pharmacophore-optimized non-covalent cores for target binding, and are equipped with a diazirine photoaffinity tag to enable anchorage and MS-based detection^18^.

**Fig. 1:**
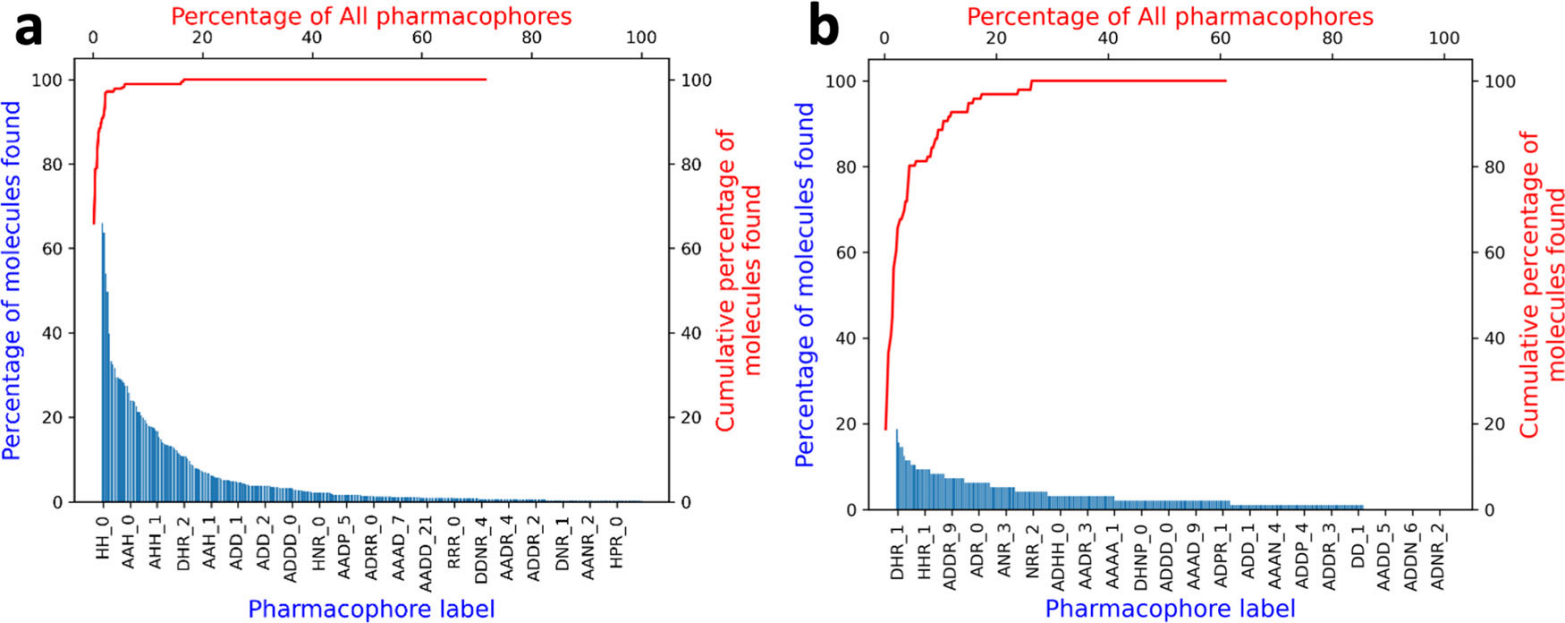
Pharmacophore coverage of fragment libraries. Relative occurrences (blue) of unique binding pharmacophores (lower *x* axis) in a commercial FFF library (**a**) vs. the SpotXplorer pilot library (**b**). The cumulative coverage of library members (red line) further highlights that almost all members of the commercial library are represented by the few most privileged pharmacophores (vs. a more even distribution for the SpotXplorer library that is about six times smaller).

Efficient library design is a central phenomenon in fragment screening since library composition impacts the chemical space and the protein interactome covered. Recently we developed a design protocol, dubbed SpotXplorer, that provides fragment libraries with maximal coverage and diversity in their representation of the unique pharmacophore patterns that were detected in over 3000 experimental protein-fragment complexes^19^. Analyzing an FFF library of a major vendor (640 compounds) reveals an uneven distribution of experimentally validated and evolutionary conserved fragment-binding pharmacophores (Fig. 1a)^19^. By contrast, a pilot library of 96 SpotXplorer fragments provides balanced coverage of the unique fragment-binding pharmacophores (Fig. 1b). The pilot library has been successfully validated against both conventional (protease and GPCRs) and challenging targets (histone methyltransferase SETD2 and SARS-CoV-2 viral targets) using biochemical and X-ray screening.

The specificity of fragment binding has been addressed in multiple studies. An analysis of 35 fragment screening campaigns of Novartis concluded that 63% of the screened fragments had never been observed as hits, while a smaller number of privileged fragments were found to be active on more than one target^20^. Similarly, many fragments that appear in crystallized complexes in the Protein Data Bank (PDB) have multiple, seemingly unrelated protein targets^21^. Interestingly, the analysis of the available X-ray structures suggested these fragments form specific binding interactions^22^. In line with these observations, we found that most fragments represent more than one unique pharmacophore and furthermore, multiple sites might recognize the same pharmacophore(s). Consequently, individual fragments can bind to diverse binding sites having the key recognition elements represented by the actual fragment pharmacophore. Considering the key objective of fragments screening as the identification of a potential binding site and a viable chemical starting point, specificity at the fragment level is not a strict requirement. In fact, in FBDD settings, binding specificity is typically ensured by the interactions formed upon the optimization of the initial fragment.

Maximizing the coverage of the experimentally validated set of binding pharmacophores at the fragment level, here we report a pharmacophore-optimized photoaffinity library (PhP) containing 100 fragments with diverse binding pharmacophores, equipped with a diazirine-type photoaffinity tag. We have characterized the library by screening against three benchmark proteins including carbonic anhydrase II, myoglobin and lysozyme. Next, we employed the library against three oncological targets with decreasing tractability: the first bromodomain of the BRD4 enzyme (BRD4-BD1) with multiple known ligands, the oncogenic KRas^G12D^ GTPase with a few structurally related inhibitors, and the *N*-terminal domain of the STAT5B transcription factor, which has no reported ligands. Binding events were first confirmed by mass spectrometry and the exact site of labeling was identified after enzymatic digestion. The identified binding sites were characterized by X-ray crystallography, HSQC NMR experiments and modeling. Our efforts revealed that pharmacophore-optimized photoaffinity fragments map the available binding sites effectively, identifying yet unexploited, tractable sites on challenging drug targets and provided viable chemical starting points for small molecule drug discovery efforts.

## Results

### Library design, synthesis, and screening

The PhP library was compiled by the SpotXplorer technology^19^ providing the optimal coverage of the unique fragment-protein binding pharmacophores as its main objective. We have recently found that experimental fragment-binding modes are represented by a limited set of 425 unique binding pharmacophores with up to four features, including H-bond donors/acceptors, positively/negatively charged groups, aromatic rings and hydrophobic groups. After suitable ligand preparation, commercial fragment collections can be utilized for assembling small fragment libraries that provide optimal coverage against this set of experimentally validated and evolutionary-conserved unique pharmacophores and can thus be used in target-agnostic screening campaigns.

**Fig. 2:**
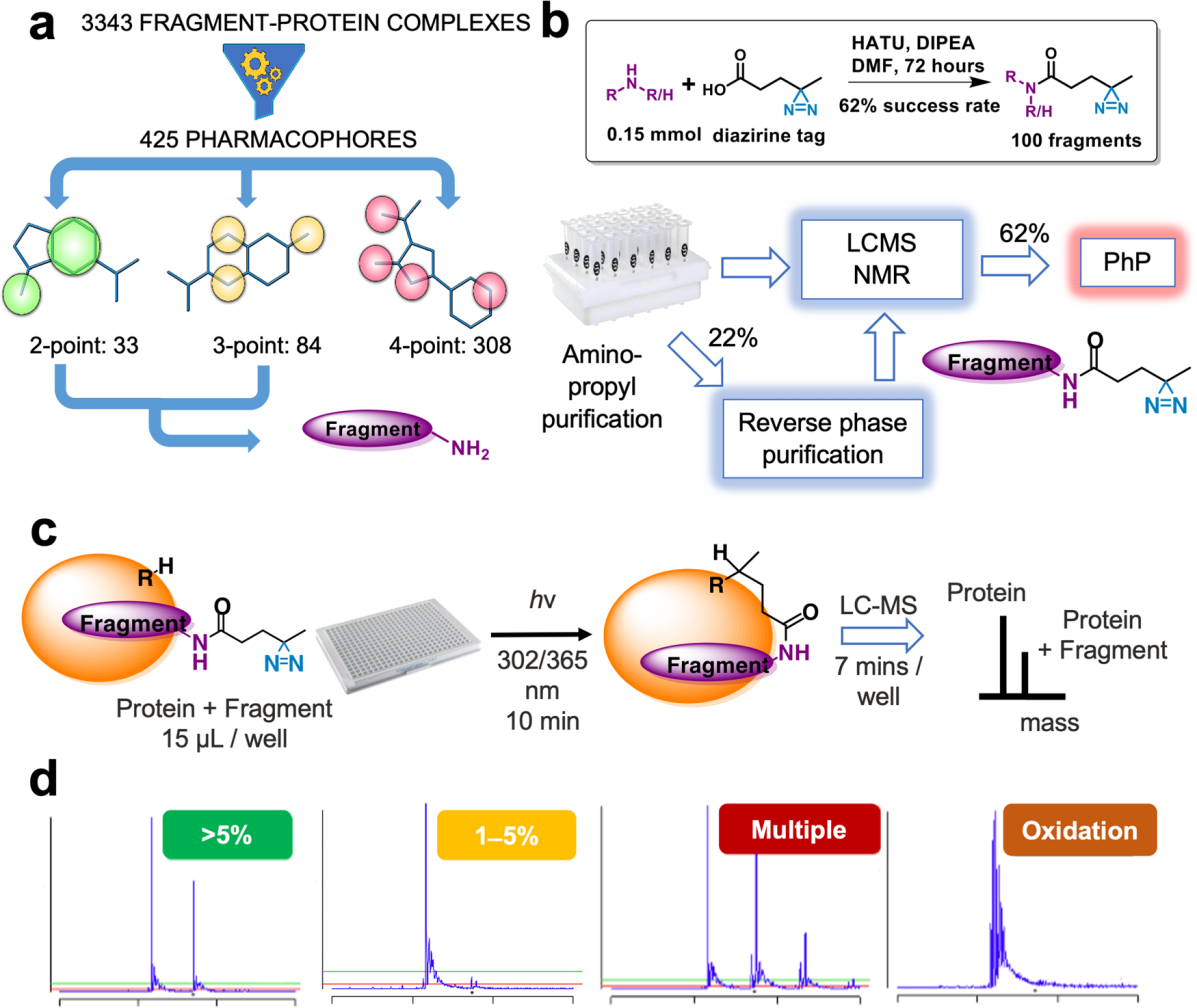
Concept, synthesis, and general screening workflow of the pharmacophore-optimized photoaffinity (PhP) library. **a** Primary amines from the Enamine collection were compiled with the SpotXplorer workflow to optimize diversity and coverage against 117 unique 2- and 3-point fragment binding pharmacophores. **b** HATU coupling in plate-based parallel synthesis and subsequent purification yielded 100 (out of 160) fragments equipped with a diazirine tag. **c** The screening paradigm employs 10 min of irradiation with the protein target and subsequent MS detection of the anchored protein-fragment complex. **d** Screening results were categorized by the analysis of their mass spectra as strong binders (>5% labeling), weak binders (1–5% labeling), multiple binders (multiple labeling), oxidized samples (having only protein +16 or multiplied peaks) and non-binders (<1% labeling) samples showing no change in the MS spectra.

To compile the PhP library, we have utilized the Enamine primary amine collection, using the primary amine group as the attachment point for the photoreactive diazirine unit. Briefly, protomers and conformers of the available amines were screened against the unique pharmacophores, and selected with a diversity picker based on the pharmacophore sets that they represent. Since fragments typically have small sizes and limited pharmacophore elements^23^ that form only a few characteristic interactions with their targets^24^; we selected a minimal set of fragments covering the largest possible fraction of experimentally validated 2- and 3-point binding pharmacophores. During library preparation, we disregarded the primary amine function as a potential pharmacophore feature (as it served the purpose of the attachment point for the diazirine unit), and we discarded any molecules with multiple primary amine groups, as well as pan-assay interference compounds (PAINS)^25^. A set of 160 compounds was assembled to provide optimal pharmacophore coverage (88% and 83%) and diversity over the 117 unique 2- and 3-point pharmacophores, respectively (Fig. 2a).

The 160 amines representing the set of experimentally validated fragment pharmacophores were coupled with the free carboxyl group of the photoaffinity tag using HATU coupling in a plate-based parallel synthesis setup. Products were subjected to purification and were analyzed by plate-based LC-MS and NMR that confirmed 100 photoaffinity labeled fragments, representing a 62% synthesis success rate (Fig. 2b). The final PhP library covers 88% and 75% of the experimentally validated 2- and 3-point pharmacophores, respectively.

Using a plate-based format with one fragment per well, each fragment was incubated with the target proteins for 15 min to allow for the formation of non-covalent interactions. The plate was then irradiated at 302 or 365 nm for 10 min to enable the bound fragments to crosslink to the protein^26^. Finally, the results were directly analyzed from the plate using intact protein mass spectrometry by observing the mass additions that correspond to successful labeling (Fig. 2c, for labeling % values see Supplementary data). The library was first characterized against three benchmark proteins like carbonic anhydrase (CA), lysozyme (Lyo), and myoglobin (Myo). These proteins were labeled by 17, 16, and 23 fragments, respectively. This result showed a consistent efficiency of the library compared to the larger set (556 PhaBits) of Grant et al. that showed 47% labeling efficiency for Lyo, 18% for CA and <3% for Myo^17^.

Next, we screened the library against three oncology targets with decreasing tractability. Among the therapeutically relevant targets, BRD4-BD1 represents a conventional target having 6000+ ligands available in the ChEMBL database. We considered the oncogenic KRas^G12D^ mutant as a challenging target with a few ligands reported recently in the literature. Finally, we screened the PhP library against the *N*-terminal domain (NTD) of the transcription factor STAT5B that has no known ligands reported to date. Hits were detected by two different MS setups: for BRD1-BD4 and KRas^G12D^ we used a high-end LC-MS-TOF spectrometer, while STAT5B-NTD was screened on a readily available standard UHPLC-MS system. Hits were classified by the analysis of the spectra following criteria on the labeling pattern (Fig. 2d).

From all targets, BRD4-BD1 was the most vulnerable (30 fragments labeled >1%) followed by STAT5B-NTD and KRas G12D (26 and 25, respectively). There were 44 fragments in the library that did not label any of the proteins more than 1% and further 19 did not reach 2%. This is in line with Grant et al. reporting that majority of the 556-membered library did not crosslink to any targets. In their investigation 10 PhaBits out of 556 (1.8%) labeled six proteins, while 58 (10.4%) labeled at least 4 targets^17^. Among our most privileged fragments, two have labeled four proteins each (**PhP018** and **PhP060**), while four have labeled two targets in higher than 10% amount (**PhP038,** **PhP034,** **PhP071,** **PhP087**). These might represent privileged scaffolds that could fit into several protein pockets, or be due to nonspecific crosslinking through lipophilic interactions with the protein or formation of long lifetime carbenes^17^. Importantly, all proteins had privileged pharmacophores that labeled their targets in a significantly higher amount than others (see Supplementary Table S1 and the corresponding discussion). CA was mostly preferred by **PhP003** (4.7%) that slightly labeled the others (<1.1% for each). BRD4-BD1 was mostly preferred by **PhP053** (6.7% vs. <2%). In the case of KRas, **PhP048** and **PhP012** were the most selective compounds (29.4% and 15.8% vs. <3%, respectively). **PhP092,** **PhP001** and **PhP088** preferred Lyo (35.6%, 19.2% and 4.8% vs. <1%), while Myo was targeted selectively only by **PhP082** (52.9% vs. <0.5%). STAT5B-NTD was most selectively labeled by **PhP040,** **PhP077,** **PhP065** and **PhP097** (75.0%, 20.0%, 6.5% and 5.7% vs. <3%, respectively).

Usually, >20% labeling efficiencies are accompanied with oxidation, protein degradation or other side reactions. We identified six fragments that showed labeling efficiencies in the 1–13% range consistently over all protein targets (**PhP099**: 5.2 ± 4%, **PhP072**: 4.4 ± 4.3%, **PhP080**: 3.6 ± 2.2%, **PhP037**: 3.4 ± 3.8%, **PhP098**: 3.3 ± 3.1%, **PhP035**: 1.5 ± 1.2%). From this set, **PhP037** and **PhP098** differ only in a substituent on the phenyl ring connected to imidazole and the labeling pattern is very similar e.g., KRas G12D is labeled in 11.2% and 9.6%, BRD4-BD1 in 4.8% and 4.3%, while STAT5B-NTD in 1.4% and 2.3%. **PhP072** might also be highlighted as a consistently labeling fragment with a pyridoxamine core structure. In the following subsections, we present our results on the three oncological targets in more detail.

### Fragment hits against BRD4, a tractable target

As a tractable protein target with thousands of reported ligands, we have first screened the PhP library against the first bromodomain of BRD4 (BRD4-BD1). BRD4 is a member of the Bromodomain and Extraterminal protein family, and has important roles in the expression regulation of oncogenes, as well as the maintenance of genome stability^27^. As such, BRD4 is an important oncotarget for small-molecule intervention, with an accessible and druggable acetyl-lysine (AcK) binding site^28^.

**Fig. 3:**
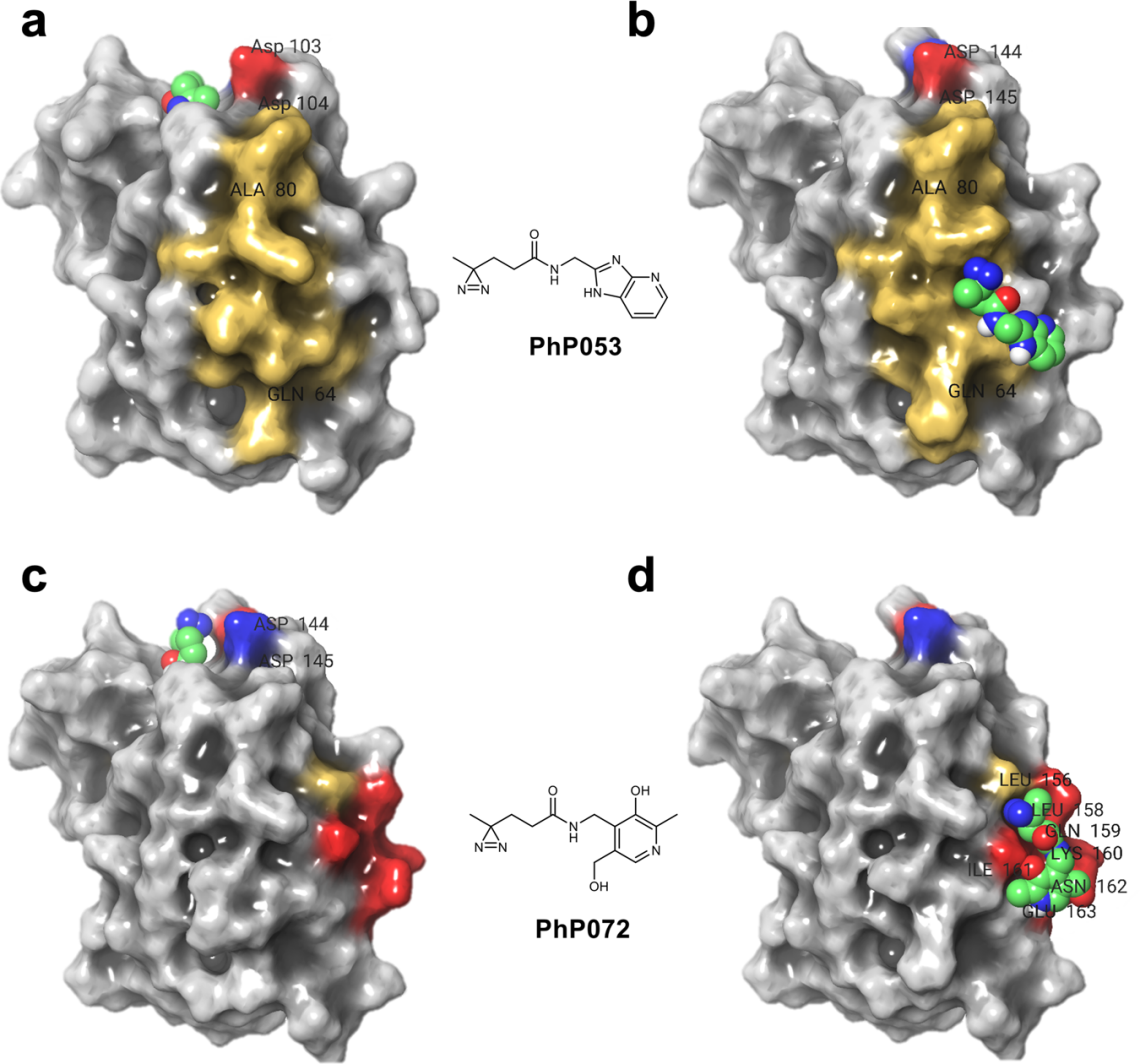
Binding sites identified on the BRD4-BD1 surface revealed by screening the PhP library. Experimental (**a**) and modeled (**b**–**d**) binding poses of two verified hits against the primary binding site (left column) vs. other locations (right column) on BRD4-BD1. In the newly identified, surface-exposed sites, **PhP053** binds via multiple H-bond and π-π contacts to the Y65 and K160 sidechains, while **PhP072** occupies a neighboring site by multiple H-bonds towards K160 and E163. Ligands are represented as spheres, labeling sites (as determined by peptide mapping) are colored red (exact site found) or yellow (approximate site found along a longer sequence) on the protein surface. The binding mode of **PhP053** vs. the primary site was solved by X-ray crystallography and has been deposited into the PDB database with the accession code 8Q34.

Intact MS screening against BRD4-BD1 has identified five fragment hits with >1% target labeling, and we could verify the binding sites of two of them by peptide mapping performed by LC-MS/MS following tryptic digestion (Fig. 3). One hit, **PhP053** was successfully co-crystallized with the protein, providing further evidence of binding in the primary (AcK) binding site. Other binding modes, including one in a secondary site for **PhP053**, as well as the AcK site and a third site (residues 156-163) for **PhP072** were modeled with induced fit docking, considering the sites of labeling, as verified by peptide mapping. These efforts revealed that the PhP library could identify hits against the main binding site of a tractable target, and in addition, some hits could identify yet unreported orthogonal, surface-exposed binding sites, facilitating the design of allosteric ligands.

### Fragment binders for KRas^G12D^, a challenging target

KRas^G12D^ represents a challenging drug target previously considered as non-druggable^29^. Oncogenic KRas mutants attracted considerable interest in recent years and drug discovery efforts identified multiple fragments and drug-like compounds having affinity against KRas^G12D^. In fact, several distinct binding sites were successfully targeted by recent efforts^30^, including the Switch II pocket^31^, the SOS interface^32,33^ and the interface of the Switch I/II regions^34,35^. To our current knowledge though, there are no reported inhibitors targeting the RBD and dimerization interfaces, or the Switch I region^36^. Given the fact that KRas has relatively shallow, solvent-exposed pockets, we hoped that any labeling would be an indication of a high-affinity fragment binding site of the target.

**Fig. 4:**
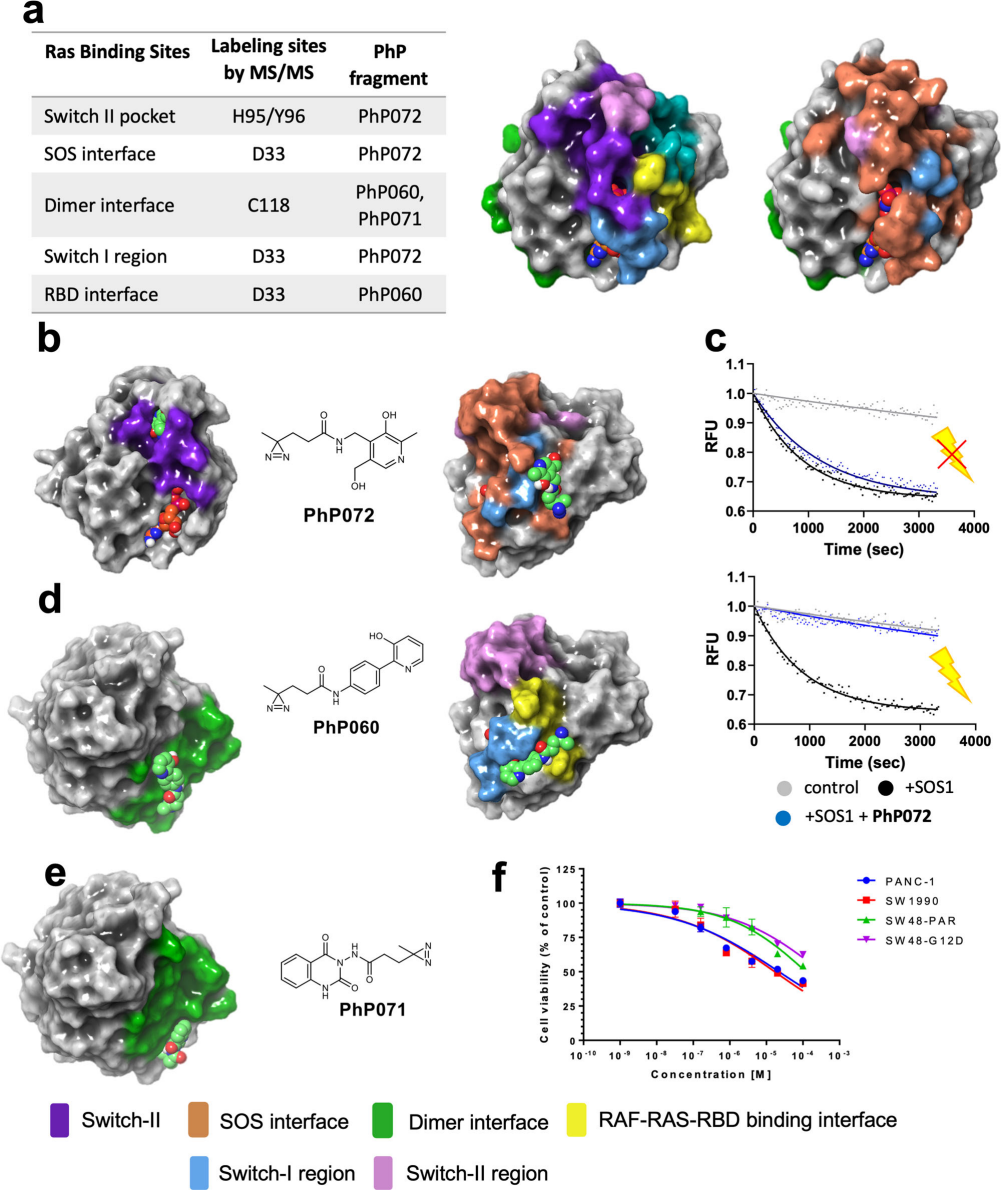
Binding sites identified on the KRas^G12D^ surface revealed by screening the PhP library. **a** Experimentally validated labeling sites of fragment hits on the surface of KRas^G12D^ **b PhP072** binds to distinct sites in the Switch-II pocket and SOS interface. **PhP072** forms H-bond interactions with D69 and E63 in the Switch-II pocket, and with S39, T35, E37. **c** In the KRas-SOS exchange assay, SOS1 displaces MANT-labeled GDP from KRas (black curve). Addition of **PhP072** (blue curve) has little effect prior to irradiation (top), but fully restores KRas function to the control level (gray curve) upon irradiation (bottom). **d PhP060** binds to distinct sites on the dimerization and RBD interfaces via H-bonds to D33, H27, T35, and a π–π interaction to H27. **e PhP071** binds to the dimerization interface via multiple H-bond interactions with R123 and T127. **f PhP072** shows antiproliferative effect on KRas-dependent pancreatic and colon cancer cells (blue: PANC-1, red: SW1990, green: SW48-PAR, purple: SW48-G12D). Small molecules are shown as spheres: fragments with green carbons, GDP with orange carbons. In (**c**), each data point and the fitted kinetic curves are shown. In (**f**), data are presented as mean values ± SD, calculated from two biologically independent samples, each with three repetitions. Source data are provided in the Source Data file.

Intact MS screening of the PhP library resulted in crosslinking for 11 compounds. Their sites of labeling were investigated by mass spectrometry after tryptic digestion, while the contacting amino acids were identified by HSQC NMR. Combining these orthogonal experiments, we identified the respective binding sites of the three confirmed hits with induced fit docking using experimental information available from MS-based peptide mapping and HSQC NMR. This analysis revealed that PhP fragments have identified multiple allosteric pockets or protein-protein interaction contact surfaces that were not yet targeted by small molecules (Fig. 4).

Following up on the three fragment hits, we have proven by a KRas-SOS exchange assay that **PhP072** fragment binding at the Switch I, II and SOS interfaces influences KRas function (Fig. 4c). Consistently with the generally low affinity of fragment-sized ligand upon non-covalent binding, **PhP072** could restore KRas function only upon irradiation (Fig. 4c, bottom panel). In contrast, **PhP071** and **PhP060** fragments that bind at the RBD and dimerization interfaces have no effect on the nucleotide exchange, before or after irradiation.

Encouraged by the positive labeling and functional results, we have undertaken further efforts to assess the utility of the fragment hits in living cells. The dependency of the applied cell lines for the protein targets was determined by obtaining CRISPR knockout sensitivity data from https://depmap.org, and the combined CRISPR sensitivity values (DepMap 22Q2 Public+Score, Chronos) were downloaded (Supplementary Fig. S1). First, we have tested the effect of **PhP072** against a range of KRas-dependent cancer cells including PANC-1 (human pancreatic cancer, KRas^G12D^), SW1990 (human pancreatic cancer, KRas-G12D), SW48-PAR (human colon cancer; parental cell line KRas^wt^), and SW48-G12D (human colon cancer; heterozygous knockin of the KRas^G12D^ activating mutation) cancer cell lines. **PhP072** showed antiproliferative activity against all the KRas-dependent cell lines with IC_50_ values of 17.2 and 22.6 µM against SW1990 and PANC-1, respectively.

### Fragment hits against STAT5B NTD, an unliganded target

Finally, we aimed to identify viable fragment starting points against signal transducer and activator of transcription 5B (STAT5B), an important transcription factor mediating or even driving cancer progression through hyperactivation or gain-of-function mutations^37^. While there is great interest in its small molecule targeting, no direct STAT inhibitor has reached the market yet. Furthermore, the few ligands of STAT5B reported so far are all targeting its highly conserved SH2 domain^38^. Therefore, targeting the yet unliganded *N*-terminal domain (NTD) represents an approach for the direct inhibition of STAT5B function that would open a possibility for pharmacological intervention to cancer progression. Recent works to characterize and target the *N*-terminal domain of STAT3, including its published X-ray structure^39^ and a high-throughput virtual screen^40^, provide a solid basis for this effort.

**Fig. 5:**
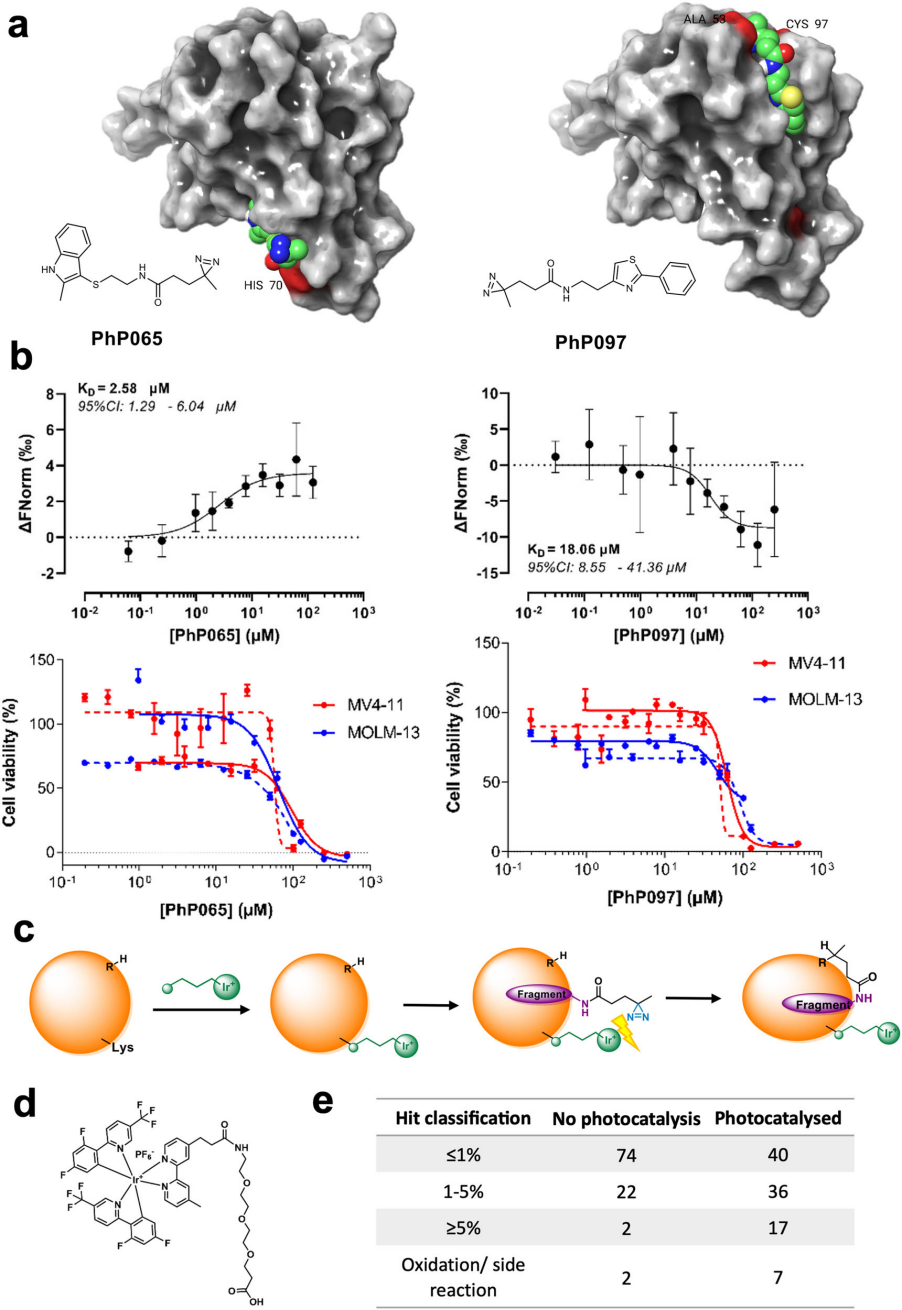
Results of screening the PhP library against the STAT5B N-terminal domain. **a** Structures and predicted binding modes of **PhP065** and **PhP097** against the dimerization interfaces of the STAT5B *N*-terminal domain. **PhP065** binds close to the cavity formed in the handshake dimer interface by H-bonds with the Q36 and K70 residues. **PhP097** binds at the tip of the α6 and α7 helices next to the Ni^2+^-mediated tetramer interface with an H-bond to the T58 sidechain and further hydrophobic contacts. **b** In microscale thermophoresis, **PhP065** and **PhP097** bind directly to STAT5B-NTD, with *K*_d_ values of 2.58 and 18.06 µM, respectively (black curves). Furthermore, the fragments inhibit the viability of the established leukemia cell lines MV4-11 (red) and MOLM-13 (blue) with IC_50_ values of 76.15 and 100.1 µM (**PhP065**), and 56.62 and 68.22 µM (**PhP097**), respectively. **c** In our modified workflow, the iridium-based photocatalyst Ir-G2-PEG3-COOH is first attached to the target protein. After initial non-covalent binding, the crosslinking of the photoaffinity-labeled fragments is enhanced by Dexter-energy transfer from the photocatalyst. **d** Structure of the photocatalyst Ir-G2-PEG3-COOH^41^. **e** Application of the photocatalyst results in higher labeling efficiency and, by transition, a higher number of primary hits (>1% labeling). In the *K*_d_ plots, data are presented as mean values ± SD, calculated from two biologically independent samples, each with three repetitions. In the cellular IC_50_ plots, data are presented as mean values ± SEM, separately for two biologically independent samples, each with three repetitions. Source data are provided in the Source Data file.

After successful validation against BRD4-BD1 and KRas^G12D^, we introduced two methodological improvements in the screening process applied against STAT5B-NTD. First, we replaced the high-end LC-MSMS-TOF platform by a readily available UPLC-MS system that might broaden the user community. Second, we attempted to increase the sensitivity of screening by improving the labeling efficiency of PhP fragments. We have noticed that the labeling efficiency of the diazirines is usually around or less than 5% that limits the sensitivity of detecting bound fragments. Therefore, we synthesized an iridium-based photocatalyst (Fig. 5d) and designed a methodology to conjugate the catalyst to the target protein in water using the activating agent BOP, or ((1H-benzo[d][1,2,3]triazol-1-yl)oxy)tris(dimethylamino)phosphonium hexafluorophosphate)^41^. During the screening of photoreactive fragments, the catalyst can activate the diazirine moiety by Dexter-energy transfer to result in increased labeling efficiency (Fig. 5c)^41–43^. (To note, the small size of the N-terminal domain and the long-range effect of Dexter-energy transfer allow for the activation of the photoaffinity tag practically anywhere on the STAT5B-NTD surface, see Supplementary Fig. S2.)

Intact MS screening has identified 24 hits against STAT5B-NTD. As follow-up, the labeling site and efficiency of the hits were analyzed by mass spectrometry, and their prospective binding modes were predicted by ligand docking. Since the labeling sites are close to the two known dimerization interfaces of STAT N-terminal domains, we presume that the hits can inhibit the formation of the “handshake dimer” or the Ni^2+^-mediated tetramer of the *N*-terminal domain (Fig. 5a)^39^. This was further verified on the two best fragment hits for the two mentioned sites, **PhP065** and **PhP097** respectively, which provided over 5% labeling and no side reactions, even without the use of the photocatalyst. Here, microscale thermophoresis measurements that revealed direct binding of the two hits to STAT5B-NTD even without irradiation, with *K*_d_ values in the low micromolar range (Fig. 5b, black curves). To investigate the translational potential of the resulting fragment hits, we have tested the effects of **PhP065** and **PhP097** on the viability of two established leukemia cell lines, MV4-11 and MOLM-13 (see CRISPR knockout sensitivities in Supplementary Fig. S1). Here, the hit fragments exhibited cellular IC_50_ values in the mid-micromolar range (Fig. 5b, red and blue curves), further verifying them as viable starting points for hit elaboration. Finally, we have shown significantly increased labeling efficiencies and more primary hits in case of the photocatalyzed intact MS screening of PhP-fragments, confirming the usefulness of the improved protocol (Fig. 5e).

## Discussion

We introduced a fragment screening concept using pharmacophore-optimized photoaffinity fragments (PhPs), by combining the advantages of two recent approaches reported by our groups^17,19^. The advantages of PhP are apparent in comparison to SpotXplorer, as the PhP library readily offers the capacity to experimentally detect the relevant binding sites of the discovered hits, in a platform that is more accessible to a wider community. By comparison, in validating the SpotXplorer technology against targets that were new at the time, specifically the SARS-CoV-2 viral targets 3CLPro and NSP3, we needed a state-of-the-art synchrotron facility to obtain high-resolution crystal structures of the confirmed hits. Additionally, our current results have illustrated that there are distinct cases regarding the available binding site(s). In some cases, we can infer analogies based on previously reported ligands (BRD4-BD1). However, for the more elusive target KRas^G12D^, there are numerous binding sites available, so the direct experimental mapping provides invaluable and readily available guidance for hit-to-lead follow-up at an early stage. Finally, for the unexplored STAT5B-NTD target, we have gathered first evidence for targeting the dimerization interface, as verified by MS screening. We should highlight that the orthogonal, secondary binding assays (X-ray crystallography for BRD4-BD1, HSQC NMR for KRas^G12D^ and MST for STAT5B-NTD) were carried out without irradiation, verifying the affinity of the fragments towards the respective targets via a non-covalent mechanism of action. Additionally, the detected affinities against KRas^G12D^ and STAT5B-NTD (IC_50_ and K_d_ values in the 2–20 µM range) exceeded our expectations toward fragment hits. These promising on-target affinities prompted us to test the best fragments against KRas^G12D^ and STAT5B-dependent cell lines, respectively. Although their observed cellular activity supports that the fragments interact with the target in cells, but further studies will be required to ascribes its functional effects to on-target binding given their promiscuity. In fact, during the revision process, Offensperger et al. published a large-scale chemoproteomics study, using 407 diazirine-tagged fragments^44^. From this set, we identified compounds with notable structural similarities to our fragment hits (Supplementary Table S2) that labeled 10–300 proteins in cells.

In comparison to the original PhABits screening platform, the main advantages of the PhP library are the higher hit rates and, by design, its better coverage of the unique fragment-binding pharmacophore patterns. For the highly tractable BRD4-BD1 target, both libraries provided fragment hits against its primary binding site (nine primary hits for PhABits and five for PhP). Notably though, the smaller number of hits provided about the same coverage of the pharmacophore space of the previously reported, fragment-sized BRD4-BD1 inhibitors (58 fragments downloaded from the ChEMBL database^45,46^ with 10–16 heavy atoms and a pChEMBL value ≥3, representing millimolar or better activity). Specifically, 63 and 59% of the unique pharmacophores represented by at least one literature inhibitor were recovered by the nine PhABit and five PhP hits, respectively (vs. a priori expectations of 16 and 9%, assuming an even distribution of pharmacophores). The smaller number of PhP hits can be rationalized by their optimized pharmacophore coverage as compared to PhABits. The similar coverage of known pharmacophores, however, was achieved by significantly fewer PhP fragments as PhABits (100 vs. 556 compounds). Comparing the screening efficiency of PhABit and PhP libraries revealed three times higher hit rate for the latter platform (1.6% vs 5%, respectively). In addition, each of the two hits that were further verified by peptide mapping, **PhP053** and **PhP072**, have labeled an alternative binding site in addition to the main one. The difference is even more apparent for KRas^G12D^, where the three verified PhP hits have labeled five different binding sites, in comparison to one site labeled by the four hits resulting from the larger PhABits library. This is a direct consequence of the SpotXplorer design, that optimized the library towards the best possible coverage of the diverse pharmacophore patterns observed in experimental protein-fragment complexes. Furthermore, screening the PhP library provided a more than seven times higher hit rate (5% vs. 0.7%) against this challenging target. Therefore, the PhP hits explored a higher diversity of potential binding sites with higher screening efficiency as compared to the PhABit hits.

Finally, we highlight two important methodological developments. First, we adapted the screening technology to conventional LC-MS platforms, in line with recent efforts to democratize MS-based fragment screening approaches for academic labs^47–49^. Second, we improved its sensitivity by a recently reported bioconjugated photocatalyst that improves the efficiency of photoaffinity labeling. This approach can eliminate the bottleneck of limited labeling efficiency in screening low-affinity fragments, and to our knowledge is the first successful attempt at attaching this photocatalyst to a full protein. Electrophiles targeting a specific sidechain, such as cysteine, typically have a high labeling efficiency, but a limited proteome coverage, as the targeted sidechain must be present in the binding site. By comparison, photoaffinity tags offer a far superior proteome coverage by design, but this comes at a cost of lower modification yields, cf. our classification of primary hits with >5% labeling rates (Fig. 2d). Ultimately, this is a key factor regarding the detection sensitivity of the platform. One possibility for improving modification yields is using different photoreactive functionalities. However, a higher reactivity might come at a cost in other properties, such as a lower aqueous stability^50^. In the present work, we have followed a different train of thought by boosting the labeling efficiency of our photoaffinity-tagged fragments with a bioconjugated iridium-based photocatalyst. This approach doubled the number of hits identified against the previously unliganded and challenging transcription factor target, STAT5B-NTD (Fig. 5c). It should be noted that in addition to the much-improved labeling efficiency we observed slightly higher number of side reactions such as oxidation or water elimination. These reactions might limit the use of certain moieties; however, the covered pharmacophore information can be preserved by choosing more photoresistant scaffolds. These developments in the screening technology improved the detection sensitivity of the platform especially on low-end LC-MS platforms.

In conclusion, combining the advantages of two fragment screening platforms developed at our labs we introduced pharmacophore-optimized photoaffinity fragments that provides higher hit rates and better exploration of available protein binding sites even for challenging or unliganded targets. Improving the sensitivity of hit detection by a bioconjugated photocatalyst and transforming the screening platform to readily available LC-MS systems, we widened the scope of potential users. Our work can also be considered as an example of academia-industry collaborations that facilitate drug discovery efforts and therapeutic innovations via new tools available for the wider community.

## Methods

### Library design

The library was designed as reported in our recent work^19^. Briefly, pharmacophore models were extracted from PDB structures with the ePharmacophore module of the Schrödinger software suite (version 2017-4), and clustered with scipy (version 1.0.1). Ligand preparation, conformer generation and pharmacophore screening were carried out with the Epik, Macromodel and Phase modules of the Schrödinger software suite, respectively (version 2022-4).

Fragments in the Enamine primary amine collection were filtered for size (10–16 heavy atoms), primary amine group count of exactly one, and the absence of PAINS moieties^25^. Candidate fragments were screened against, and annotated with the full set of 2-, and 3-point pharmacophore models and stored as fingerprints with bits for pharmacophores present set to 1. We have used a diversity picker to select 160 fragments with maximized coverage and diversity across the 117 bit positions (pharmacophores). The final set of successfully synthesized 100 PhP fragments provided coverages of 88% and 75% for the 2- and 3-point pharmacophores, respectively. SMILES codes, images and pharmacophore fingerprints of the PhP fragments are included in Supplementary Data 1.

### General procedures for synthesis and compound characterization

**Scheme 1:**
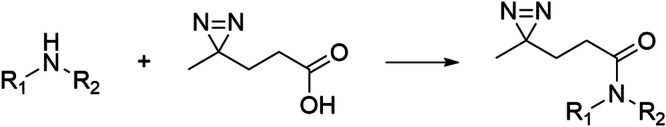
General procedure for attaching the diazirine tag onto amine-containing fragments. Amide coupling with the HATU (Hexafluorophosphate Azabenzotriazole Tetramethyl Uronium) coupling reagent was utilized to award the photoaffinity-tagged fragments of the PhP library.

PhP fragments were synthesized according to the general procedure outlined in Scheme 1. 3-(3-Methyl-3H-diazirin-3-yl)propanoic acid (0.025 g, 0.195 mmol) was dissolved in *N*,*N*-dimethylformamide (DMF), 1-[bis(dimethylamino)methylene]-1H-1,2,3-triazolo[4,5-b]pyridinium 3-oxid hexafluorophosphate (HATU, 0.071 g, 0.188 mmol) and DIPEA (0.105 mL, 0.600 mmol) were added. The reaction mixture was stirred for 15 min at room temperature. 0.15 mmol amine was added, then the mixture was stirred at RT for 16-72 h covered in tin foil, and progress was assessed by LCMS.

After 16–72 h the reactions were processed as follows:

- DMF in the reaction mixtures was removed in a Genevac. The crude product was dissolved in chloroform (0.4 ml) and loaded onto a 1.0 g NH_2_-Isolute SPE column, pre-equilibrated with chloroform for 2 column volumes (CV’s). The product was eluted off the column using 2.5 mL of 10% methanol in ethyl acetate. This was repeated with another 2.5 mL of 10% methanol in ethyl acetate. The eluent was collected for each reaction in pre-weighed T-vials and dried under a stream of nitrogen in the Radleys blowdown apparatus to obtain a product.
- The reaction mixture was loaded to a prep-HPLC column and purified with a linear gradient from 5 to 100 vol% MeCN in water containing 0.1 vol% formic acid over 15 min. The solvent was then evaporated.

^1^H NMR was recorded in DMSO-d_6_, CDCl_3_ CD_3_CN or D_2_O solution at room temperature, on a Varian Unity Inova 500 (Varian, Palo Alto, CA, USA) (500 MHz) and on a Varian 300 spectrometer (300 MHz), with the deuterium signal of the solvent as the lock. Chemical shifts (*δ*) and coupling constants (*J*) are given in ppm and Hz, respectively, and the spectra are collated in Supplementary Data 2.

HPLC-MS measurements were performed using a Shimadzu LC-MS-2020 device equipped with a Reprospher-100 C18 (5 µm; 100 × 3 mm) column and positive-negative double ion source (DUIS±) with a quadrupole MS analyzer in a range of *m/z* 50-1000. Samples were analyzed with gradient elution using eluent A (0.1% formic acid in water) and eluent B (0.1% formic acid in acetonitrile). Flow rate was set to 1 mL/min. The initial condition was 5% B eluent, followed by a linear gradient to 100% B eluent by 1 min, from 1 to 3.5 min 100% B eluent was retained; and from 3.5 to 4.5 min back to initial condition with 5% B eluent and retained to 5 min. The column temperature was kept at room temperature and the injection volume was 1–10 µL. Purity of compounds was assessed by HPLC with UV detection at 254 nm; all tested compounds were >95% pure.

High-resolution mass spectrometric measurements were performed using a Q-TOF Premier mass spectrometer (Waters Corporation, Milford, MA, USA) in positive or negative electrospray ionization mode. Reactions were monitored with Merck silica gel 60 F^254^ TLC plates (Darmstadt, Germany). All chemicals and solvents were used as purchased from commercial suppliers. The column chromatography purifications were performed using Teledyne ISCO CombiFlash Lumen+ R_f_. For buffer media exchange, a GE Healthcare PD SpinTrap™ G-25 desalting column was used.

Detailed analytical results are included in Supplementary Note 1 for each compound.

### Sample preparation for STAT5B-NTD illumination and photocatalysis experiments

STAT5B protein in 7 µM concentration in pH 7.4 PBS buffer was pre-incubated with 0.17 µL PhP (in 100 mM DMSO solution) for 1 h at room temperature in dark. Irradiation was carried out at 4 °C for 10 min using 365 nm UV lamp.

The synthesis of short- and long-linker Ir-G2 photocatalysts was started from dMebppy^41^. First, we synthesized Gen 2 Iridium catalyst (cpd. 13 in ref. ^41^) that was not effective for labeling STAT5B-NTD, presumably due to the short propionic acid linker. However, the carboxylic acid analog with a longer PEG3 linker (Ir-G2-PEG3-COOH, or cpd. 17 in ref. ^41^) successfully labeled the protein target in the presence of BOP (but not of other activating agents like HATU, HCTU, TSTU or PyAOP) with a 27% yield (Supplementary Fig. S3).

For the photocatalysis experiments, 1.4 µL photocatalyst (in 1.4 mM DMSO solution, 10 eq) was pre-incubated with 1.5 µL coupling reagent (0.75 µL, 5.6 mM EDC (20 eq)+0.75 µL 4.2 mM NHS and 1.5 µL 2.1 mM (15 eq) BOP for 30 min in dark. 30 µL STAT5B-NTD (in 7 µM concentration) was added and incubated further at room temperature overnight or at 37 °C for 1 h in dark. After addition of 0.14 µL PhP (in 100 mM DMSO) the solution was further incubated in dark for 1 h. Irradiation was carried out using 450 nm LED lamp (7.6Vx0A) for 10 min at 4 °C.

### Intact mass spectrometry (MS) screening

The PhP library was screened against BRD4-BD1 and KRas^G12D^ as follows. Using a Labcyte Echo® 555 Liquid Handler, 150 nL of fragment solution (20 mM) was transferred into a Greiner 384 low-volume plate (white) to prepare the probe plate. The probe plate was placed on ice. 15 μL of protein stock solution (1 μM BRD4-BD1 (GSK/GenScript) or KRas^G12D^ (GSK/GenScript) in PBS buffer) was dispensed into wells containing fragments. The plate was left on ice for 15 min for incubation, then irradiated at 302 nm for 10 min, and centrifuged (1000 rpm, 1 min) to remove any bubbles. The plate was then analyzed by LCMS-TOF mass spectrometry (Agilent 1200 series liquid chromatography with Agilent Bio-HPLC PLRP-S (1000 Å, 5 µm × 50 mm × 1.0 mm, PL1312-1502) reverse phase HPLC column at 70 °C equipped with an Agilent G6224 time-of-flight (ToF), see Supplementary Note 2 for the full protocols). The deconvoluted spectra were analyzed using R Studio software. Spectra of the hits are reported in Supplementary Note 2.

For screening against STAT5B-NTD, we have used a UHPLC-MS system that consisted of a Waters ACQUITY UPLC I-Class setup coupled with a Waters ACQUITY UPLC Peptide BEH C18 Column (130 Å, 1.7 µm, 2.1 mm × 100 mm), connected to a Waters Xevo G2-XS QT-ToF instrument equipped with a Waters Z-spray ESI source. During the analysis, the column temperature was maintained at a constant 60 °C, and a sample volume of 3 µL was injected for each analysis. Data acquisition was conducted in positive ion mode within the *m/z* 100–2000 (mass-to-charge ratio) range. For the full protocol and spectra of the hits, see Supplementary Note 2.

### Binding site identification by LC-MS/MS peptide mapping—BRD4-BD1 and STAT5B-NTD

In follow-up of the intact MS screening, the fragment hits were further analyzed by a Triple TOF 5600+ hybrid Quadrupole-TOF LC/MS/MS system, after digesting the resulting fragment-protein complexes by Trypsin/Lys C mix. Data acquisition and processing were performed using Analyst TF software version 1.7.1 (AB Sciex Instruments, CA, USA). Chromatographic separation was achieved on the Discovery® BIO Wide Pore C-18-5 (250 mm × 2.1 mm, 5 μm, 300 Å) HPLC column. MS/MS spectra were obtained on the 8 most abundant parent ions present in the TOF survey scan with the Information Dependent Acquisition (IDA) mode, and peaks were evaluated with PeakView^®^ (version 2.2, Sciex) and Biologics Explorer (version 3.0.3, Sciex). The full sample preparation and data acquisition protocols, as well as the spectra of the reported hits, are available in Supplementary Note 3.

### Modeling the binding poses of fragment hits by docking

Docking calculations were performed on BRD4-BD1 (PDB ID: 7A9U^17^), KRas^G12D^ (PDB ID: 4OBE^51^) and STAT5B-NTD with the same protocol. Briefly, the relevant X-ray structures were downloaded from PDB and were prepared using Protein Preparation Wizard^52^. The structure of STAT5B-NTD was homology modeled based on the published structures of STAT3-NTD (PDB ID: 4ZIA^39^). Ligands were prepared with Ligprep^52^, and docking was performed with the Induced Fit Docking protocol of Schrödinger. For the grid box generation, the experimental results (MS labeling data and NMR shift perturbations, where available) were used. At most 20 possible binding conformations were generated in the first docking step^53^. Redocking was done into structures within a 30 kcal/mol energy window from the best structure, and within the top 20 structures overall, using the single precision (SP) method.

### Cell viability measurements

Pancreatic cancer cell lines PANC-1 (cat. no. CRL-1469), SW1990 (cat. no. CRL-2172) were obtained from ATCC (American Type Culture Collection). Genetically modified isogeneic colon cancer cell lines SW48-PAR and SW48-G12D were obtained from Horizon Discovery Ltd. PANC-1 (human pancreatic cancer, KRas^G12D^), SW1990 (human pancreatic cancer, KRas^G12D^), SW48-PAR (human colon cancer; parental cell line KRas^wt^), and SW48-G12D (human colon cancer; heterozygous knockin of the KRas^G12D^ activating mutation) cancer cell lines were cultured in Roswell Park Memorial Institute Medium (RPMI; Biosera, Nuaille, France), supplemented with 10% heat-inactivated Fetal Bovine Serum (FBS; Biosera), and with 1% Penicillin/Streptomycin (Biosera). Cells were cultured in sterile T75 flasks with ventilation cap (Sarstedt, Nümbrecht, Germany) at 37 °C in a humidified atmosphere with 5% CO_2_ in ESCO CelCulture Incubator (ESCO, Friedberg, Germany). Manipulations with the cells were performed in biosafety cabinet (laminar) ESCO Sentinel Gold class II model AC2-4E8 (ESCO).

For the evaluation of the in vitro antiproliferative activity of fragments, cell viability was determined with the MTT assay (3-(4,5-dimethylthiazol-2-yl)-2,5-diphenyl-tetrazolium bromide from Sigma Aldrich). After standard harvesting of the cells by trypsin-EDTA (Biosera), 7 × 10^3^ cells per well depending on the cell line, were seeded in serum-containing growth medium to 96-well plates and incubated. After 24 h, cells were treated with various concentrations of fragments (32 nM–100 μM), dissolved in serum-containing medium, and incubated under standard conditions. Control wells were treated with medium. Final concentration of serum was 2.5%, final concentration of DMSO was 0.2%. Treatment was for 72 h continuously.

Afterwards, MTT assay was performed in order to determine cell viability, by adding 20 μL of MTT solution (5 mg/mL in PBS) to each well and after 2 h of incubation at 37 °C, the supernatant was removed. The formazan crystals were dissolved in 100 μL of a 1:1 solution of DMSO (Sigma-Aldrich):EtOH (Molar Chemicals) and the absorbance was measured after 15 min at *λ* = 570 nm by using a microplate reader (BioTek 800TS, Agilent, Santa Clara, CA, USA). The IC_50_ values of the fragments were calculated using GraphPad Prism 6 (GraphPad Software, San Diego, CA, USA). The experiments were done in triplicate, and each experiment was repeated two times.

### BRD4-BD1 expression, purification, crystallization, and structure

BRD4 (residues 44-171, cloned into pNIC28-Bsa4 using LIC cloning, SGC ID: BRD4A-c001) was produced from *E. coli* (BL21) RR in TB media (Formedia) with expression induced with 1 mM IPTG when OD600 = 0.6 was reached at 18 °C overnight. The cells were lysed (50 mM HEPES, 500 mM NaCl, 5% glycerol) by sonication, purified by IMAC using Talon resin (50 mM HEPES, 500 mM NaCl, 5% glycerol, 300 mM imidazole), tag removed with TEV cleavage overnight at 4 °C and polished using gel filtration with Superdex 75 column. Purified protein in (50 mM HEPES, 500 mM NaCl, 5% glycerol) was concentrated to 5.6 mg/mL and crystallized in 150 nL drop in 1:2 protein: reservoir solution ratio in 20% PEG6000, 10% ethylene glycol, 0.1 M HEPES pH 7.0 and 0.2 M sodium chloride at 277 K using sitting drop vapor diffusion. Crystals formed after 28 days. **PhP053** (100 mM, dissolved in MeOH, not DMSO) was diluted 10 times with reservoir solution added to the crystals and incubated for a further 1 h at 4 °C. Diffraction data were collected at i04 at Diamond Light source as part of BAG allocation mx28172, autoprocessed using the autoPROC pipeline^54^ and phased using BUSTER^55^ using 4MEN. Manual model rebuilding was done using the CCP4 cloud^56^ alternated with structure refinement was performed in COOT^57^ and REFMAC5^58^. The resulting structure has been deposited in the PDB database (https://rcsb.org), with the accession code 8Q34. Refinement statistics are provided in Table 1, as well as in Supplementary Data 1.

**Table 1:** Refinement statistics for the X-ray structure 8Q34

PDB ID	8Q34^*^
**Data collection**	
Space group	P1
Cell dimensions	
*a*, *b*, *c* (Å)	37.24 44.09 78.48
*α*, *β*, *γ* (°)	90.03 90.00 90.03
Resolution (Å)	78.48–1.48 (1.51–1.48)
*R*_merge_	0.14 (1.31)
*I* / σ*I*	5.7 (0.8)
Completeness (%)	90.9 (51.3)
Redundancy	3.4 (3.0)
**Refinement**	
Resolution (Å)	1.48
No. reflections	259312
*R*_work_/*R*_free_	0.184/0.216
No. atoms	9429
Protein	8457
Ligand	168
Water	804
*B*-factors	
Protein	16
Ligand	14
Water	26.41
R.m.s. deviations	
Bond lengths (Å)	0.0068
Bond angles (°)	2.905

^*^Diffraction data from single crystal was used to determine the structure. Values in parentheses are for highest-resolution shell. Section headings are highlighted in bold.

### Binding site identification of KRas^G12D^ hits by LC-MS/MS peptide mapping

Modification sites were determined by proteolysis and reversed-phase LC-MS peptide mapping. Briefly, proteins were enzymatically digested in 25 mM NH_4_HCO_3_ solution after buffer exchange using Amicon Ultra-0.5 mL Centrifugal Filter units (10 kDa, Merck Millipore). Trypsin-LysC mixture and ProAlanase (Promega Corporation, Madison, USA) were used for the enzymatic digestion. Protein samples were reduced by dithiothreitol at 37 °C for 30 min. After reduction, proteins were digested using 1:50 enzyme:protein ratio or 4 h at 37 °C, followed by an additional short incubation with dithiothreitol (5 min, 37 °C). Overnight digestion was performed by Trypsin-LysC mixture in 50 mM NH_4_HCO_3_ solution at 37 °C. Tryptic digestion was stopped by adding formic acid in a final concentration of 0.2% (V/V). ProAlanase digestion was performed for 4 h at 37 °C in 50 mM HCl and was stopped by heating at 90 °C for 10 min. After digestions an additional short incubation with dithiothreitol was repeated (5 min, 37 °C).

Mass spectrometric experiments were performed on a high-resolution hybrid quadrupole-time-of-flight mass spectrometer equipped with a cyclic ion mobility separator (Waters Select Series Cyclic IMS, Waters Corp., Wilmslow, U.K.). Chromatographic separation was performed using a Waters Acquity I-Class UPLC system, coupled directly to the mass spectrometer. Waters Acquity CSH Peptide C18 UPLC column (2.1 × 150 mm, 1.7 µm) was used for chromatography. Gradient elution was performed under the following parameters: eluent A: 0.1% formic acid in water, eluent B: 0.1% formic acid in acetonitrile; flow rate: 300 µL/min; column temperature: 60 °C; gradient: 2 min: 2%B, 80 min: 45%B, 81 min: 85%B. HDMS^E^ experiments were performed using a single-pass cyclic ion mobility separation and fragmentation in the transfer cell with collision voltage ramping. MS data acquisition was performed with the following parameters: *m/z* 50–2000, V-mode, scan time: 0.3 s, single Lock Mass: leucine enkephalin; low energy: 6 V, high energy: ramping 19-45 V. BiopharmaLynx 1.3.5 software (Waters Corp., Wilmslow, U.K.) was used to for data analysis. Spectra of the hits are reported in Supplementary Note 3.

### Binding site identification of KRas^G12D^ hits by NMR spectroscopy

NMR samples contained 70 μM ^15^N-labeled GDP-bound KRas^G12D^ protein, 100–600 μM binding partner, 10 mM MgCl_2_, 3 mM NaN_3_, in PBS buffer, 5% DMSO-d6, 7% D_2_O, 0.5% DSS and the pH was set to 7.4. ^1^H,^15^N-SOFAST-HMQC (fast version of ^1^H,^15^N-HSQC) NMR spectra were acquired at 298 K on a Bruker AVANCE III spectrometer (Bruker Biospin, Rheinstetten, Germany) operating at 700.05 MHz for ^1^H and 70.94 MHz for ^15^N, equipped with a 5 mm Prodigy TCI H&F-C/N-D, z-gradient probe head. Temperature was calibrated by standard methanol solution. The chemical shifts were referenced with respect to the ^1^H-resonance of an internal DSS standard, while ^15^N chemical shifts were referenced indirectly via the gyromagnetic ratios according to the IUPAC conventions. All NMR data were processed with Bruker TOPSPIN 3.6 and analyzed in POKY software^59^. The shifted crosspeaks were compared to the free KRas^G12D^ chemical shifts^60^. Spectra of the hits are reported in Supplementary Note 4.

### Kras-SOS exchange assay

MANT-GDP loading assay: Kras^G12D^ protein was first buffer exchanged into low magnesium buffer (20 mM HEPES-NaOH (pH 7.5), 50 mM NaCl, 0.5 mM MgCl_2_) using a NAP5 column (catalog no.: 17-0583-1, Cytiva). The proteins were then incubated with 20-fold molar excess of *N*-methylanthraniloyl (MANT)-GDP (catalog no.: 69244, Sigma-Aldrich) in loading buffer (50 mM NaCl, 20 mM HEPES-NaOH [pH 7.5], 0.5 mM MgCl_2_, 10 mM EDTA, and 1 mM Dithiothreitol (DTT)) in a total volume of 200 μL at 20 °C for 90 min. The reaction was stopped by adding MgCl_2_ to a final concentration of 10 mM, then incubated at 20 °C for 30 min. The unbound MANT-GDP was removed using the NAP-5 column equilibrated with nucleotide exchange buffer (40 mM HEPES-NaOH (pH 7.5), 50 mM NaCl, 10 mM MgCl_2_, 2 mM DTT).

MANT-GDP exchange assay: First the MANT-GDP bound Kras G12D protein (in a final 1 μM molar concentration) was preincubated with the inhibitor molecules for 60 min. After the preincubation the samples were exposed to UV light for 10 min with a wavelength of 366 nm. The UV-treated MANT-GDP Kras-inhibitor mix was loaded into a black 384 well microplate. The nucleotide exchange reaction was initiated by adding 100 fold molar excess of GppNHp (catalog no.: G0635, Sigma-Aldrich), a non-hydrolyzable GTP analog and the SOS1 exchange domain (catalog no.: GE02, Cytoskeleton, Inc.) protein in 0.5 μM final concentration. The change in fluorescence intensity was measured every 30 s in room temperature for 60 min on an EnSpire plate reader (PerkinElmer, Inc.). The measured fluorescence values were fitted to a single exponential function by using GraphPad Prism 8 software.

### STAT5B-NTD expression, purification, and MST measurements

Recombinant protein expression was performed similarly to our recently published protocol for expressing full-length STAT5B^61^. STAT5B *N*-terminal domain (1-123, NCBI Accession Number NP_036580.2) was codon optimized and synthesized by Genscript and cloned into pET28b+ plasmid using NheI and XhoI cloning sites with an *N*-terminal His-SUMO tag. The plasmid was used to transform BL21 RILP cells and single colonies were selected and used to inoculate 3 mL cultures in Super Broth (with 34 µg/mL chloramphenicol and 50 µg/mL kanamycin). Once cultures reached an OD_600_ of ~1.0, they were transferred to 1 L Super Broth (supplemented with 10 mM MgSO_4_, 0.1% [v/v] glucose, 34 µg/mL chloramphenicol and 50 µg/mL kanamycin). At OD_600_ = 1.5, the temperature was reduced to 16 °C and the media was supplemented with 1 mM IPTG and 3% (v/v) ethanol. The cells were harvested following 20 h induction and frozen at −80 °C.

For protein purification, the cell pellets were thawed in lysis buffer and ruptured through sonication. The cell lysate was cleared by centrifugation and loaded onto a 3 mL Ni^2+^-NTA resin (GE Healthcare). The column was washed and the protein was eluted and loaded onto a Superdex 200 Increase 10/300 GL column (GE Healthcare). The fractions were treated with His-Ulp1 protease to cleave the His-SUMO tag and passed through a 1 mL Ni^2+^-NTA resin to remove any residual tag. The flow through was collected and assessed for purity via SDS-PAGE and the protein was dialyzed into 100 mM HEPES pH 7.4, 2% (v/v) glycerol. Protein concentration was determined by BCA assay (Thermo Fisher Scientific) and aliquots of *N*-terminal domain were flash-frozen in liquid nitrogen and stored at −80 °C. The compositions of all purification buffers are listed in additional detail in ref. ^61^.

For the microscale thermophoresis (MST) studies, we prepared 16 two-fold serial dilutions of compounds starting from 500 μM. Titration series were prepared that contained 10 μL of compounds’ solutions of varying concentrations and 10 μL RED-NHS 2nd Generation labeled STAT5B NTD with a concentration of 168 nM for compound **PhP097**, and a concentration of 84 nM for compound **PhP065**. Final buffer composition included 1X PBS containing 0.5% DMSO. All measurements were taken in Premium Coated Capillaries on a Monolith NT.115 instrument (NanoTemper Technologies, Munich, Germany) using 80% infrared laser power for compound **PhP097**, 60% infrared laser power for compound **PhP065**, and an LED excitation source with *λ* = 650 nm at a temperature of 25 °C. Results were expressed as the mean of two separate experiments, with three technical replicates each. GraphPad Prism 9.5.1 software was used to fit the data and to determine the *K*_D_ values.

MV4-11 and MOLM-13 cell lines were purchased from DSMZ (Braunschweig, Germany), and grown at 37 °C and 5% CO_2_ in RPMI 1640 medium (Gibco^TM^, Thermo Fisher Scientific). Media were supplemented with 10% fetal calf serum (FCS), 10 U/mL penicillin, 10 µg/mL streptomycin and 2 mM L-glutamine (all Gibco^TM^, Thermo Fisher Scientific*)*.

To determine the IC_50_ of the selected compounds on the cell lines, CellTiter-Blue^®^ cell viability assay (Promega) was performed. For this, cells were seeded in 96-well flat bottom plates at a cell density of 10,000 cells/well. Cells were treated in triplicates with the compound of interest at various concentrations or with 10 μM Bortezomib (S1013; Selleck Chemicals, Houston, TX, USA) as a positive control. Cell viability of treated cell lines was measured using CellTiter-Blue^®^ after 72 h incubation. Plates were measured using a GloMax^®^ plate reader (Promega) and IC_50_ values were determined by non-linear regression using the GraphPad Prism 9.1.1 (GraphPad Software, Inc.) and the data are reported as mean values ± SEM.

### Reporting summary

Further information on research design is available in the Nature Portfolio Reporting Summary linked to this article.

### Supplementary information

Supplementary information

Description of Additional Supplementary Files

Supplementary data 1

Supplementary data 2

Source Data 1

Reporting Summary

## Data Availability

Structure data that support the findings of this study have been deposited in the PDB database (https://rcsb.org), with the accession code 8Q34. Data generated during the computational and experimental screening of the described fragment library are reported in Supplementary Data 1. BRD4-BD1 ligands and bioactivity data were downloaded from the ChEMBL database (https://www.ebi.ac.uk/chembl/). Source data are provided with this paper.
