# Supplementary material for: Mapping protein binding sites by photoreactive fragment pharmacophores
Source: Commun Chem. 2024 Jul 31;7:168. doi: 10.1038/s42004-024-01252-w (PMC11292009; doi:10.1038/s42004-024-01252-w)
Supplement: Supplementary file 1 — Supplementary information [file 42004_2024_1252_MOESM1_ESM.pdf]

Supplementary information

Péter Ábrányi-Balogh[1,2,3§], Dávid Bajusz[1,2§], Zoltán Orgován[1,2§], Aaron B. Keeley[1], László Petri[1,2], Nikolett Péczka[1,2,3], Tibor Viktor Szalai[1,2,4], Gyula Pálfy[5], Márton Gadanecz[2,5], Emma K. Grant[6], Tímea Imre[2,7], Tamás Takács[2,8,9], Ivan Ranđelović[10,11], Marcell Baranyi[11,12], András Marton[13,14], Gitta Schlosser[15], Qirat F. Ashraf[16], Elvin de Araujo[17], Tamás Karancsi[13,14], László Buday,[2,8] József Tóvári[10], András Perczel[2,5], Jacob Bush[6], György M. Keserű*[1,2,3]

See full list of affiliations in the article.

§These authors contributed equally to this work.

*Corresponding author: György M. Keserű, email: keseru.gyorgy@ttk.hu

Table of contents

1. Supplementary Table 1. Number of PhP fragment hits against different protein targets. 2

2. Supplementary Table 2. Number of proteins labelled by the proteomics probes of ref. 50 most similar to PhP fragment hits. 3

3. Supplementary Figure 1. Structure of STAT5B-NTD *vs*. range of Dexter-energy transfer. . 4

4. Supplementary Figure 2. MS spectrum of the photocatalyst-labeled STAT5B N-terminal domain. 4

5. Supplementary Note 1. Compound characterization........ 5

6. Supplementary Note 2. Intact MS protocols and results........ 23

   6.1 Screening against BRD4-BD1 and KRas^{G12D}........ 23

   6.2 Screening against STAT5B-NTD........ 24

   6.3 Intact MS spectra of hit fragments vs. BRD4-BD1........ 25

   6.4 Intact MS spectra of hit fragments vs. KRas^{G12D}........ 25

   6.5 Intact MS spectra of hit fragments vs. STAT5B-NTD........ 27

7. Supplementary Note 3. Binding site identification by LC-MS/MS peptide mapping 28

   7.1 Sample preparation and data acquisition for BRD4-BD1 28

   7.2 Sample preparation and data acquisition for STAT5B-NTD 29

   7.3 MS spectra of digested BRD4-BD1 after labeling by hit fragments........ 30

   7.4 MS spectra of digested KRas^{G12D} after labeling by hit fragments........ 32

   7.5 MS spectra of digested STAT5B-NTD after labeling by hit fragments........ 34

8. Supplementary Note 4. HSQC NMR spectra of fragment hits against KRas^{G12D}........ 36

9. Supplementary References........ 37

1. Supplementary Table 1. Number of PhP fragment hits against different protein targets.

Labeling efficiency	CA	BRD4	KRas G12D	Lyo	Myo	STAT5B	STAT5B + Photocat
>10%	4	7	11	3	3	2	6
>5%	1	2	2	2	0	2	18
1-5%	12	21	12	18	13	22	36
<1%	83	64	74	77	84	74	40
not available		6	1				

Among the detected PhP fragment hits, CA was mostly preferred by PhP003 (4.8%) that moderately labelled the other targets (>1.1% for each). BRD4-BD1 was mostly preferred by PhP053 (6.7% vs. >2%). In the case of KRas, PhP048 and PhP012 were the most selective compounds (29.4% and 15.8% vs. >3%, respectively). PhP092, PhP001 and PhP088 preferred Lyo (35.6%, 19.2% and 4.8% vs. >1%), while Myo was targeted selectively only by PhP082 (52.9% vs. >0.5%). STAT5B-NTD was most selectively labelled by PhP040, PhP077, PhP065 and PhP097 (75.0%, 20.0%, 6.5% and 5.7% vs. >3%, respectively). Note that a higher labeling efficiency does not necessarily translate to a stronger affinity in the secondary binding assays that are carried out without irradiation.

2. Supplementary Table 2. Number of proteins labelled by the proteomics probes of ref. 50 most similar to PhP fragment hits.

PhP probe ID	PhP probe structure	Proteomics probe ID	Proteomics probe structure	Tanimoto similarity	No. of labeled proteins
PhP065	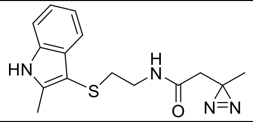	C169	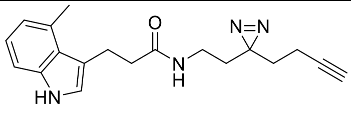	0.289	308
PhP097	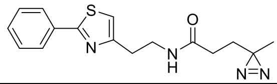	C071	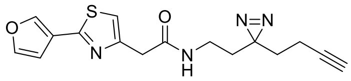	0.373	100
PhP072	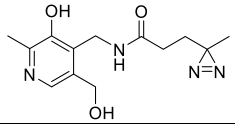	C415	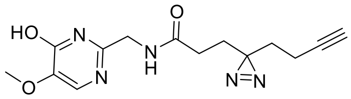	0.175	NA
PhP060	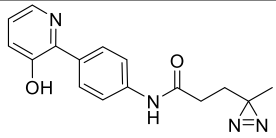	C264	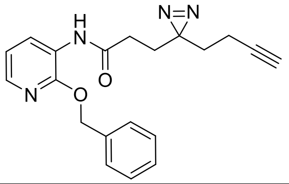	0.406	181
PhP071	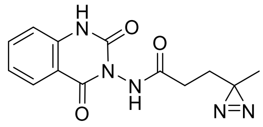	C417	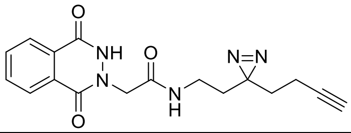	0.373	10
PhP053	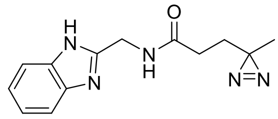	C420	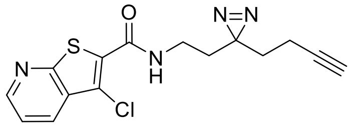	0.259	228

Tanimoto similarities were calculated using Morgan fingerprints (radius=2) of the probes.

3. Supplementary Figure 1. Structure of STAT5B-NTD *vs*. range of Dexter-energy transfer.

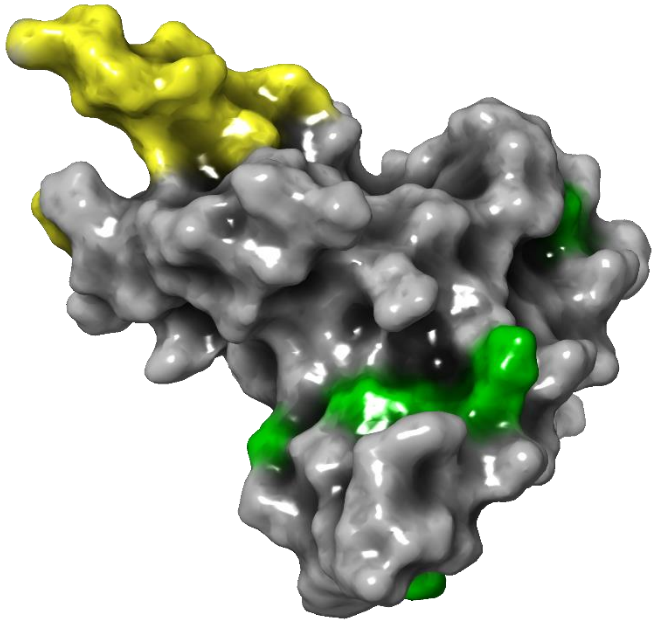

STAT5B-NTD structure, with residue coloring based on whether the photocatalyst can reach that area with Dexter-energy transfer. Green: all possible lysine residues, where the photocatalyst Ir-G2-PEG3-COOH can attach. Grey: the photocatalyst can activate fragments in the area. Yellow: the photocatalyst cannot activate fragments that bind in the area. (All calculations are based on the following information: (i) the photocatalyst linker has a length of approximately 15 Å, and (ii) the Dexter-energy transfer has a range of 10 Å.[2])

4. Supplementary Figure 2. MS spectrum of the photocatalyst-labeled STAT5B N-terminal domain.

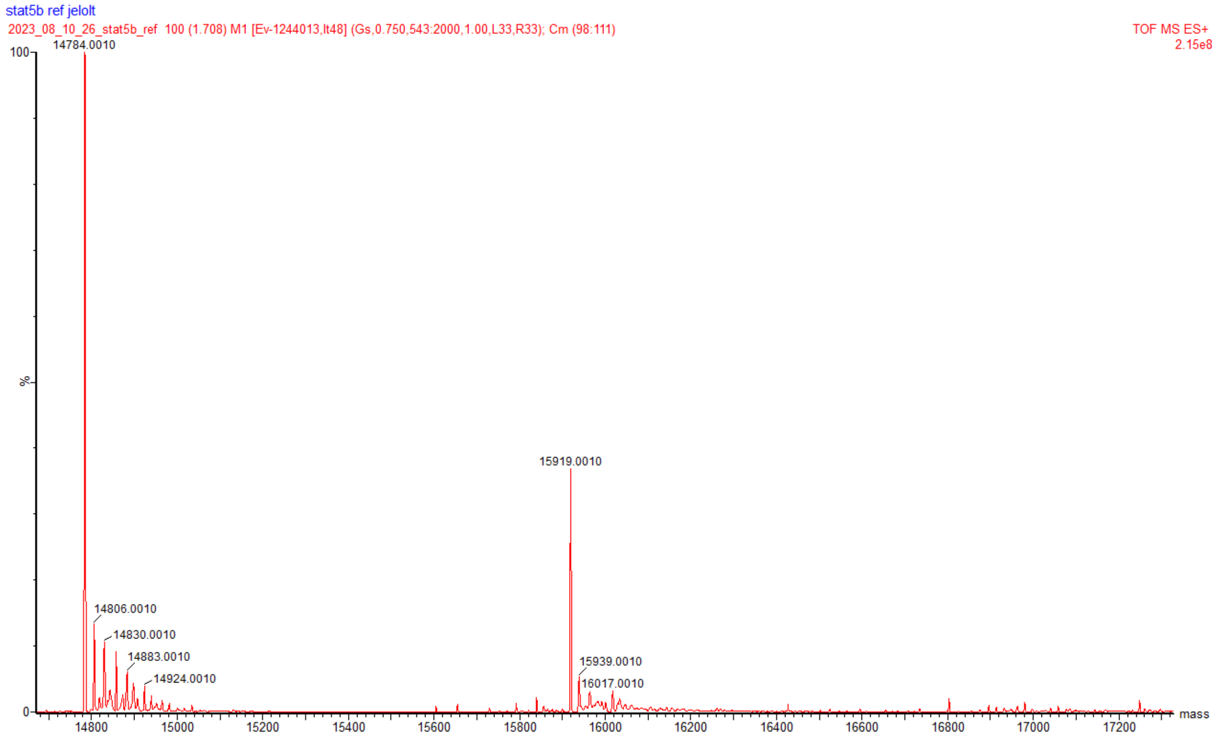

5. Supplementary Note 1. Compound characterization

The PhP library was synthesized following the general procedures available in the Methods section, based on our previous work.[1] Compounds PhP006, PhP015, PhP051, PhP056, PhP057 and PhP099 were provided, courtesy of GSK.

Structures of the compounds are provided as SMILES strings and figures in Supplementary Data 1, as the "core fragment" (fragment without the amine handle, included in pharmacophore screening during library selection), "amine" (fragment with the amine handle), and "PhP" (fragment with the diazirine-type photoaffinity tag attached to the amine handle). ^{1}H NMR assignments and further analytical properties are listed here, while ^{1}H NMR spectra are collated in Supplementary Data 2.

PhP001: Ethyl-6-benzyl-2-(3-(3-methyl-3H-diazirin-3-yl)propanamido)-4,5,6,7-tetrahydrothieno[2,3-c]pyridine-3-carboxylate (0.014 mmol, 6 mg, 9%) as an off-white solid. ^{1}H NMR (300 MHz, $CDCl_3$) δ 11.26 (s, 1H), 7.46 – 7.31 (m, 5H), 4.42 – 4.25 (m, 2H), 3.68 (d, *J* = 40.8 Hz, 4H), 2.86 (d, *J* = 21.6 Hz, 4H), 2.32 (t, *J* = 7.5 Hz, 2H), 1.84 (t, *J* = 7.9 Hz, 2H), 1.40 – 1.33 (m, 3H), 1.05 (s, 3H). LC-MS: t_R = 0.77 min, 100% by UV, HRMS m/z: $[M + H]^+$ calcd for $C_{22}H_{27}N_4O_3S$, 427.1803; found, 427.1811.

PhP002: *N*-(1-(3,4-dihydroxyphenyl)propan-2-yl)-3-(3-methyl-3H-diazirin-3-yl)propanamide (0.141 mmol, 39 mg, 94%) as an off-white solid. ^{1}H NMR (500 MHz, DMSO-d_6) δ 8.65 (d, *J* = 45.2 Hz, 2H), 7.73 (d, *J* = 8.1 Hz, 1H), 6.64 – 6.53 (m, 2H), 6.45 – 6.37 (m, 1H), 3.85 (dd, *J* = 14.4, 7.2 Hz, 1H), 2.40 – 2.30 (m, 1H), 1.93 (dd, *J* = 10.6, 5.2 Hz, 2H), 1.53 (dd, *J* = 10.7, 5.0 Hz, 2H), 0.97 (s, 6H).

LC-MS: t_R = 0.73 min, 97% by UV, HRMS m/z: $[M + H]^+$ calcd for $C_{14}H_{20}N_3O_3$, 278.1504; found, 278.1505.

PhP003: *N*-(2-(1-methyl-1H-imidazol-2-yl)ethyl)-3-(3-methyl-3H-diazirin-3-yl)propanamide (0.049 mmol, 11.5 mg, 33%) as a yellow solid. ^{1}H NMR (300 MHz, DMSO-d_6) δ 8.09 (s, 1H), 7.57 (dd, *J* = 8.8, 2.4 Hz, 2H), 3.77 (s, 3H), 3.41 (s, 2H), 3.02 (d, *J* = 18.2 Hz, 2H), 1.97 – 1.86 (m, 2H), 1.59 – 1.46 (m, 2H), 0.96 (s, 3H).

LC-MS: t_R = 1.45 min, 90% by UV, HRMS m/z: $[M + H]^+$ calcd for $C_{11}H_{18}N_5O$, 236.1511; found, 236.1512.

PhP004: *N*-((2,3-dimethyl-1H-indol-5-yl)methyl)-3-(3-methyl-3H-diazirin-3-yl)propanamide (0.134 mmol, 38.3 mg, 89%) as an off-white solid. ^{1}H NMR (300 MHz, $CDCl_3$) δ 7.36 (s, 1H),

7.20 (d, *J* = 8.2 Hz, 1H), 7.01 (d, *J* = 8.2 Hz, 1H), 5.67 (s, 1H), 2.36 (s, 3H), 2.20 (d, *J* = 1.9 Hz, 2H), 2.17 (s, 3H), 2.03 – 1.96 (m, 2H), 1.81 – 1.74 (m, 2H), 1.02 (s, 3H).

LC-MS: t_R = 1.01 min, 100% by UV, HRMS m/z: $[M + H]^+$ calcd for $C_{16}H_{21}N_4O$, 285.1715; found, 285.1718.

PhP005: ethyl (R)-2-(3-(3-methyl-3H-diazirin-3-yl)propanamido)-2-phenylacetate (0.135 mmol, 39 mg, 90%) as an off-white solid. ^{1}H NMR (300 MHz, $CDCl_3$) δ 7.39 – 7.31 (m, 5H), 6.48 (s, 1H), 5.55 (d, *J* = 7.3 Hz, 1H), 4.31 – 4.08 (m, 2H), 2.08 (q, *J* = 7.0 Hz, 2H), 1.79 – 1.68 (m, 2H), 1.22 (q, *J* = 6.7 Hz, 3H), 1.00 (s, 3H).

LC-MS: t_R = 1.03 min, 97% by UV, HRMS m/z: $[M + H]^+$ calcd for $C_{15}H_{20}NO_3$, 262.1443; found, 262.1440.

PhP006: N-(2-(4-(4-methoxyphenyl)thiazol-2-yl)ethyl)-3-(3-methyl-3H-diazirin-3-yl)propanamide (0.147 mmol, 43 mg, 85%) as an off-white solid. ^{1}H NMR (500 MHz, $CDCl_3$) δ 7.87 – 7.74 (m, 2H), 7.25 – 7.18 (m, 1H), 7.02 – 6.91 (m, 2H), 6.44 (s, 1H), 3.91 – 3.82 (m, 3H), 3.74 (q, *J* = 6.3 Hz, 2H), 3.22 (q, *J* = 6.5 Hz, 2H), 2.02 (q, *J* = 7.6 Hz, 2H), 1.76 (q, *J* = 8.2 Hz, 2H), 1.01 (s, 3H). LC-MS: t_R = 1.45 min, 93% by UV, HRMS m/z: $[M + H]^+$ calcd for $C_{17}H_{21}N_4O_2S$, 345.1385; found, 345.1383

PhP007: *N*-(4-methyl-3-oxo-3,4-dihydro-2H-benzo[b][1,4]oxazin-6-yl)-3-(3-methyl-3H-diazirin-3-yl)propanamide (0.127 mmol, 36.7 mg, 85%) as an off-white solid. 1H NMR (300 MHz, $CDCl_3$) δ 7.54 (s, 1H), 7.45 (s, 1H), 6.93 – 6.76 (m, 2H), 4.57 (s, 2H), 3.32 (s, 3H), 2.23 – 2.06 (m, 2H), 1.92 – 1.77 (m, 2H), 1.06 (s, 3H). LC-MS: t_R = 0.84 min, 100% by UV, HRMS m/z: $[M + H]^+$ calcd for $C_{14}H_{17}N_4O_3$, 289.13; found, 289.1305.

PhP008: *N*-(isoquinolin-4-ylmethyl)-3-(3-methyl-3H-diazirin-3-yl) propanamide (0.150 mmol, 40.2 mg, 100%) as a yellow solid. ^{1}H NMR (600 MHz, DMSO-d_6) δ ppm 0.94 - 0.99 (s, 3 H), 1.61 (br t, *J* = 7.64 Hz, 2 H), 2.04 (br t, *J* = 7.64 Hz, 2 H), 4.69 - 4.74 (m, 2 H), 7.71 - 7.76 (m, 1 H), 7.85 (m, 1 H), 8.03 - 8.15 (m, 1 H), 8.18 (br d, *J* = 8.07 Hz, 1 H), 8.41 - 8.49 (s, 1 H), 9.25 - 9.30 (s, 1 H); LC-MS: t_R = 0.46 min, 86% by UV, HRMS m/z: $[M + H]^+$ calcd for $C_{15}H_{16}N_4O$, 268.1324; found, 268.1321.

PhP009: *N*-(4-(4-(hydroxymethyl)piperidin-1-yl)benzyl)-3-(3-methyl-3H-diazirin-3-yl)propanamide (0.015 mmol, 4.9 mg, 10%) as an off-white solid. ^{1}H NMR (300 MHz, DMSO-d_6) δ 8.41 (d, *J* = 6.3 Hz, 1H), 7.52 (s, 2H), 7.38 (d, *J* = 7.8 Hz, 2H), 4.27 (d, *J* = 5.6 Hz, 2H),

3.60 (d, *J* = 11.6 Hz, 4H), 3.35 (d, *J* = 5.4 Hz, 3H), 2.04 (t, *J* = 7.8 Hz, 2H), 1.92 (d, *J* = 13.7 Hz, 2H), 1.75 (s, 1H), 1.60 (q, *J* = 9.7 Hz, 4H), 0.98 (s, 3H).

LC-MS: t_R = 0.42 min, 84.7% by UV, HRMS m/z: $[M + H]^+$ calcd for $C_{18}H_{27}N_4O_2$, 331.2134; found, 331.2134.

PhP010: *N*-((5-methyl-1H-imidazol-4-yl)methyl)-3-(3-methyl-3H-diazirin-3-yl)propanamide (0.131 mmol, 29 mg, 87%) as an off-white solid. ^{1}H NMR (300 MHz, DMSO-d_6) δ 13.99 (s, 1H), 8.85 (d, *J* = 2.2 Hz, 1H), 8.37 (d, *J* = 5.7 Hz, 1H), 4.23 (d, *J* = 5.3 Hz, 2H), 2.24 (s, 3H), 2.00 (t, *J* = 7.7 Hz, 2H), 1.58 (t, *J* = 7.7 Hz, 2H), 0.96 (s, 3H).

LC-MS: t_R = 1.19 min, 85% by UV, HRMS m/z: $[M + H]^+$ calcd for $C_{10}H_{16}N_5O$, 222.1354; found, 222.1353.

PhP011: 4-(2-(3-(3-methyl-3H-diazirin-3-yl)propanamido)thiazol-4-yl)-1H-pyrrole-2-carboxamide (0.137 mmol, 43.7 mg, 91%) as an off-white solid. ^{1}H NMR (300 MHz, DMSO-d_6) δ 12.12 (s, 1H), 11.50 (s, 1H), 7.52 (s, 1H), 7.23 – 6.91 (m, 4H), 2.38 – 2.31 (m, 2H), 1.70 (s, 2H), 1.02 (s, 3H).LC-MS: t_R = 0.79 min, **91**% by **UV**, HRMS m/z: $[M + H]^+$ calcd for $C_{13}H_{15}N_6O_2S$, 319.0977; found, 319.0980.

PhP012: *N*-(1-(4-fluorophenyl)piperidin-4-yl)-3-(3-methyl-3H-diazirin-3-yl)propanamide (0.027 mmol, 8.3 mg, 18%) as an yellow solid. ^{1}H NMR (300 MHz, $CDCl_3$) δ 6.98 – 6.78 (m, 4H), 5.41 (s, 1H), 3.91 (s, 1H), 3.48 (d, *J* = 11.8 Hz, 2H), 2.81 (t, *J* = 11.9 Hz, 2H), 2.09 – 1.93 (m, 4H), 1.83 – 1.70 (m, 2H), 1.57 (q, *J* = 12.3 Hz, 2H), 1.04 (s, 3H). LC-MS: t_R = 0.7 min, 90% by UV, HRMS m/z: $[M + H]^+$ calcd for $C_{16}H_{22}N_4OF$, 305.1777; found, 305.1783.

PhP013: *N*-(1-(3-(benzyloxy)phenyl)propan-2-yl)-3-(3-methyl-3H-diazirin-3-yl)propanamide (0.049 mmol, 17.2 mg, 33%) as an off-white solid. ^{1}H NMR (300 MHz, $CDCl_3$) δ 7.47 – 7.31 (m, 5H), 7.24 – 7.15 (m, 1H), 6.87 – 6.74 (m, 3H), 5.24 (d, *J* = 8.2 Hz, 1H), 5.06 (d, *J* = 1.8 Hz, 2H), 4.25 (p, *J* = 7.0 Hz, 1H), 2.86 – 2.61 (m, 2H), 1.96 – 1.83 (m, 2H), 1.80 – 1.65 (m, 2H), 1.11 (dd, *J* = 6.6, 1.9 Hz, 3H), 1.00 (s, 3H).

LC-MS: t_R = 1.25 min, 87% by UV, HRMS m/z: $[M + H]^+$ calcd for $C_{21}H_{26}N_3O_2$, 352.2025; found, 352.2026.

PhP014: (3-(3-methyl-3H-diazirin-3-yl)propanoyl)tryptophanate (0.037 mmol, 12 mg, 25%) as an off-white solid. ^{1}H NMR (300 MHz, $CDCl_3$) δ 8.20 (s, 1H), 7.51 (d, *J* = 7.7 Hz, 1H), 7.35 (d, *J* = 7.6 Hz, 1H), 7.23 – 7.05 (m, 2H), 7.00 (d, *J* = 6.3 Hz, 1H), 5.96 (s, 1H), 4.95 (s, 1H), 3.75 – 3.62 (m, 3H), 3.32 (s, 2H), 1.96 (dt, *J* = 13.0, 5.6 Hz, 2H), 1.70 (d, *J* = 8.2 Hz, 2H), 0.99 (s, 3H).

LC-MS: t_R = 0.98 min, 100% by UV, HRMS m/z: $[M + H]^+$ calcd for $C_{17}H_{21}N_4O_3$, 329.1613; found, 329.1611

PhP015: *tert*-butyl (3-(3-(3-methyl-3H-diazirin-3-yl)propanamido)cyclohexyl)carbamate (0,085 mmol, 25 mg, 91%) as an off-white solid. 1H NMR (300 MHz, $CDCl_3$) δ 5.47 (s, 1H), 4.59 (s, 1H), 4.08 (s, 1H), 3.76 (s, 1H), 2.05 – 1.63 (m, 10H), 1.43 (d, J = 5.4 Hz, 11H), 1.02 (s, 3H). LC-MS: t_R = 1.17 min, 94% by UV, HRMS m/z: $[M + H]^+$ calcd for $C_{16}H_{29}N_4O_3$, 325.2239; found, 325.2243

PhP016: *N*-(2-(*tert*-butyl)-5,6,7,8-tetrahydroimidazo[1,2-a]pyridin-6-yl)-3-(3-methyl-3H-diazirin-3-yl)propanamide (0.069 mmol, 20.9 mg, 46%) as an off-white solid. ^{1}H NMR (300 MHz, DMSO-d_6) δ 8.18 (s, 1H), 7.25 (s, 1H), 4.36 – 4.04 (m, 2H), 3.82 (s, 1H), 3.02 (s, 2H), 2.11 – 1.93 (m, 4H), 1.58 (t, *J* = 8.2 Hz, 2H), 1.24 (s, 9H), 0.95 (s, 3H).

LC-MS: t_R = 0.49 min, 99% by UV, HRMS m/z: $[M + H]^+$ calcd for $C_{16}H_{26}N_5O$, 304.2137; found, 304.2144.

PhP017: *N*-(2-methyl-1-phenyl-1H-benzo[d]imidazol-5-yl)-3-(3-methyl-3H-diazirin-3-yl)propanamide (0.146 mmol, 48.7 mg, 97%) as an yellow solid. ^{1}H NMR (300 MHz, DMSO-d_6) δ 9.97 (s, 1H), 7.97 (s, 1H), 7.63 (d, *J* = 7.1 Hz, 2H), 7.53 (d, *J* = 6.5 Hz, 3H), 7.32 (d, *J* = 8.7 Hz, 1H), 7.04 (d, *J* = 7.5 Hz, 1H), 2.41 (d, *J* = 4.5 Hz, 3H), 2.23 (t, *J* = 7.2 Hz, 2H), 1.74 – 1.65 (m, 2H), 1.03 (s, 3H).

LC-MS: t_R = 0.65 min, 96% by UV, HRMS m/z: $[M + H]^+$ calcd for $C_{19}H_{20}N_5O$, 334.1667; found, 334.1671.

PhP018: *N*-(1-((4-methoxyphenyl)sulfonyl)piperidin-4-yl)-3-(3-methyl-3H-diazirin-3-yl)propanamide (0.025 mmol, 9.7 mg, 17%) as an off-white solid. ^{1}H NMR (300 MHz, $CDCl_3$) δ 7.86 – 7.63 (m, 2H), 7.08 – 6.93 (m, 2H), 5.65 – 5.43 (m, 1H), 3.88 (s, 3H), 3.73 (d, *J* = 11.9 Hz, 3H), 2.37 (t, *J* = 11.8 Hz, 2H), 1.96 (q, *J* = 7.2 Hz, 4H), 1.73 (d, *J* = 7.4 Hz, 2H), 1.62 – 1.51 (m, 2H), 0.99 (s, 3H). LC-MS: t_R = 0.97 min, 90% by UV, HRMS m/z: $[M + H]^+$ calcd for $C_{17}H_{25}N_4O_4S$, 381.1596; found, 381.1598.

PhP019: methyl 2-(3-(3-methyl-3H-diazirin-3-yl)propanamido)-2-(p-tolyl)acetate (0.135 mmol, 39 mg, 90%) as an off-white solid. ^{1}H NMR (300 MHz, $CDCl_3$) δ 7.33 – 7.23 (m, 2H), 7.20 – 7.13 (m, 2H), 6.40 (s, 1H), 5.52 (d, *J* = 7.2 Hz, 1H), 3.72 (s, 3H), 2.33 (s, 3H), 2.19 – 1.98 (m, 2H), 1.73 (t, *J* = 7.7 Hz, 2H), 0.99 (s, 3H). LC-MS: t_R = 1.04 min, 87% by UV, HRMS m/z: $[M + H]^+$ calcd for $C_{15}H_{20}N_3O_3$, 290.1504; found, 290.1503.

PhP020: *N*-(2-(1-methyl-1H-benzo[d]imidazol-2-yl)ethyl)-3-(3-methyl-3H-diazirin-3-yl)propanamide (0.145 mmol, 41.5 mg, 97%) as an off-white solid. ^{1}H NMR (300 MHz, $CDCl_3$) δ 7.73 (d, *J* = 7.2 Hz, 1H), 7.44 – 7.31 (m, 3H), 7.05 (s, 1H), 3.83 (d, *J* = 16.8 Hz, 5H), 3.20 (s, 2H), 2.03 (t, *J* = 7.5 Hz, 2H), 1.67 (d, *J* = 7.8 Hz, 2H), 0.94 (s, 3H). LC-MS: t_R = 0.44 min, 100% by UV, HRMS m/z: $[M + H]^+$ calcd for $C_{15}H_{20}N_5O$, 286.1667; found, 286.1674.

PhP021: *N*-((1,3-dimethyl-1H-pyrazol-4-yl)methyl)-3-(3-methyl-3H-diazirin-3-yl)propanamide (0.074 mmol, 17.5 mg, 49%) as an yellow solid. ^{1}H NMR (300 MHz, $CDCl_3$) δ 5.46 (s, 1H), 4.22 (s, 2H), 3.79 (s, 3H), 2.21 (s, 3H), 1.97 (d, *J* = 10.0 Hz, 2H), 1.78 (d, *J* = 9.6 Hz, 2H), 1.02 (s, 3H).

LC-MS: t_R = 0.61 min, 98% by UV, HRMS m/z: $[M + H]^+$ calcd for $C_{11}H_{18}N_5O$, 236.1511; found, 236.1513.

PhP022: 3-(3-methyl-3H-diazirin-3-yl)-N-(4-morpholinobutyl)propanamide (0.077 mmol, 21 mg, 51%) as an off-white solid. ^{1}H NMR (300 MHz, DMSO-d_6) δ 7.87 (s, 1H), 3.94 (s, 3H), 3.63 (s, 3H), 3.12 – 3.01 (m, 6H), 1.93 (d, *J* = 6.6 Hz, 2H), 1.66 – 1.50 (m, 5H), 1.41 (s, 2H), 0.97 (s, 3H).

LC-MS: t_R = 0.41 min, 91% by UV, HRMS m/z: $[M + H]^+$ calcd for $C_{13}H_{25}N_4O_2$, 269.1977; found, 269.1980.

PhP023: *N*-((7-methyl-1H-benzo[d]imidazol-2-yl)methyl)-3-(3-methyl-3H-diazirin-3-yl)propanamide (0.147 mmol, 40 mg, 98%) as an off-white solid. ^{1}H NMR (300 MHz, $CDCl_3$) δ 7.74 (s, 1H), 7.45 (d, *J* = 8.3 Hz, 1H), 7.36 (s, 1H), 7.18 (d, *J* = 8.2 Hz, 1H), 4.73 (d, *J* = 5.7 Hz, 2H), 2.45 (s, 3H), 2.13 (t, *J* = 7.7 Hz, 2H), 1.66 (t, *J* = 7.8 Hz, 2H), 1.01 – 0.90 (m, 3H). LC-MS: t_R = 0.49 min, 98% by UV, HRMS m/z: $[M + H]^+$ calcd for $C_{14}H_{18}N_5O$, 272.1511; found, 272.1515.

PhP024: tert-butyl 3-((3-(3-methyl-3H-diazirin-3-yl)propanamido)methyl)benzoate (0.040 mmol, 12.6 mg, 27%) as an off-white solid. ^{1}H NMR (300 MHz, $CDCl_3$) δ 7.91 – 7.81 (m, 2H), 7.49 – 7.31 (m, 2H), 5.89 (s, 1H), 4.46 (d, *J* = 5.9 Hz, 2H), 2.10 – 1.94 (m, 2H), 1.84 – 1.72 (m, 2H), 1.58 (s, 9H), 1.02 (s, 3H).

LC-MS: t_R = 1.15 min, 86% by UV, HRMS m/z: $[M + H]^+$ calcd for $C_{17}H_{24}N_3O_3$, 318.1817; found, 318.1819.

PhP025: 3-(3-methyl-3H-diazirin-3-yl)-N-(2-(methylsulfonamido)benzyl)propanamide (0.070 mmol, 19.4 mg, 47%) as an off-white solid. ^{1}H NMR (500 MHz, $CDCl_3$) δ 9.12 (s, 1H), 7.58 – 7.51 (m, 1H), 7.33 (dd, J = 7.5, 1.6 Hz, 1H), 7.19 – 7.11 (m, 1H), 6.22 (s, 1H), 4.40 (d, J = 6.6 Hz, 2H), 3.07 (s, 3H), 1.99 (dd, J = 8.6, 6.7 Hz, 2H), 1.77 (dd, J = 8.5, 6.8 Hz, 2H),

0.98 (s, 3H). LC-MS: t_R = 0.53 min, **93**% by **UV**, HRMS m/z: [M + H]$^+$ calcd for $C_{13}H_{19}N_4O_3S$, 311.1099.1569; found, 311.1180.

PhP026: 3-(3-methyl-3H-diazirin-3-yl)-N-(pyridin-2-yl(p-tolyl)methyl)propanamide (0.040 mmol, 12.2 mg, 27%) as an off-white solid. ^{1}H NMR (300 MHz, $CDCl_3$) δ 8.56 (s, 1H), 7.66 – 7.54 (m, 2H), 7.20 (d, *J* = 7.9 Hz, 4H), 7.08 (d, *J* = 7.8 Hz, 2H), 6.09 (d, *J* = 7.0 Hz, 1H), 2.28 (s, 3H), 2.14 (t, *J* = 8.3 Hz, 2H), 1.74 (t, *J* = 8.1 Hz, 2H), 0.98 (s, 3H).
LC-MS: t_R = 0.94 min, 100% by UV, HRMS m/z: [M + H]$^+$ calcd for $C_{18}H_{21}N_4O$, 309.1715; found, 309.1724.

PhP027: 4-chloro-N-(2-(3-(3-methyl-3H-diazirin-3-yl)propanamido)ethyl)benzamide (0.142 mmol, 43.7 mg, 95%) as an off-white solid. ^{1}H NMR (300 MHz, $CDCl_3$) δ 7.82 – 7.72 (m, 2H), 7.48 – 7.36 (m, 3H), 6.42 (s, 1H), 3.64 – 3.45 (m, 4H), 2.05 – 1.95 (m, 2H), 1.79 – 1.73 (m, 2H), 0.98 (s, 3H). LC-MS: t_R = 0.89 min, 98% by UV, HRMS m/z: [M + H]$^+$ calcd for $C_{14}H_{18}N_4O_2Cl$, 309.1118; found, 309.1118.

PhP028: *N*-(2-hydroxybenzyl)-3-(3-methyl-3H-diazirin-3-yl)propanamide (0.039 mmol, 9.2 mg, 26%) as an off-white solid. ^{1}H NMR (300 MHz, $CDCl_3$) δ 9.11 (s, 1H), 7.17 – 6.77 (m, 3H), 6.35 (s, 1H), 4.38 (s, 2H), 2.05 (s, 2H), 1.79 (s, 2H), 1.01 (s, 3H). LC-MS: t_R = 0.86 min, 88% by UV, HRMS m/z: [M + H]$^+$ calcd for $C_{12}H_{16}N_3O_2$, 234.1242; found, 234.1246

PhP029: 3-(3-methyl-3H-diazirin-3-yl)-N-(1-(3-oxo-3,4-dihydropyrazin-2-yl)piperidin-3-yl)propanamide (0.147 mmol, 45 mg, 98%) as an yellow solid. ^{1}H NMR (300 MHz, $CDCl_3$) δ 11.10 (s, 1H), 7.04 (d, *J* = 4.2 Hz, 1H), 6.82 – 6.57 (m, 2H), 4.14 – 3.92 (m, 3H), 3.46 – 3.22 (m, 2H), 2.00 (d, *J* = 8.6 Hz, 2H), 1.82 – 1.64 (m, 6H), 1.02 (s, 3H). LC-MS: t_R = 0.59 min, 98% by UV, HRMS m/z: [M + H]$^+$ calcd for $C_{14}H_{21}N_6O_2$, 305.1725; found, 305.1725.

PhP030: *N*-((5-methyl-1H-benzo[d]imidazol-2-yl)methyl)-3-(3-methyl-3H-diazirin-3-yl)propanamide (0.151 mmol, 41 mg, 100%) as an off-white solid. ^{1}H NMR (300 MHz, DMSO-d_6) δ 8.76 (s, 1H), 7.59 (d, *J* = 8.3 Hz, 1H), 7.49 (s, 1H), 7.27 (d, *J* = 8.3 Hz, 1H), 4.63 (s, 2H), 2.46 (s, 3H), 2.13 (t, *J* = 7.6 Hz, 2H), 1.61 (t, *J* = 7.8 Hz, 2H), 0.99 (s, 3H). LC-MS: t_R = 0.49 min, 100% by UV, HRMS m/z: [M + H]$^+$ calcd for $C_{14}H_{18}N_5O$, 272.1511; found, 272.1517.

PhP031: *N*-(2-(1H-benzo[d]imidazol-2-yl)ethyl)-3-(3-methyl-3H-diazirin-3-yl)propanamide (0.148 mmol, 40.1 mg, 99%) as an off-white solid. ^{1}H NMR (500 MHz, DMSO-d_6) δ 8.57 – 8.39 (m, 2H), 7.83 (s, 1H), 7.63 (d, *J* = 8.2 Hz, 2H), 7.42 (s, 2H), 7.26 (s, 1H), 4.39 – 4.24 (m, 2H), 2.11 – 2.00 (m, 2H), 1.71 – 1.57 (m, 2H), 1.01 (s, 3H). LC-MS: t_R = 0.41 min, 97% by UV, HRMS m/z: [M + H]$^+$ calcd for $C_{14}H_{18}N_5O$, 272.1511; found, 272.1516.

PhP032: *N*-(1-(2-methoxyphenyl)propyl)-3-(3-methyl-3H-diazirin-3-yl)propanamide (0.106 mmol, 29.2 mg, 71%) as an off-white solid. ^{1}H NMR (300 MHz, $CDCl_3$) δ 7.25 – 7.12 (m, 2H), 6.98 – 6.79 (m, 2H), 6.46 (d, *J* = 9.4 Hz, 1H), 4.99 (q, *J* = 8.1 Hz, 1H), 3.88 (s, 3H), 2.04 – 1.90 (m, 2H), 1.88 – 1.66 (m, 4H), 0.98 (s, 3H), 0.87 – 0.72 (m, 3H)
LC-MS: t_R = 1.08 min, 96% by UV, HRMS m/z: $[M + H]^+$ calcd for $C_{15}H_{22}N_3O_2$, 276.1712; found, 276.1712.

PhP033: *N*-(4-(1H-imidazol-1-yl)benzyl)-3-(3-methyl-3H-diazirin-3-yl)propanamide (0.101 mmol, 29 mg, 67%) as an off-white solid. ^{1}H NMR (500 MHz, DMSO-d_6) δ 8.47 (d, *J* = 29.9 Hz, 2H), 7.83 (s, 2H), 7.63 (d, *J* = 8.0 Hz, 2H), 7.48 – 7.19 (m, 2H), 4.40 – 4.24 (m, 2H), 2.12 – 1.99 (m, 2H), 1.70 – 1.53 (m, 2H), 1.01 (s, 3H).
LC-MS: t_R = 0.39 min, 99% by UV, HRMS m/z: $[M + H]^+$ calcd for $C_{15}H_{18}N_5O$, 284.1511; found, 284.1519.

PhP034: *N*-(2-(4-methoxynaphthalen-1-yl)ethyl)-3-(3-methyl-3H-diazirin-3-yl)propanamide (0.060 mmol, 18.6 mg, 40%) as an off-white solid. ^{1}H NMR (300 MHz, $CDCl_3$) δ 8.31 (d, J = 7.9 Hz, 1H), 8.00 (d, J = 7.9 Hz, 1H), 7.60 – 7.45 (m, 2H), 7.23 (s, 1H), 6.75 (d, J = 7.4 Hz, 1H), 5.43 (s, 1H), 4.01 (s, 3H), 3.59 (d, J = 10.1 Hz, 2H), 3.28 – 3.15 (m, 2H), 1.90 (d, J = 9.3 Hz, 2H), 1.75 (d, J = 9.4 Hz, 2H), 1.01 (s, 3H). LC-MS: t_R = 1.16 min, 100% by UV, HRMS m/z: $[M + H]^+$ calcd for $C_{18}H_{22}NO_2$, 284.165; found, 284.1651.

PhP035: 3-(3-methyl-3H-diazirin-3-yl)-N-(2-((3-nitrophenyl)sulfonamido)ethyl)propanamide (0.120 mmol, 42.5 mg, 80%) as an off-white solid. ^{1}H NMR (300 MHz, $CDCl_3$) δ 8.69 (s, 1H), 8.43 (d, *J* = 7.9 Hz, 1H), 8.20 (d, 1H), 7.84 – 7.68 (m, 1H), 6.12 – 5.92 (m, 2H), 3.49 – 3.33 (m, 2H), 3.26 – 3.10 (m, 2H), 2.07 – 1.93 (m, 2H), 1.85 – 1.73 (m, 2H), 1.03 (s, 3H).
LC-MS: t_R = 0.88 min, 93% by UV, HRMS m/z: $[M + H]^+$ calcd for $C_{13}H_{18}N_5O_5S$, 356.1028; found, 356.1029

PhP036: methyl (3-(3-methyl-3H-diazirin-3-yl)propanoyl)-D-tryptophanate (0.067 mmol, 22.1 mg, 45%) as an off-white solid. ^{1}H NMR (500 MHz, DMSO-d_6) δ 10.86 (s, 1H), 8.34 (d, *J* = 7.5 Hz, 1H), 7.49 (d, *J* = 7.8 Hz, 1H), 7.33 (d, *J* = 8.0 Hz, 1H), 7.17 – 7.12 (m, 1H), 7.07 (t, *J* = 7.6 Hz, 1H), 6.99 (t, *J* = 7.5 Hz, 1H), 4.51 (q, *J* = 7.2 Hz, 1H), 3.58 (s, 3H), 3.14 (dd, *J* = 14.7, 5.4 Hz, 1H), 3.03 (dd, *J* = 14.7, 8.8 Hz, 1H), 2.07 – 1.96 (m, 2H), 1.48 (q, *J* = 7.9 Hz, 2H), 0.93 (s, 3H). LC-MS: t_R = 0.98 min, 85% by UV, HRMS m/z: $[M + H]^+$ calcd for $C_{17}H_{21}N_4O_3$, 329.1613; found, 329.1616

PhP037: 3-(3-methyl-3H-diazirin-3-yl)-N-((5-(m-tolyl)-1H-imidazol-2-yl)methyl)propanamide (0.118 mmol, 35.2 mg, 79%) as an off-white solid. ^{1}H NMR (300 MHz, $CDCl_3$) δ 7.34 (d, *J* = 6.6 Hz, 2H), 7.28 (d, *J* = 7.8 Hz, 1H), 7.25 (d, *J* = 4.5 Hz, 1H), 7.15 (d, *J* = 7.5 Hz, 1H), 4.65 (s, 2H), 3.49 (d, *J* = 1.4 Hz, 1H), 2.34 (s, 3H), 2.22 – 2.11 (m, 2H), 1.66 (t, *J* = 7.4 Hz, 2H), 0.95 (s, 3H). LC-MS: t_R = 0.58 min, 97% by UV, HRMS m/z: $[M + H]^+$ calcd for $C_{16}H_{20}N_5O$, 298.1667; found, 298.1675.

PhP038: *N*-(1-(2,5-dimethoxyphenyl)-1-hydroxypropan-2-yl)-3-(3-methyl-3H-diazirin-3-yl)propanamide (0.135 mmol, 43.4 mg, 90%) as an off-white solid. ^{1}H NMR (300 MHz, DMSO-d_6) δ 7.59 (d, *J* = 7.9 Hz, 1H), 7.00 – 6.91 (m, 1H), 6.91 – 6.82 (m, 1H), 6.80 – 6.70 (m, 1H), 5.24 – 5.14 (m, 1H), 4.84 – 4.77 (m, 1H), 4.11 – 3.97 (m, 1H), 3.69 (s, 6H), 2.02 – 1.87 (m, 2H), 1.47 (q, *J* = 6.8 Hz, 2H), 1.03 – 0.89 (m, 3H), 0.83 (s, 3H).
LC-MS: t_R = 0.86 min, 93% by UV, HRMS m/z: $[M + H]^+$ calcd for $C_{16}H_{24}N_3O_4$, 322.1766; found, 322.1762.

PhP039: *N*-((6-methoxypyridin-3-yl)methyl)-3-(3-methyl-3H-diazirin-3-yl)propanamide (0.024 mmol, 6 mg, 16%) as an off-white solid. ^{1}H NMR (300 MHz, $CDCl_3$) δ 8.01 (s, 1H), 7.60 – 7.36 (m, 1H), 6.77 – 6.54 (m, 1H), 6.01 (s, 1H), 4.38 – 4.05 (m, 2H), 3.88 (s, 3H), 2.06 – 1.81 (m, 2H), 1.79 – 1.61 (m, 3H), 0.99 (s, 3H). LC-MS: t_R = 0.72 min, 87% by UV, HRMS m/z: $[M + H]^+$ calcd for $C_{12}H_{17}N_4O_2$, 249.1351; found, 249.1354.

PhP040: *N*-((1H-indol-2-yl)methyl)-3-(3-methyl-3H-diazirin-3-yl)propanamide (0.126 mmol, 32.3 mg, 84%) as an off-white solid. ^{1}H NMR (300 MHz, $CDCl_3$) δ 8.91 (s, 1H), 7.59 – 7.49 (m, 1H), 7.34 (d, *J* = 10.1 Hz, 1H), 7.22 – 7.04 (m, 2H), 6.32 (s, 1H), 6.03 (s, 1H), 4.58 – 4.33 (m, 2H), 2.09 – 1.93 (m, 2H), 1.79 (d, *J* = 8.7 Hz, 2H), 1.00 (s, 3H).LC-MS: t_R = 0.98 min, 88% by UV, HRMS m/z: $[M + H]^+$ calcd for $C_{14}H_{17}N_4O$, 257.1402; found, 257.1404.

PhP041: *N*-(1-(4-fluorophenyl)-6-methyl-1H-pyrazolo[3,4-d]pyrimidin-4-yl)-3-(3-methyl-3H-diazirin-3-yl)propanamide (0.040 mmol, 14.2 mg, 27%) as an yellow solid. ^{1}H NMR (300 MHz, $CDCl_3$) δ 8.79 – 8.71 (m, 1H), 8.28 – 8.13 (m, 3H), 7.22 – 7.18 (m, 2H), 2.70 (s, 3H), 2.42 – 2.31 (m, 2H), 2.00 – 1.89 (m, 2H), 1.11 (s, 3H).LC-MS: t_R = 1.26 min, 99% by UV, HRMS m/z: $[M + H]^+$ calcd for $C_{17}H_{17}N_7OF$, 354.1478; found, 354.1489.

PhP042: *N*-(3-(1H-tetrazol-1-yl)phenyl)-3-(3-methyl-3H-diazirin-3-yl)propanamide (0.076 mmol, 20.7 mg, 51%) as an off-white solid. ^{1}H NMR (300 MHz, DMSO-d_6) δ 10.41 (s, 1H), 10.07 (s, 1H), 8.28 (q, *J* = 1.4 Hz, 1H), 7.72 – 7.62 (m, 1H), 7.60 – 7.53 (m, 2H), 2.26 (t, *J* = 7.6 Hz, 2H), 1.71 (dd, *J* = 8.1, 6.9 Hz, 2H), 1.03 (s, 3H). LC-MS: t_R = 0.85 min, 100% by UV, HRMS m/z: $[M + H]^+$ calcd for $C_{12}H_{14}N_7O$, 272.1259; found, 272.1263.

PhP043: *N*-(5-(1-methyl-1H-imidazol-2-yl)-1H-pyrazol-3-yl)-3-(3-methyl-3H-diazirin-3-yl)propanamide (0.041 mmol, 11.1 mg, 27%) as an off-white solid. ^{1}H NMR (300 MHz, DMSO-d_6) δ 10.97 (s, 1H), 8.01 – 7.49 (m, 3H), 6.80 (s, 1H), 4.06 (s, 3H), 2.27 (d, *J* = 7.2 Hz, 2H), 1.80 – 1.66 (m, 2H), 1.02 (s, 3H). LC-MS: t_R = 0.4 min, 100% by UV, HRMS m/z: $[M + H]^+$ calcd for $C_{12}H_{16}N_7O$, 274.1416; found, 274.1423.

PhP044: *N*-(2-(2-methoxyphenoxy)ethyl)-3-(3-methyl-3H-diazirin-3-yl)propanamide (0.134 mmol, 37.3 mg, 89%) as an off-white solid. ^{1}H NMR (300 MHz, $CDCl_3$) δ 6.92 (q, *J* = 5.8 Hz, 4H), 6.24 (s, 1H), 4.08 (t, *J* = 4.7 Hz, 2H), 3.87 (s, 3H), 3.64 (q, *J* = 4.9 Hz, 2H), 2.01 (d, *J* = 6.4 Hz, 2H), 1.75 (d, *J* = 6.3 Hz, 2H), 1.00 (s, 3H). LC-MS: t_R = 0.9 min, 84% by UV, HRMS m/z: $[M + H]^+$ calcd for $C_{14}H_{20}N_3O_3$, 278.1504; found, 278.1508.

PhP045: *N*-(2-methyl-2-(methylsulfonamido)propyl)-3-(3-methyl-3H-diazirin-3-yl)propanamide (0.132 mmol, 36.5 mg, 88%) as an off-white solid. ^{1}H NMR (300 MHz, $CDCl_3$) δ 6.43 (s, 1H), 5.24 (s, 1H), 3.36 (dd, *J* = 6.4, 2.8 Hz, 2H), 3.04 (s, 3H), 2.07 – 1.99 (m, 2H), 1.77 (td, *J* = 7.7, 3.0 Hz, 2H), 1.35 (s, 6H), 1.02 (s, 3H).
LC-MS: t_R = 0.64 min, 91% by UV, HRMS m/z: $[M + H]^+$ calcd for $C_{10}H_{20}N_4O_3S$, 277.1256; found, 277.1257.

PhP046: *N*-((4-isobutylmorpholin-2-yl)methyl)-3-(3-methyl-3H-diazirin-3-yl)propanamide (0.138 mmol, 39.1 mg, 92%) as an off-white solid. ^{1}H NMR (500 MHz, $CDCl_3$) δ 7.80 (s, 1H), 5.38 (d, *J* = 32.4 Hz, 1H), 4.03 (s, 1H), 3.47 (s, 1H), 2.90 (s, 2H), 2.33 – 2.21 (m, 9H), 2.17 (s, 2H), 2.05 (t, *J* = 7.7 Hz, 2H), 1.78 (d, *J* = 8.9 Hz, 2H), 1.03 (d, *J* = 15.9 Hz, 5H). LC-MS: t_R = 0.37 min, 90% by UV, HRMS m/z: $[M + H]^+$ calcd for $C_{14}H_{27}N_4O_2$, 283.2134; found, 283.2138

PhP047: 3-(3-methyl-3H-diazirin-3-yl)-N-(5,6,7,8-tetrahydroimidazo[1,2-a]pyridin-6-yl)propanamide (0.059 mmol, 14.6 mg, 39%) as an off-white solid. ^{1}H NMR (300 MHz, DMSO-d_6) δ 8.16 (d, *J* = 6.6 Hz, 1H), 7.56 (d, *J* = 10.2 Hz, 2H), 4.28 (d, *J* = 12.9 Hz, 2H), 3.95 – 3.86 (m, 1H), 3.04 (d, *J* = 6.9 Hz, 2H), 1.98 (q, *J* = 7.5 Hz, 4H), 1.65 – 1.54 (m, 2H), 0.96 (s, 3H). LC-MS: t_R = 1.04 min, 86% by UV, HRMS m/z: $[M + H]^+$ calcd for $C_{12}H_{18}N_5O$, 248.1511; found, 248.1510.

PhP048: *N*-(3-(ethyl(phenyl)amino)propyl)-3-(3-methyl-3H-diazirin-3-yl)propanamide (0.126 mmol, 36.3 mg, 84%) as an off-white solid. ^{1}H NMR (300 MHz, DMSO-d_6) δ 7.90 (s, 1H), 7.12 (s, 2H), 6.64 (d, *J* = 6.9 Hz, 2H), 6.54 (s, 1H), 3.24 (s, 2H), 3.09 (s, 2H),2.57-2.54 (m, 2H) 1.96 (s, 2H), 1.60 (d, *J* = 9.9 Hz, 4H), 1.24 (s, 1H), 1.05 (t, *J* = 3.6 Hz, 3H), 0.99 (t, *J* = 3.1 Hz, 3H).

LC-MS: t_R = 0.54 min, 83% by UV, HRMS m/z: $[M + H]^+$ calcd for $C_{16}H_{24}N_4O$, 289.2028; found, 289.2032.

PhP049: 6-(tert-butyl)-2-(3-(3-methyl-3H-diazirin-3-yl)propanamido)-4,5,6,7-tetrahydrobenzo[b]thiophene-3-carboxamide (0.046 mmol, 16.7 mg, 31%) as an off-white solid.

^{1}H NMR (500 MHz, DMSO-d_6) δ 2.76 (dd, *J* = 15.6, 4.9 Hz, 1H), 2.71 – 2.61 (m, 2H), 2.43-2.30 (m, 3H), 1.97 (d, *J* = 12.8 Hz, 1H), 1.66 (t, *J* = 7.6 Hz, 2H), 1.47 – 1.37 (m, 1H), 1.26 – 1.18 (m, 1H), 1.01 (s, 3H), 0.91 (s, 9H).

LC-MS: t_R = 1.34 min, 100% by UV, HRMS m/z: $[M + H]^+$ calcd for $C_{18}H_{27}N_4O_2S$, 363.1854; found, 363.1855.

PhP050: *N*-(3-(1H-imidazol-1-yl)propyl)-3-(3-methyl-3H-diazirin-3-yl)propanamide (0.066 mmol, 15.6 mg, 44%) as an off-white solid. ^{1}H NMR (300 MHz, DMSO-d_6) δ 9.07 (s, 1H), 7.95 (s, 1H), 7.78 (s, 1H), 7.70 (s, 1H), 4.18 (t, *J* = 6.9 Hz, 2H), 3.03 (q, *J* = 6.5 Hz, 2H), 1.95 (q, *J* = 7.4 Hz, 4H), 1.59 (t, *J* = 7.6 Hz, 2H), 1.25 (s, 4H), 0.99 (s, 3H). LC-MS: t_R = 1.04 min, 97% by UV, HRMS m/z: $[M + H]^+$ calcd for $C_{11}H_{18}N_5O$, 236.1511; found, 236.1511.

PhP051: *N*-[2-(benzylsulfanyl)ethyl]-3-(3-methyl-3H-diazirin-3-yl)propanamide (0.059 mmol, 16.4 mg, 39 %) ^{1}H NMR (300 MHz, $CDCl_3$) δ 8.02 (s, 1H), 7.47 (s, 2H), 7.18 – 6.90 (m, 3H), 5.11 (s, 2H), 3.58 (q, *J* = 6.5 Hz, 2H), 2.93 (t, *J* = 6.7 Hz, 2H), 1.95 – 1.85 (m, 2H), 1.76 – 1.69 (m, 2H), 0.99 (s, 3H). LC-MS: t_R = 0.81 min, 95% by UV, HRMS m/z: $[M + H]^+$ calcd for $C_{16}H_{18}N_5O$, 278,1248; found, 278,1544.

PhP052: 3-(3-methyl-3H-diazirin-3-yl)-N-(4-(pyrimidin-5-yl)benzyl)propanamide (0.018 mmol, 5.4 mg, 12%) as an off-white solid. ^{1}H NMR (500 MHz, DMSO-d_6) δ 9.17 (s, 1H), 9.12 (s, 2H), 8.41 (t, *J* = 6.1 Hz, 1H), 7.76 (d, *J* = 7.8 Hz, 2H), 7.42 (d, *J* = 7.7 Hz, 2H), 4.33 (d, *J* = 5.9 Hz, 2H), 2.06 (t, *J* = 7.7 Hz, 2H), 1.63 (t, *J* = 7.7 Hz, 2H), 1.00 (s, 3H)

; LC-MS: t_R = 0.78 min, 90% by UV, HRMS m/z: $[M + H]^+$ calcd for $C_{16}H_{18}N_5O$, 296.1511; found, 296.1517.

PhP053: *N*-((1H-imidazo[4,5-b]pyridin-2-yl)methyl)-3-(3-methyl-3H-diazirin-3-yl)propanamide (0.126 mmol, 32.5 mg, 84%) as an off-white solid. ^{1}H NMR (300 MHz, DMSO-d_6) δ 8.72 (d, *J* = 5.4 Hz, 1H), 8.57 – 8.47 (m, 1H), 8.27 (d, *J* = 7.6 Hz, 1H), 7.50 (p, *J* = 4.3 Hz, 1H), 4.64 (d, *J* = 5.0 Hz, 2H), 2.14 (t, *J* = 7.6 Hz, 2H), 1.61 (q, *J* = 6.2 Hz, 2H), 0.99

(s, 3H). LC-MS: t_R = 0.42 min, 98% by UV, HRMS m/z: $[M + H]^+$ calcd for $C_{12}H_{15}N_6O$, 259.1307; found, 259.1310.

PhP054: 2-(3-(3-methyl-3H-diazirin-3-yl)propanamido)-5-(piperidin-1-yl)benzamide (0.130 mmol, 42.8 mg, 87%) as an off-white solid. ^1H NMR (500 MHz, DMSO-d_6) δ 9.05 (s, 1H), 8.06 (s, 1H), 7.78 (d, *J* = 8.9 Hz, 2H), 7.45 (s, 1H), 3.07 (s, 5H), 2.21 (t, *J* = 7.6 Hz, 2H), 1.82 – 1.48 (m, 9H), 1.02 (s, 3H).

LC-MS: t_R = 0.57 min, 100% by UV, HRMS m/z: $[M + H]^+$ calcd for $C_{17}H_{24}N_5O_2$, 330.193; found, 330.1938.

PhP055: *N*-(2-((dimethylamino)methyl)benzo[d]oxazol-6-yl)-3-(3-methyl-3H-diazirin-3-yl)propanamide (0.122 mmol, 36.8 mg, 81%) as an off-white solid. ^1H NMR (600 MHz, DMSO-d_6) δ ppm 1.02 (s, 3 H), 1.66 (br t, *J* = 7.52 Hz, 2 H), 2.09 - 2.21 (m, 2 H), 2.98 (s, 6 H), 4.68 - 4.74 (s, 2 H), 6.78 - 6.84 (m, 2 H), 6.96 (br d, *J* = 8.44 Hz, 2 H), 7.15-7.20 (s, 1 H), 9.73 - 9.76 (br s, 1 H); LC-MS: t_R = 0.45 min, 93% by UV, HRMS m/z: $[M + H]^+$ calcd for $C_{15}H_{20}N_5O_2$, 302.1617; found, 302.1620.

PhP056: *tert*-butyl 4-(3-(3-(3-methyl-3H-diazirin-3-yl)propanamido)propyl)piperazine-1-carboxylate (0,105 mmol, 33 mg, 89%) an an off-white solid. ^1H NMR (300 MHz, $CDCl_3$) δ 7.07 (s, 1H), 4.65 (s, 5H), 3.76 (s, 3H), 3.36 (d, *J* = 6.0 Hz, 2H), 3.18 (s, 4H), 2.24 – 2.18 (m, 2H), 2.09 – 2.00 (m, 2H), 1.79 – 1.68 (m, 2H), 1.47 (s, 9H), 1.04 (s, 3H). LC-MS: t_R = 1.34 min, 95% by UV, HRMS m/z: $[M + H]^+$ calcd for $C_{17}H_{32}N_5O_3$, 354.2505; found, 354.2510.

PhP057: N-((2-hydroxynaphthalen-1-yl)methyl)-3-(3-methyl-3H-diazirin-3-yl)propanamide (0,132 mmol, 32 mg, 86%) as an off-white solid. ^1H NMR (500 MHz, $CDCl_3$) δ 9.72 (s, 1H), 7.75 (d, *J* = 30.4 Hz, 3H), 7.50 (s, 1H), 7.34 (d, *J* = 10.3 Hz, 1H), 7.23 (d, *J* = 15.9 Hz, 1H), 6.47 (s, 1H), 4.78 (s, 2H), 2.01 (s, 2H), 1.74 (d, *J* = 9.6 Hz, 2H), 1.01 – 0.82 (m, 3H). LC-MS: t_R = 1.28 min, 98% by UV, HRMS m/z: $[M + H]^+$ calcd for $C_{16}H_{18}N_3O_2$, 284.1399; found, 284.1404.

PhP058: (S)-N-(1-amino-3-(4-hydroxyphenyl)-1-oxopropan-2-yl)-3-(3-methyl-3H-diazirin-3-yl)propanamide (0.035 mmol, 10.3 mg, 23%) as an off-white solid. ^1H NMR (300 MHz, DMSO-d_6) δ 9.14 (s, 1H), 7.96 (s, 1H), 7.35 (s, 1H), 7.00 (dd, *J* = 8.6, 2.4 Hz, 3H), 6.62 (dd, *J* = 8.5, 2.4 Hz, 2H), 4.33 (s, 1H), 2.87 (dt, *J* = 13.6, 3.3 Hz, 1H), 2.61 (dd, *J* = 13.3, 9.5 Hz, 1H), 2.01 – 1.80 (m, 2H), 1.47 – 1.27 (m, 2H), 0.89 (s, 3H).

LC-MS: t_R = 0.59 min, 100% by UV, HRMS m/z: $[M + H]^+$ calcd for $C_{14}H_{19}N_4O_3$, 291.1457; found, 291.1463.

PhP059: 3-((3-(3-methyl-3H-diazirin-3-yl)propanamido)methyl)benzamide (0.076 mmol, 19.7 mg, 51%) as an off-white solid. ^{1}H NMR (300 MHz, $CDCl_3$) δ 7.75 (s, 2H), 7.44 (s, 2H), 5.84 (s, 1H), 4.49 (s, 2H), 2.04 (s, 2H), 1.84 (s, 2H), 1.27 (s, 2H), 1.04 (s, 3H).
; LC-MS: t_R = 0.62 min, 94% by UV, HRMS m/z: $[M + H]^+$ calcd for $C_{13}H_{17}N_4O_2$, 261.1351; found, 261.1346

PhP060: *N*-(4-(3-hydroxypyridin-2-yl)phenyl)-3-(3-methyl-3H-diazirin-3-yl)propanamide (0.143 mmol, 42.5 mg, 95%) as an off-white solid. ^{1}H NMR (300 MHz, DMSO-d_6) δ 11.75 (bs, 1H), 10.25 (s, 1H), 7.88 – 7.68 (m, 7H), 2.26 (s, 2H), 1.70 (d, *J* = 7.9 Hz, 2H), 1.03 (s, 3H).
LC-MS: t_R = 0.52 min, 98% by UV, HRMS m/z: $[M + H]^+$ calcd for $C_{16}H_{17}N_4O_2$, 297.1351; found, 297.1361

PhP061: *N*-(2-(3,5-dimethyl-1H-pyrazol-4-yl)ethyl)-3-(3-methyl-3H-diazirin-3-yl)propanamide (0.150 mmol, 37.4 mg, 100%) as an off-white solid. ^{1}H NMR (300 MHz, $CDCl_3$) δ 5.35 (s, 1H), 3.26 (s, 1H), 3.02 (d, *J* = 34.1 Hz, 2H), 2.89 (s, 1H), 2.62 (s, 2H), 2.28 (d, *J* = 37.0 Hz, 2H), 2.01 (bs, 2H), 1.26 (s, 9H).
LC-MS: t_R = 0.41 min, 87% by UV, HRMS m/z: $[M + H]^+$ calcd for $C_{12}H_{20}N_5O$, 250.1667; found, 250.1673.

PhP062: *N*-(1-(2,5-dimethoxyphenyl)ethyl)-3-(3-methyl-3H-diazirin-3-yl)propanamide (0.117 mmol, 34.1 mg, 78%) as an off-white solid. ^{1}H NMR (300 MHz, $CDCl_3$) δ 6.84 – 6.72 (m, 3H), 6.51 – 6.38 (m, 1H), 5.26 – 5.12 (m, 1H), 3.83 (s, 3H), 3.75 (s, 3H), 1.97 (dt, *J* = 8.1, 4.0 Hz, 2H), 1.75 – 1.69 (m, 2H), 1.43 (d, *J* = 1.8 Hz, 3H), 0.99 (s, 3H). LC-MS: t_R = 1.00 min, 93% by UV, HRMS m/z: $[M + H]^+$ calcd for $C_{15}H_{22}N_3O_3$, 292.1661; found, 292.1671.

PhP063: 3-(3-methyl-3H-diazirin-3-yl)-N-(2-(4-methyl-4H-1,2,4-triazol-3-yl)ethyl)propanamide (0.077 mmol, 18.3 mg, 51%) as an off-white solid. ^{1}H NMR (300 MHz, DMSO-d_6) δ 9.02 (s, 1H), 8.04 (s, 1H), 3.71 (s, 3H), 3.00 – 2.94 (m, 2H), 1.89 (s, 4H), 1.48 (s, 2H), 0.92 (s, 3H).
LC-MS: t_R = 0.39 min, 89% by UV, HRMS m/z: $[M + H]^+$ calcd for $C_{10}H_{17}N_6O$, 237.1463; found, 237.1459

PhP064: *N*-(1-hydroxy-3-(1H-indol-3-yl)propan-2-yl)-3-(3-methyl-3H-diazirin-3-yl)propanamide (0.032 mmol, 9.5 mg, 21%) as an off-white solid. ^{1}H NMR (500 MHz, CD_3CN) δ 9.09 (s, 1H), 7.63 (d, *J* = 7.8 Hz, 1H), 7.38 (dd, *J* = 8.2, 1.1 Hz, 1H), 7.14 – 7.02 (m, 3H), 6.35 (d, *J* = 8.0 Hz, 1H), 4.20 – 4.04 (m, 1H), 3.58 – 3.41 (m, 2H), 2.95 (dd, *J* = 14.6, 6.6 Hz, 1H), 2.85 (dd, *J* = 14.5, 7.2 Hz, 1H), 2.00 – 1.92 (m, 3H), 1.61 – 1.50 (m, 2H), 0.94 (s, 3H).

LC-MS: t_R = 0.81 min, 100% by UV, HRMS m/z: $[M + H]^+$ calcd for $C_{17}H_{24}N_6OF_3$, 301.1664; found, 301.1669.

PhP065: *N*-(2-((2-methyl-1H-indol-3-yl)thio)ethyl)-3-(3-methyl-3H-diazirin-3-yl)propanamide (0.062 mmol, 19.5 mg, 41%) as an off-white solid. ^{1}H NMR (300 MHz, DMSO-*d*$_6$) δ 11.33 (d, *J* = 8.8 Hz, 1H), 7.98 – 7.83 (m, 1H), 7.57 – 7.43 (m, 1H), 7.29 (q, *J* = 6.1 Hz, 1H), 7.03 (p, *J* = 6.2 Hz, 2H), 3.06 (q, *J* = 6.7 Hz, 2H), 2.59 (d, *J* = 9.8 Hz, 2H), 2.43 (s, 3H), 1.89 (t, *J* = 7.4 Hz, 2H), 1.50 (t, *J* = 7.2 Hz, 2H), 0.95 (s, 3H).LC-MS: t_R = 1.07 min, 100% by UV, HRMS m/z: $[M + H]^+$ calcd for $C_{16}H_{21}N_4OS$, 317.1436; found, 317.1444.

PhP066: *N*-(3-(1H-1,2,4-triazol-3-yl)propyl)-3-(3-methyl-3H-diazirin-3-yl)propanamide (0.147 mmol, 34.7 mg, 98%) as an off-white solid. ^{1}H NMR (500 MHz, DMSO-*d*$_6$) δ 8.37 (d, *J* = 2.9 Hz, 1H), 7.91 (d, *J* = 5.6 Hz, 1H), 3.09 (q, *J* = 6.6 Hz, 2H), 2.74 (dd, *J* = 8.9, 6.1 Hz, 2H), 1.95 (t, *J* = 7.6 Hz, 2H), 1.83 – 1.75 (m, 2H), 1.57 (dd, *J* = 8.7, 6.7 Hz, 2H), 0.98 (d, *J* = 3.0 Hz, 3H).

LC-MS: t_R = 0.46 min, 89% by UV, HRMS m/z: $[M + H]^+$ calcd for $C_{10}H_{17}N_6O$, 237.1463; found, 237.1465.

PhP067: *N*-(3-(5,6-dimethyl-1H-benzo[d]imidazol-1-yl)-2-hydroxypropyl)-3-(3-methyl-3H-diazirin-3-yl)propanamide (0.020 mmol, 6.7 mg, 13%) as an off-white solid. ^{1}H NMR (300 MHz, $CDCl_3$) δ 7.45 – 7.29 (m, 1H), 7.26 – 7.20 (m, 1H), 6.75 (s, 1H), 4.29 (d, *J* = 9.8 Hz, 1H), 4.16 (s, 2H), 3.66 – 3.49 (m, 1H), 3.33 – 3.18 (m, 1H), 2.36 (s, 3H), 2.29 (s, 4H), 2.06 (t, *J* = 7.5 Hz, 2H), 1.75 (t, *J* = 7.4 Hz, 2H), 1.02 (s, 3H). LC-MS: t_R = 053 min, 100% by UV, HRMS m/z: $[M + H]^+$ calcd for $C_{17}H_{24}N_5O_2$, 330.193; found, 330.1938.

PhP068: *N*-((2-methoxy-4,6-dimethylpyridin-3-yl)methyl)-3-(3-methyl-3H-diazirin-3-yl)propanamide (0.104 mmol, 28.8 mg, 69%) as an off-white solid. ^{1}H NMR (300 MHz, $CDCl_3$) δ 6.56 (s, 1H), 5.90 (s, 1H), 4.41 – 4.36 (m, 2H), 3.94 (s, 3H), 2.36 (d, *J* = 1.8 Hz, 6H), 1.98 – 1.91 (m, 2H), 1.76 – 1.69 (m, 2H), 0.98 (s, 3H).

LC-MS: t_R = 0.96 min, 97% by UV, HRMS m/z: $[M + H]^+$ calcd for $C_{14}H_{21}N_4O_2$, 277.1664; found, 277.1668.

PhP069: *N*-(3-(1H-benzo[d]imidazol-2-yl)propyl)-3-(3-methyl-3H-diazirin-3-yl)propanamide (0.143 mmol, 41 mg, 95%) as an off-white solid. ^{1}H NMR (300 MHz, $CDCl_3$) δ 13.32 (s, 1H), 7.77 (s, 2H), 7.57 (t, *J* = 4.3 Hz, 2H), 6.79 (s, 1H), 3.43 (d, *J* = 6.1 Hz, 2H), 3.31 (s, 2H), 2.96 (d, *J* = 9.8 Hz, 2H), 2.30 (t, *J* = 7.2 Hz, 2H), 1.84 (t, *J* = 7.5 Hz, 3H), 1.07 (s, 3H).

LC-MS: t_R = 0.45 min, 100% by UV, HRMS m/z: $[M + H]^+$ calcd for $C_{15}H_{20}N_5O$, 286.1667; found, 286.1673.

PhP070: -((1S,2R)-1-hydroxy-1-(3-hydroxyphenyl)propan-2-yl)-3-(3-methyl-3H-diazirin-3-yl)propanamide (0.032 mmol, 9 mg, 21%) as an off-white solid. ^{1}H NMR (700 MHz, DMSO-d_6) δ ppm 0.89 (d, *J* = 6.78 Hz, 3 H), 0.96 (s, 3 H), 1.46 - 1.53 (m, 2 H), 1.91 - 1.99 (m, 2 H), 3.83 - 3.92 (m, 1 H), 4.47 - 4.52 (m, 1 H), 6.57 - 6.61 (m, 1 H), 6.66 - 6.74 (m, 1 H), 6.76 (s, 1 H), 7.08 (br t, *J* = 7.84 Hz, 1 H), 7.74 - 7.79 (m, 1 H), 9.23 - 9.29 (br s, 1 H); LC-MS: t_R = 0.68 min, 99% by UV, HRMS m/z: $[M + H]^+$ calcd for $C_{14}H_{20}N_3O_3$, 278.1504; found, 278.1507.

PhP071: *N*-(2,4-dioxo-1,4-dihydroquinazolin-3(2H)-yl)-3-(3-methyl-3H-diazirin-3-yl)propanamide (0.115 mmol, 33.1 mg, 77%) as an off-white solid. ^{1}H NMR (300 MHz, DMSO-d_6) δ 10.61 (bs, 1H), 7.93 (t, *J* = 6.2 Hz, 1H), 7.78 – 7.66 (m, 1H), 7.24 (q, *J* = 6.9 Hz, 2H), 2.30 – 2.16 (m, 2H), 1.61 (q, *J* = 7.0 Hz, 2H), 1.05 (s, 3H). LC-MS: t_R = 0.67 min, 87% by UV, HRMS m/z: $[M + H]^+$ calcd for $C_{13}H_{14}N_5O_3$, 288.1096; found, 288.1098.

PhP072: *N*-((3-hydroxy-5-(hydroxymethyl)-2-methylpyridin-4-yl)methyl)-3-(3-methyl-3H-diazirin-3-yl)propanamide (0.031 mmol, 8.7 mg, 21%) as an off-white solid. ^{1}H NMR (300 MHz, DMSO-d_6) δ 8.95 (d, *J* = 6.2 Hz, 1H), 8.12 – 7.90 (m, 2H), 4.68 (s, 2H), 4.33 (d, *J* = 5.7 Hz, 2H), 2.46 (d, *J* = 2.3 Hz, 3H), 2.12 – 2.03 (m, 3H), 1.57 (t, *J* = 7.8 Hz, 2H), 0.94 (s, 3H). LC-MS: t_R = 0.4 min, 100% by UV, HRMS m/z: $[M + H]^+$ calcd for $C_{13}H_{19}N_4O_3$, 279.1457; found, 279.1468.

PhP073: *N*-(2-(2-methyl-1H-benzo[d]imidazol-1-yl)ethyl)-3-(3-methyl-3H-diazirin-3-yl)propanamide (0.120 mmol, 34.2 mg, 80%) as an off-white solid. ^{1}H NMR (300 MHz, CD_3CN) δ 7.83 – 7.55 (m, 4H), 6.58 (s, 1H), 4.50 – 4.34 (m, 2H), 3.63 (d, *J* = 5.3 Hz, 2H), 2.82 (s, 3H), 1.89 – 1.74 (m, 2H), 1.42 – 1.29 (m, 2H), 0.90 (s, 3H). LC-MS: t_R = 0.41 min, 95% by UV, HRMS m/z: $[M + H]^+$ calcd for $C_{15}H_{20}N_5O$, 286.1667; found, 286.1672.

PhP074: *N*-(4-methoxy-6-methylpyrimidin-2-yl)-3-(3-methyl-3H-diazirin-3-yl)propanamide (0.083 mmol, 20.6 mg, 55%) as an off-white solid. ^{1}H NMR (300 MHz, $CDCl_3$) δ 6.27 (s, 1H), 3.94 (s, 3H), 2.89 – 2.66 (m, 2H), 2.36 (s, 3H), 1.95 – 1.79 (m, 2H), 1.09 (s, 3H). LC-MS: t_R = 0.66 min, 90% by UV, HRMS m/z: $[M + H]^+$ calcd for $C_{11}H_{16}N_5O_2$, 250.1303; found, 250.1309.

PhP075: 3-(3-methyl-3H-diazirin-3-yl)-N-(2-oxo-1,2,3,4-tetrahydroquinolin-7-yl)propanamide (0.061 mmol, 16.5 mg, 41%) as an off-white solid. ^{1}H NMR (300 MHz, CD_3CN) δ 8.29 (s, 2H), 7.33 (s, 1H), 7.12 – 7.01 (m, 1H), 6.99 – 6.94 (m, 1H), 2.94 – 2.82 (m, 2H), 2.55 – 2.42 (m, 2H), 1.71 (s, 2H), 1.30 (d, *J* = 16.2 Hz, 2H), 1.02 (s, 3H).

LC-MS: t_R = 0.77 min, 97% by UV, HRMS m/z: $[M + H]^+$ calcd for $C_{14}H_{17}N_4O_2$, 273.1351; found, 273.1357.

PhP076: *N*-(6-ethyl-2-methylpyrimidin-4-yl)-3-(3-methyl-3H-diazirin-3-yl)propanamide (0.099 mmol, 24.4 mg, 66%) as an off-white solid. ^{1}H NMR (500 MHz, DMSO-d_6) δ 10.77 (s, 1H), 7.79 (s, 1H), 2.67 (q, *J* = 7.6 Hz, 2H), 2.57 – 2.51 (m, 3H), 2.34 (t, *J* = 7.4 Hz, 2H), 1.68 (t, *J* = 7.4 Hz, 2H), 1.21 (td, *J* = 7.6, 2.1 Hz, 3H), 1.03 (s, 3H). LC-MS: t_R = 0.63 min, 97% by UV, HRMS m/z: $[M + H]^+$ calcd for $C_{12}H_{18}N_5O$, 248.1511; found, 248.1516.

PhP077: *N*-(3-(5-methyl-2-oxo-2,3-dihydro-1H-imidazol-1-yl)benzyl)-3-(3-methyl-3H-diazirin-3-yl)propanamide (0.094 mmol, 30 mg, 63%) as an off-white solid. ^{1}H NMR (300 MHz, $CDCl_3$) δ 9.38 (s, 1H), 7.48 (s, 1H), 7.39 – 7.21 (m, 4H), 6.14 (s, 2H), 4.52 (s, 2H), 2.07 (s, 2H), 1.76 (d, *J* = 37.8 Hz, 4H), 1.07 (s, 3H). LC-MS: t_R = 0.72 min, 96% by UV, HRMS m/z: $[M + H]^+$ calcd for $C_{16}H_{20}N_5O_2$, 314.1617; found, 314.1616

PhP078: *N*-(2-((2,6-dimethylmorpholino)methyl)phenyl)-3-(3-methyl-3H-diazirin-3-yl)propanamide (0.083 mmol, 27.4 mg, 54%) as an off-white solid. ^{1}H NMR (300 MHz, $CDCl_3$) δ 8.35 (s, 1H), 7.52 – 7.28 (m, 4H), 4.13 (s, 2H), 3.82 – 3.70 (m, 2H), 3.32 (d, *J* = 12.1 Hz, 2H), 2.74 (d, *J* = 11.3 Hz, 2H), 2.53 (t, *J* = 6.9 Hz, 2H), 1.79 (t, *J* = 6.9 Hz, 2H), 1.24 (d, *J* = 6.2 Hz, 6H), 1.06 (s, 3H). LC-MS: t_R = 0.54 min, 98% by UV, HRMS m/z: $[M + H]^+$ calcd for $C_{18}H_{27}N_4O_2$, 331.2134; found, 331.2142.

PhP079: (S)-N-(1-(1,3-dioxoisoindolin-2-yl)-3-(3-fluorophenyl)propan-2-yl)-3-(3-methyl-3H-diazirin-3-yl)propanamide (0.140 mmol, 57.3 mg, 93%) as an yellow solid. ^{1}H NMR (300 MHz, CD_3CN) δ 7.84 – 7.72 (m, 4H), 7.28 (q, *J* = 7.4 Hz, 1H), 7.12 – 6.86 (m, 3H), 6.33 (d, *J* = 9.3 Hz, 1H), 4.52 – 4.37 (m, 1H), 3.76 – 3.66 (m, 2H), 3.01 – 2.67 (m, 2H), 1.85 – 1.74 (m, 2H), 1.37 – 1.26 (m, 2H), 0.80 (s, 3H). LC-MS: t_R = 1.11 min, 95% by UV, HRMS m/z: $[M + H]^+$ calcd for $C_{22}H_{22}N_4O_3F$, 409.1675; found, 409.1683.

PhP080: *N*-(4-(3,5-dimethylisoxazol-4-yl)benzyl)-3-(3-methyl-3H-diazirin-3-yl)propanamide (0.062 mmol, 19.4 mg, 41%) as an off-white solid. ^{1}H NMR (300 MHz, CD_3CN) δ 7.89 – 7.82 (m, 1H), 7.35 (d, *J* = 7.9 Hz, 2H), 7.25 (d, *J* = 7.9 Hz, 2H), 2.70 (s, 2H), 2.33 (s, 3H), 2.17 (s, 3H), 2.12 (d, *J* = 7.8 Hz, 2H), 1.62 (t, *J* = 7.9 Hz, 2H), 0.96 (s, 3H).

LC-MS: t_R = 0.99 min, 84.8% by UV, HRMS m/z: $[M + H]^+$ calcd for $C_{17}H_{21}N_4O_2$, 313.1664; found, 313.1669.

PhP081: *N*-(2-(5-(benzyloxy)-1H-indol-3-yl)ethyl)-3-(3-methyl-3H-diazirin-3-yl)propanamide (0.134 mmol, 50.3 mg, 89%) as an off-white solid. ^{1}H NMR (300 MHz, $CDCl_3$) δ 8.19 (s, 1H), 7.49 (d, *J* = 7.7 Hz, 2H), 7.44 – 7.30 (m, 3H), 7.12 (d, *J* = 4.1 Hz, 1H), 7.03 – 6.92 (m, 2H), 5.57 (s, 1H), 5.10 (s, 2H), 3.64 – 3.50 (m, 2H), 2.98 – 2.86 (m, 2H), 1.96 – 1.84 (m, 2H), 1.77 – 1.67 (m, 2H), 0.99 (s, 3H).

LC-MS: t_R = 1.16 min, 96% by UV, HRMS m/z: $[M + H]^+$ calcd for $C_{22}H_{25}N_4O_2$, 377.1977; found, 377.1986.

PhP082: 1-methyl-4-(3-(3-methyl-3H-diazirin-3-yl)propanamido)-3-propyl-1H-pyrazole-5-carboxamide (0.140 mmol, 40.9 mg, 93%) as an off-white solid. ^{1}H NMR (300 MHz, $CDCl_3$) δ 8.73 (s, 1H), 7.38 (s, 1H), 5.95 (s, 1H), 3.94 (s, 3H), 2.46 – 2.28 (m, 6H), 1.74 (s, 2H), 0.99 (s, 3H), 0.86 (s, 3H). LC-MS: t_R = 0.73 min, 85% by UV, HRMS m/z: $[M + H]^+$ calcd for $C_{13}H_{21}N_6O_2$, 293.1725; found, 293.1725.

PhP083: *N*-((6,7-dihydro-5H-pyrrolo[2,1-c][1,2,4]triazol-3-yl)methyl)-3-(3-methyl-3H-diazirin-3-yl)propanamide (0.133 mmol, 33 mg, 89%) as an off-white solid. ^{1}H NMR (500 MHz, DMSO-d_6) δ 8.50 (s, 1H), 4.37 (d, *J* = 5.5 Hz, 2H), 3.94 (t, *J* = 7.2 Hz, 2H), 2.84 (t, *J* = 7.6 Hz, 2H), 2.64 (q, *J* = 7.5 Hz, 2H), 2.03 (t, *J* = 7.8 Hz, 2H), 1.60 (t, *J* = 7.7 Hz, 2H), 0.99 (s, 3H). LC-MS: t_R = 0.42 min, 87% by UV, HRMS m/z: $[M + H]^+$ calcd for $C_{11}H_{17}N_6O$, 249.1463; found, 249.1464.

PhP084: *N*-(2-(3-(3-methyl-3H-diazirin-3-yl)propanamido)ethyl)isonicotinamide (0.087 mmol, 24 mg, 58%) as an off-white solid. ^{1}H NMR (300 MHz, $CDCl_3$) δ 8.74 (s, 2H), 7.69 (s, 3H), 6.26 (s, 1H), 3.57 (d, *J* = 19.8 Hz, 4H), 2.14 – 1.81 (m, 4H), 0.99 (s, 3H).

LC-MS: t_R = 0.45 min, 99% by UV, HRMS m/z: $[M + H]^+$ calcd for $C_{13}H_{18}N_5O_2$, 276.146; found, 276.1477.

PhP085: 3-(3-methyl-3H-diazirin-3-yl)-N-(4,5,6,7-tetrahydro-1H-benzo[d]imidazol-5-yl)propanamide (0.020 mmol, 5 mg, 13%) as an off-white solid. ^{1}H NMR (300 MHz, DMSO-d_6) δ 8.64 (s, 1H), 7.98 (s, 1H), 4.07 (s, 2H), 3.15 – 3.12 (m, 5H), 1.97 (t, *J* = 7.6 Hz, 2H), 1.58 (t, *J* = 7.6 Hz, 2H), 0.98 (s, 3H). LC-MS: t_R = 0.32 min, 86.4% by UV, HRMS m/z: $[M + H]^+$ calcd for $C_{12}H_{18}N_5O$, 248.1511; found, 248.1510.

PhP086: benzyl O-benzyl-N-(3-(3-methyl-3H-diazirin-3-yl)propanoyl)serinate (0.047 mmol, 18.5 mg, 31%) as an off-white solid. ^{1}H NMR (300 MHz, CD_3CN) δ 7.38 – 7.26 (m, 10H), 6.89 (d, *J* = 7.5 Hz, 1H), 5.22 – 5.08 (m, 2H), 4.66 (dt, *J* = 7.8, 3.8 Hz, 1H), 4.54 – 4.40 (m, 2H), 3.87 (dd, *J* = 9.7, 4.0 Hz, 1H), 3.66 (dd, *J* = 9.7, 3.5 Hz, 1H), 2.11 (dd, *J* = 8.4, 6.9 Hz, 2H), 1.60 (dd, *J* = 9.0, 7.1 Hz, 2H), 0.98 (s, 3H).

LC-MS: t_R = 1.25 min, 84.6% by UV, HRMS m/z: $[M + H]^+$ calcd for $C_{22}H_{26}N_3O_4$, 396.1923; found, 396.1933.

PhP087: *N*-(3-(N-(4-methoxyphenyl)sulfamoyl)phenyl)-3-(3-methyl-3H-diazirin-3-yl)propanamide (0.055 mmol, 21.2 mg, 37%) as an off-white solid. ^{1}H NMR (300 MHz, $CDCl_3$) δ 7.87 (t, *J* = 11.2 Hz, 2H), 7.38 – 7.29 (m, 2H), 7.09 (s, 1H), 7.01 – 6.93 (m, 2H), 6.74

(dd, *J* = 9.0, 1.7 Hz, 2H), 3.74 (s, 3H), 2.25 – 2.12 (m, 3H), 1.83 (t, *J* = 7.5 Hz, 2H), 1.05 (s, 3H). LC-MS: t_R = 1.04 min, 100% by UV, HRMS m/z: $[M + H]^+$ calcd for $C_{18}H_{21}N_4O_4S$, 389.1283; found, 389.1286.

PhP088: *N*-((4'-hydroxy-[1,1'-biphenyl]-4-yl)methyl)-3-(3-methyl-3H-diazirin-3-yl)propanamide (0.067 mmol, 20.6 mg, 45%) as an off-white solid. ^{1}H NMR (300 MHz, DMSO-d_6) δ 9.51 (s, 1H), 8.36 (s, 1H), 7.53 – 7.22 (m, 6H), 7.02 – 6.62 (m, 2H), 4.27 (d, *J* = 5.5 Hz, 2H), 2.04 (s, 2H), 1.79 – 1.50 (m, 2H), 1.00 (s, 3H).

LC-MS: t_R = 0.95 min, 100% by UV, HRMS m/z: $[M + H]^+$ calcd for $C_{17}H_{24}N_6OF_3$, 310.1555; found, 310.1562.

PhP089: 3-(3-methyl-3H-diazirin-3-yl)-N-(2-(pyrrolidin-1-ylmethyl)benzo[d]oxazol-6-yl)propanamide (0.122 mmol, 36.8 mg, 81%) as an off-white solid. ^{1}H NMR (300 MHz, DMSO-d_6) δ 10.03 (s, 1H), 8.03 (d, *J* = 5.0 Hz, 1H), 7.02 (s, 2H), 5.14 (s, 2H), 3.69 (dd, *J* = 21.3, 6.3 Hz, 4H), 2.20 (d, *J* = 7.0 Hz, 2H), 2.00 (s, 4H), 1.69 (q, *J* = 7.0 Hz, 2H), 1.02 (s, 3H). LC-MS: t_R = 0.45 min, 93% by UV, HRMS m/z: $[M + H]^+$ calcd for $C_{17}H_{22}N_5O_2$, 328.1773; found, 328.1761.

PhP090: 3-(3-methyl-3H-diazirin-3-yl)-N-(1-(5-methyl-4H-1,2,4-triazol-3-yl)ethyl)propanamide (0.144 mmol, 34 mg, 96%) as an off-white solid. ^{1}H NMR (500 MHz, CD_3CN) δ 6.36 (t, *J* = 49.4 Hz, 1H), 5.17 (s, 1H), 3.72 – 3.63 (m, 2H), 3.22 – 3.12 (m, 2H), 2.83 (s, 1H), 1.32 (s, 9H). LC-MS: t_R = 0.46 min, 88% by UV, HRMS m/z: $[M + H]^+$ calcd for $C_{10}H_{17}N_6O$, 237.1463; found, 237.1461.

PhP091: *N*-(2-(1-methyl-1H-imidazol-4-yl)ethyl)-3-(3-methyl-3H-diazirin-3-yl)propanamide (0.111 mmol, 26 mg, 74%) as an off-white solid. ^{1}H NMR (300 MHz, CD_3CN) δ 8.33 (s, 1H), 7.12 (s, 1H), 6.66 (s, 1H), 3.80 (d, *J* = 7.0 Hz, 3H), 3.42 (q, *J* = 6.5 Hz, 2H), 3.27 – 3.04 (m, 2H), 2.02 (d, *J* = 7.4 Hz, 2H), 1.67 – 1.56 (m, 2H), 0.97 (d, *J* = 2.4 Hz, 3H). LC-MS: t_R = 1.8 min, 9**2**% by **UV**, HRMS m/z: $[M + H]^+$ calcd for $C_{17}H_{24}N_6OF_3$, 236.1511; found, 236.1512.

PhP092: *N*-(2-(4,5-dimethylthiazol-2-yl)ethyl)-3-(3-methyl-3H-diazirin-3-yl)propanamide (0.091 mmol, 24 mg, 61%) as an off-white solid. ^{1}H NMR (500 MHz, CD_3CN) δ 6.67 (s, 1H), 3.45 (q, *J* = 6.5 Hz, 2H), 2.99 (q, *J* = 5.7 Hz, 2H), 2.28 (s, 3H), 2.22 (s, 3H), 1.99 (t, *J* = 7.6 Hz, 2H), 1.60 (q, *J* = 6.4 Hz, 2H), 0.97 (s, 3H).

LC-MS: t_R = 0.67 min, 98% by UV, HRMS m/z: $[M + H]^+$ calcd for $C_{12}H_{19}N_4OS$, 267.1280; found, 267.1279.

PhP093: 3-(3-methyl-3H-diazirin-3-yl)-N-((5-methyl-4H-1,2,4-triazol-3-yl)methyl)propanamide (0.139 mmol, 31 mg, 93%) as an off-white solid. ^{1}H NMR (300 MHz,

DMSO-d_6) δ 3.40 (p, J = 6.7 Hz, 2H), 2.91 (q, J = 7.3 Hz, 2H), 2.31 (s, 2H), 1.07 (s, 3H), 1.04 (s, 3H).

LC-MS: t_R = 0.43 min, 89% by UV, HRMS m/z: $[M + H]^+$ calcd for $C_9H_{15}N_6O$, 223.1307; found, 223.1304.

PhP094: 3-(3-methyl-3H-diazirin-3-yl)-N-(2-(3-phenylureido)ethyl)propanamide (0.074 mmol, 21.3 mg, 49%) as an off-white solid. ^{1}H NMR (300 MHz, CD_3CN) δ 7.39 (d, J = 7.7 Hz, 2H), 7.26 (d, J = 7.8 Hz, 3H), 6.97 (d, J = 6.9 Hz, 1H), 6.62 (s, 1H), 5.37 (s, 1H), 3.32 – 3.19 (m, 4H), 2.01 (d, J = 7.9 Hz, 2H), 1.63 (d, J = 8.1 Hz, 2H), 0.98 (s, 3H). LC-MS: t_R = 0.77 min, 100% by UV, HRMS m/z: $[M + H]^+$ calcd for $C_{14}H_{20}N_5O_2$, 290.1617 found, 290.1627.

PhP095: 1-methyl-N-(2-(3-(3-methyl-3H-diazirin-3-yl)propanamido)ethyl)-1H-pyrazole-5-carboxamide (0.139 mmol, 38.7 mg, 93%) as an off-white solid. ^{1}H NMR (300 MHz, DMSO-d_6) δ 8.44 (s, 1H), 7.98 (s, 1H), 7.44 (d, J = 3.6 Hz, 1H), 6.79 (d, J = 3.6 Hz, 1H), 4.04 (s, 4H), 3.26 – 3.17 (m, 5H), 1.97 (t, J = 7.4 Hz, 2H), 1.57 (t, J = 7.6 Hz, 2H), 0.98 (s, 3H).

LC-MS: t_R = 0.62 min, 96% by UV, HRMS m/z: $[M + H]^+$ calcd for $C_{12}H_{19}N_6O_2$, 279.1569; found, 279.1570.

PhP096: 3-(3-methyl-3H-diazirin-3-yl)-N-(2-(4-(5-(trifluoromethyl)pyridin-2-yl)piperazin-1-yl)ethyl)propanamide (0.030 mmol, 11.7 mg, 20%) as an orange solid. ^{1}H NMR (300 MHz, CD_3CN) δ 8.41 (s, 1H), 7.94 – 7.58 (m, 1H), 6.81 (d, J = 9.2 Hz, 1H), 6.50 (s, 1H), 3.90 – 3.50 (m, 5H), 2.74 – 2.43 (m, 8H), 2.04 (t, J = 7.8 Hz, 2H), 1.77 – 1.53 (m, 2H), 1.05 (s, 3H). LC-MS: t_R = 0.63 min, 89% by UV, HRMS m/z: $[M + H]^+$ calcd for $C_{17}H_{24}N_6OF_3$, 385.1963; found, 385.1970.

PhP097: 3-(3-methyl-3H-diazirin-3-yl)-N-(2-(2-phenylthiazol-4-yl)ethyl)propanamide (0.130 mmol, 41 mg, 87%) as an off-white solid. ^{1}H NMR (500 MHz, CD_3CN) δ 7.97 – 7.80 (m, 2H), 7.49 – 7.34 (m, 3H), 7.13 (d, J = 2.8 Hz, 1H), 6.57 (s, 1H), 3.55 – 3.45 (m, 2H), 2.98 – 2.88 (m, 2H), 1.98 (t, J = 7.7 Hz, 2H), 1.65 – 1.51 (m, 2H), 0.97 (s, 3H). LC-MS: t_R = 1.06 min, 95% by UV, HRMS m/z: $[M + H]^+$ calcd for $C_{16}H_{19}N_4OS$, 315.1279; found, 315.1288.

PhP098: *N*-((5-(4-methoxyphenyl)-1H-imidazol-2-yl)methyl)-3-(3-methyl-3H-diazirin-3-yl)propanamide (0.150 mmol, 47 mg, 100%) as an off-white solid. ^{1}H NMR (300 MHz, $CDCl_3$) δ 7.55 (d, J = 8.0 Hz, 2H), 7.20 – 7.06 (m, 2H), 6.92 (d, J = 7.8 Hz, 2H), 4.46 (d, J = 3.7 Hz, 2H), 3.82 (s, 3H), 2.14 – 2.00 (m, 2H), 1.76 (t, J = 7.2 Hz, 2H), 1.00 (s, 3H). LC-MS: t_R = 0.54 min, 100% by UV, HRMS m/z: $[M + H]^+$ calcd for $C_{16}H_{20}N_5O_2$, 314.1617; found, 314.1829.

PhP099: *tert*-butyl 4-((3-(3-methyl-3H-diazirin-3-yl)propanamido)methyl)benzylcarbamate (0,089 mmol, 25 mg, 82%) as an off-white solid. ^{1}H NMR (300 MHz, CD_3CN) δ 7.21 (s, 4H),

6.80 (s, 1H), 5.77 (s, 1H), 4.29 (dd, *J* = 6.1, 2.0 Hz, 2H), 4.17 (d, *J* = 6.3 Hz, 2H), 2.08 – 2.01 (m, 2H), 1.67 – 1.61 (m, 2H), 1.40 (s, 9H), 0.99 (s, 3H). LC-MS: t_R = 1.08 min, 97% by UV, HRMS m/z: $[M + H]^+$ calcd for $C_{18}H_{27}N_4O_3$, 347.2083; found, 347.2085

PhP100: *N*-(3-(2-(dimethylamino)acetamido)phenyl)-3-(3-methyl-3H-diazirin-3-yl)propanamide (0.045 mmol, 13.8 mg, 30%) as an off-white solid. ^1H NMR (300 MHz, $CDCl_3$) δ 9.38 (s, 1H), 7.84 (s, 1H), 7.68 (s, 1H), 7.33 (d, *J* = 9.4 Hz, 3H), 3.23 (s, 2H), 2.46 (d, *J* = 3.0 Hz, 6H), 2.18 (s, 2H), 1.83 (s, 2H), 1.05 (s, 3H). LC-MS: t_R = 0.44 min, 100% by UV, HRMS m/z: $[M + H]^+$ calcd for $C_{15}H_{22}N_5O_2$, 304.1773; found, 304.1777.

6. Supplementary Note 2. Intact MS protocols and results

6.1 Screening against BRD4-BD1 and $KRas^{G12D}$

Intact protein masses were recorded by LC-MS using an Agilent G6224 time-of-flight (ToF) Accurate Mass Series mass spectrometer, interfaced with an Agilent 1200 series liquid chromatography and sample handling system. The protein sample was injected using an Agilent 1200 series AutoSampler (Model No. G1367B) with a 10 µL injection volume and maintained at a temperature of 10 °C. Chromatography was carried out on an Agilent Bio-HPLC PLRP-S (1000Å, 5 µm × 50 mm × 1.0 mm, PL1312-1502) reverse phase HPLC column at 70 °C. Using an Agilent 1200 series binary pump system (Model No. G1312B) the sample was eluted at 0.5 mL/min using a gradient system from Solvent A (water, 0.2% (v/v) formic acid) to Solvent B (acetonitrile, 0.2% (v/v) formic acid) according to the following conditions:

Elution gradient (% B) used for intact protein LC-MS

Time (min)	% B
0	20
0.5	20
0.51	40
2.5	80
2.51	100
4	100
4.01	20
4.5	20

The eluent was injected directly into an Agilent ToF mass spectrometer (Model No. G6224A) using a dual ESI source and scanning between 600-3200 Da with a scan rate of 1.03 s in positive mode. The following MS parameters were used: capillary voltage limit – 4200; desolvation temperature – 340 °C; drying gas flow – 8.0 l/min. Data acquisition was carried out in 2 GHz Extended Dynamic range mode. Spectra were processed using Mass Hunter Qualitative Analysis™ B06.00 (Agilent) software with the Maximum Entropy method employed. The total ion chromatograms (TIC) were extracted (region containing protein) and the summed scans were deconvoluted (using a maximum entropy algorithm) over a m/z range with an expected mass range dependent on the protein.

6.2 Screening against STAT5B-NTD

For screening against STAT5B-NTD, we have used a UPLC-MS system that consisted of a Waters ACQUITY UPLC I-Class setup coupled with a Waters ACQUITY UPLC Peptide BEH C18 Column (130Å, 1.7 µm, 2.1 mm X 100 mm), connected to a Waters Xevo G2-XS QToF instrument equipped with a Waters Z-spray ESI source. During the analysis, the column temperature was maintained at a constant 60°C, and a sample volume of 3 µL was injected for each analysis. We utilized a gradient elution method with two eluents: eluent A, which consisted of 0.1% formic acid in water, and eluent B, composed of 0.1% formic acid in acetonitrile. The flow rate was set at 0.6 mL/min, and each measurement run lasted for 5.4 minutes. High-quality LC-MS grade solvents were sourced from Merck (Darmstadt, Germany). The gradient program is included below. Data acquisition was conducted in positive ion mode within the 100-2000 m/z (mass-to-charge ratio) range. The MS parameters were configured as follows: a source temperature of 150°C, a capillary voltage of 3.0 kV, and a desolvation temperature of 550°C. Nitrogen was employed as the atmospheric pressure ionization gas, supplied by a Genius 3020 Nitrogen generator from Peak Scientific. To control the analytical equipment and process the acquired data, we utilized the Waters Masslynx V4.2 SCN996 software package. Spectrum deconvolution was performed using Maximum Entropy modelling (MaxEnt) with the following parameters: a mass range of 7000-30000 Da, 1.00 Da/channel, a 0.75 Da width at half height with a uniform Gaussian damage model, and iterative refinement to convergence (approximately 40 iterations).

Gradient program for the MS screening against STAT5B-NTD

**Time (min)**	**%A** (0.1% formic acid in water)	**%B** (0.1% formic acid in acetonitrile)
0.0	95.0	5.0
5.0	0.60	70.0
1.80	0.0	100.0
2.20	0.0	100.0
2.70	95.0	5.0
3.00	30.0	70.0
3.00	70.0	30.0
3.60	30.0	70.0
3.90	70.0	30.0
4.20	30.0	70.0
4.50	70.0	30.0
4.80	5.0	95.0
5.40	95.0	5.0

6.3 Intact MS spectra of hit fragments vs. BRD4-BD1

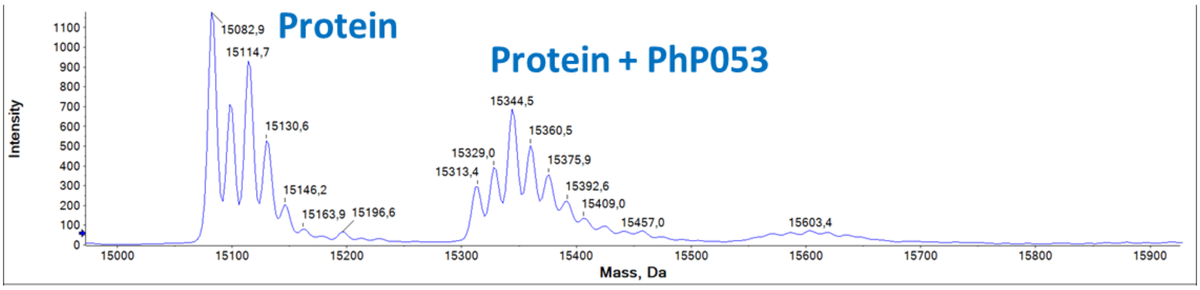

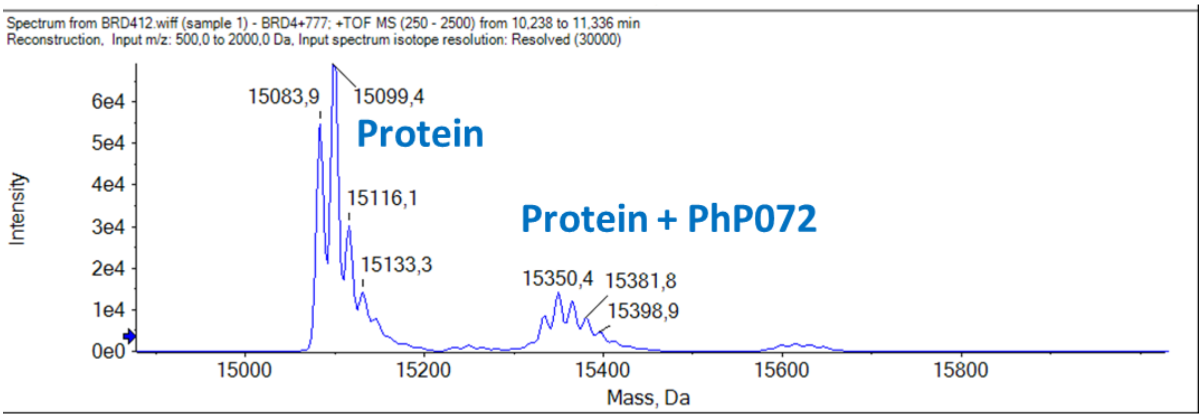

6.4 Intact MS spectra of hit fragments vs. KRas^{G12D}

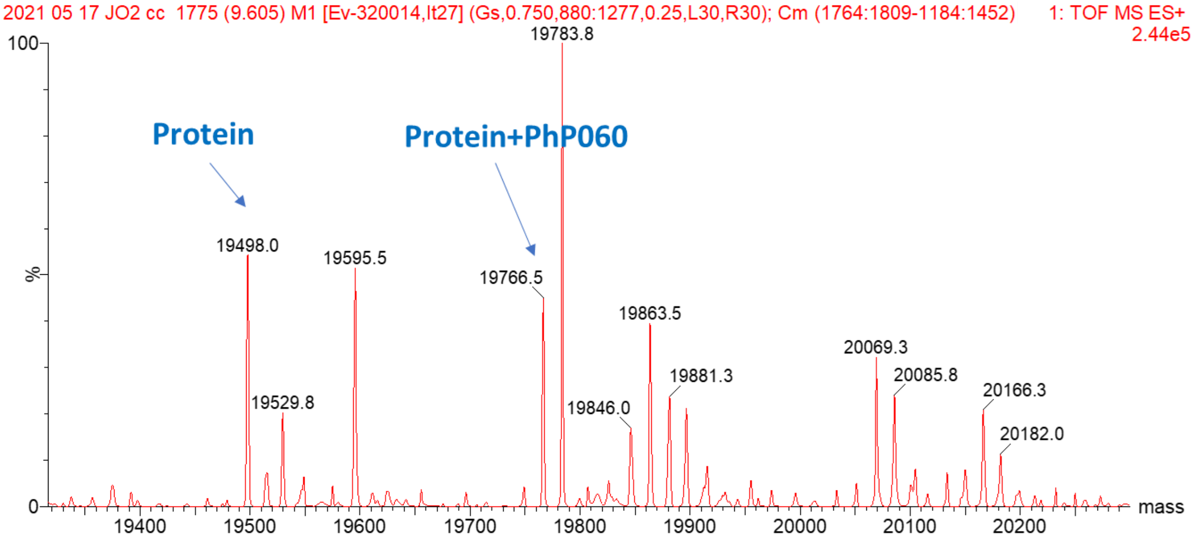

706 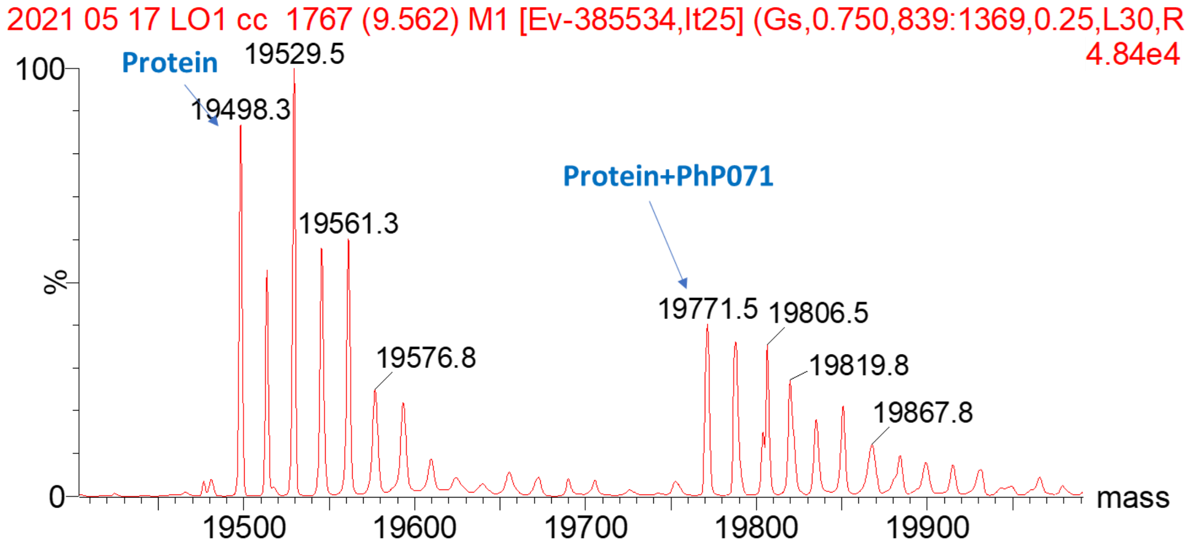

2021 05 17 LO1 cc 1767 (9.562) M1 [Ev-385534,It25] (Gs,0.750,839:1369,0.25,L30,R
4.84e4
100
Protein
19529.5
19498.3
Protein+PhP071
19561.3
%
19771.5
19806.5
19576.8
19819.8
19867.8
0
mass
19500
19600
19700
19800
19900

707 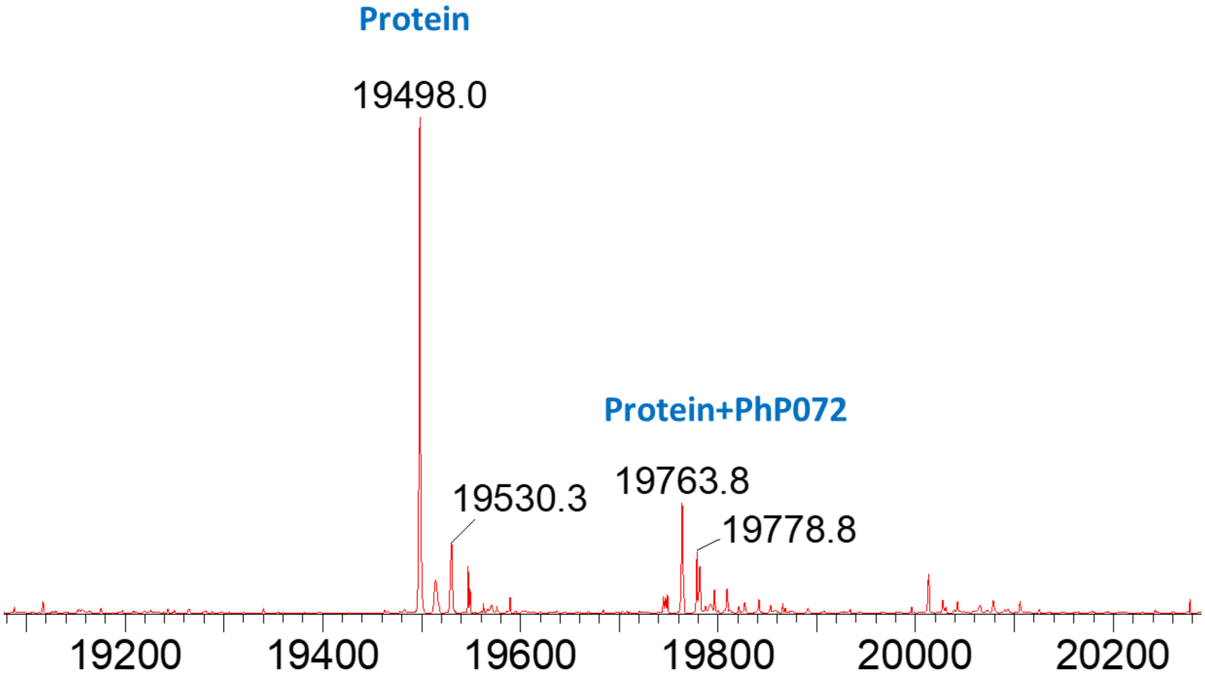

Protein
19498.0
Protein+PhP072
19763.8
19530.3
19778.8
19200
19400
19600
19800
20000
20200

6.5 Intact MS spectra of hit fragments vs. STAT5B-NTD

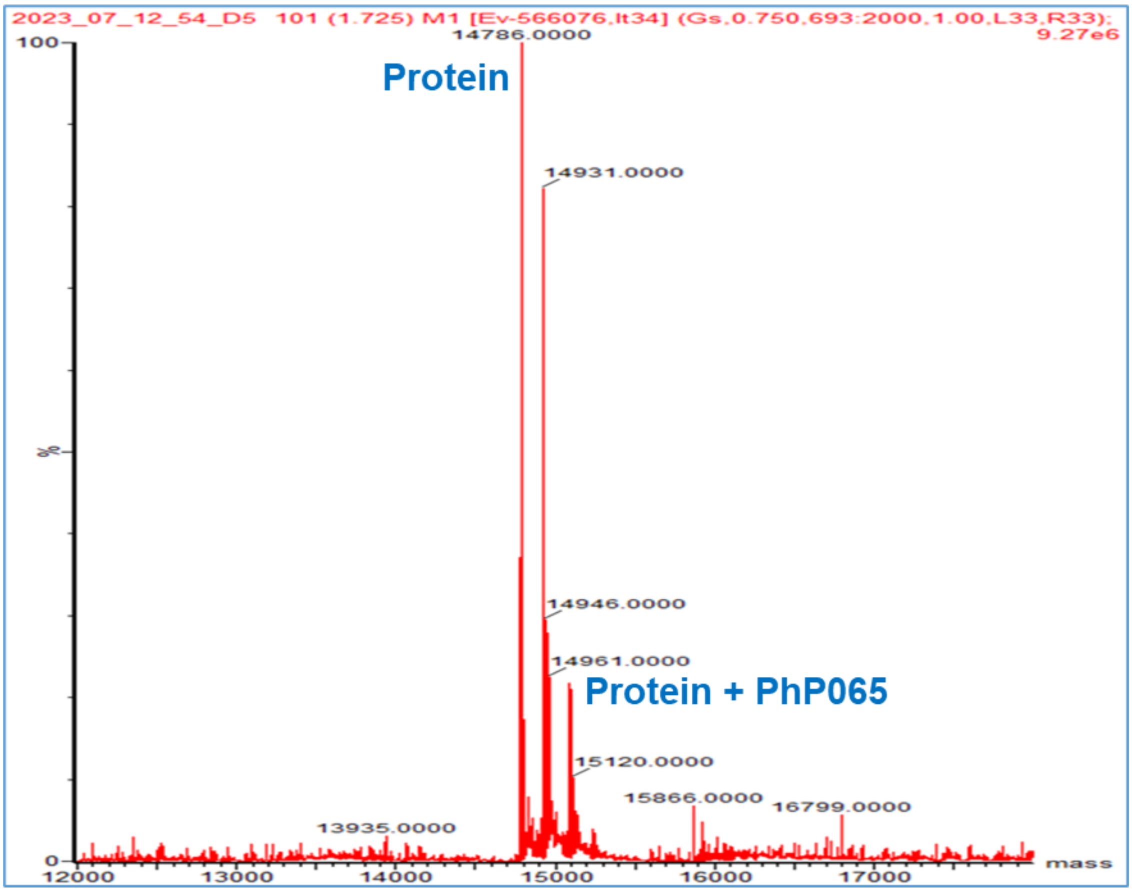

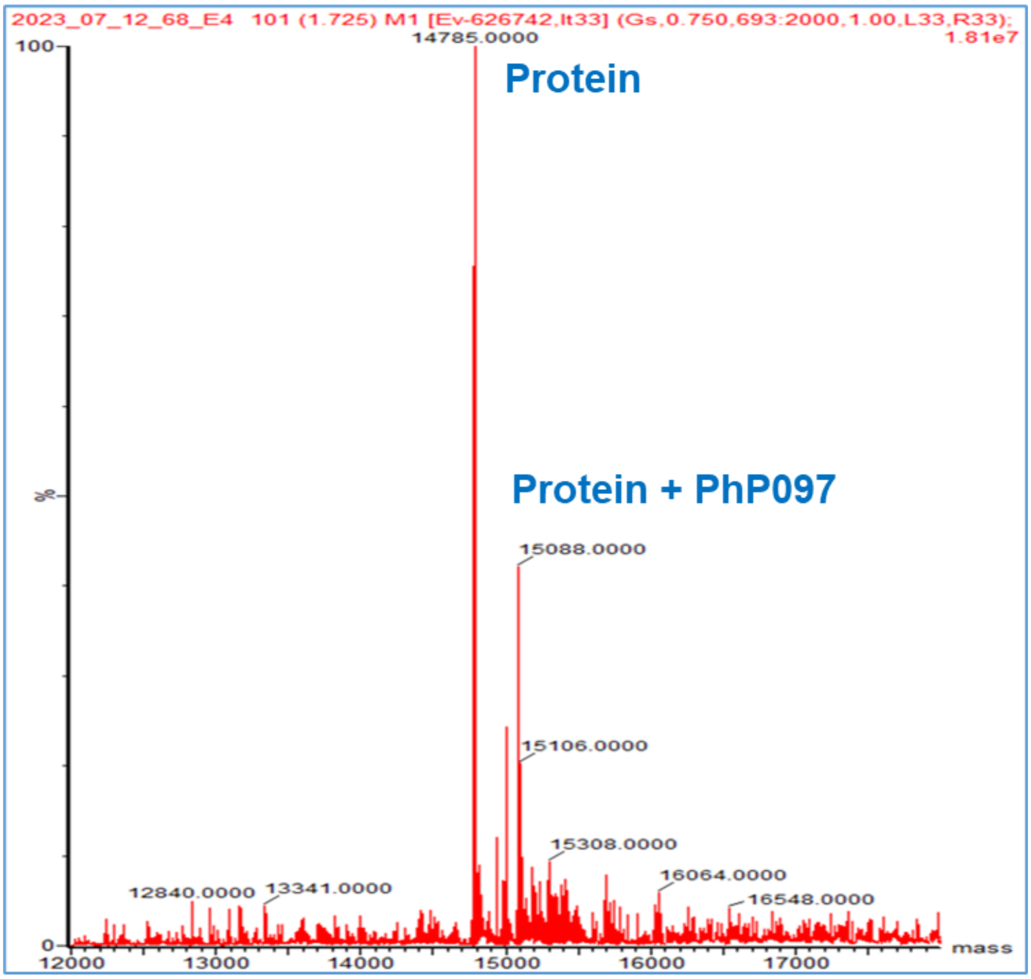

7. Supplementary Note 3. Binding site identification by LC-MS/MS peptide mapping

7.1 Sample preparation and data acquisition for BRD4-BD1

After the labelling was completed, 45 µL of the sample and 10 µL 0.2% (w/v) RapiGest SF (Waters, Milford, USA) solution buffered with 50 mM ammonium bicarbonate were mixed (pH=7.8) and 6.8 µL of 45 mM dithio-treitol (DTT) in 100 mM NH4HCO3 were added and kept at 37.5 °C for 30 min. After cooling the sample to room temperature, 8 µL of 100 mM iodoacetamide in 100 mM NH4HCO3 were added and placed in the dark at room temperature for 30 min. The reduced and alkylated protein was then digested by 5,5 µL (1 mg/mL) Trypsin/Lys C mix (the enzyme-to-protein ratio was 1:10) (Promega, Madison, USA). The sample was incubated at 37 °C for overnight. To degrade the surfactant, 6.8 µL of formic acid (500 mM) solution was added to the digested protein sample to obtain the final 40 mM concentration (pH ≈ 2) and was incubated at 37 °C for 45 min. For LC-MS analysis, the acid treated sample was centrifuged for 5 min at 13 000 rpm and the supernatant was pipetted into a microvial.

To get more precise information on the structure, samples were further analyzed by a Triple TOF 5600+ hybrid Quadrupole-TOF LC/MS/MS system (Sciex, MA, USA) equipped with a DuoSpray IonSource coupled with a Shimadzu Prominence LC20 UFLC (Shimadzu, Japan) system consisting of quaternary pump, an autosampler and a thermostated column compartment.

Data acquisition and processing were performed using Analyst TF software version 1.7.1 (AB Sciex Instruments, CA, USA). Chromatographic separation was achieved on the Discovery® BIO Wide Pore C-18-5 (250 mm × 2.1mm, 5 µm, 300 Å) HPLC column. Sample was eluted in gradient elution mode using solvent A (0.1% formic acid in water) and solvent B (0.1% formic acid in ACN). The initial condition was 5% B for 7 min, followed by a linear gradient to 90% B by 48 min, from 55 to 63 min 90% B was retained; and from 63 to 65 min back to initial condition with 5 % eluent B and retained for 10 min. Flow rate was set to 0.2 ml/min. The column temperature was 40 °C and the injection volume was 15 µl. Nitrogen was used as the nebulizer gas (GS1), heater gas (GS2), and curtain gas with the optimum values set at 35, 35 and 35 (arbitrary units), respectively. The source temperature was 350 °C and the spray voltage was set to 5000 V.

Advanced Information Dependent Acquisition (IDA) mode was used on the TripleTOF 5600+ system to obtain MS/MS spectra on the 8 most abundant parent ions present in the TOF survey scan. In IDA LC-MS/MS experiment the mass spectra and tandem mass spectra were recorded in “high-sensitivity” mode with a resolution of ~35,000 full-width half-maximum.

In first period (positive TOF MS mode) the data were acquired in the mass range of m/z=300 to 2500, with 0.1 s accumulation time. Declustering potential value was set to 60 V. The intensity threshold for precursor ion selection in TOF survey scan mode was 1000 cps. In MS2 experiment (Product Ion scan mode): the mass range was m/z=50 to 3000, with an accumulation time of 0.1 sec.

PeakView® V.2.2 software (version 2.2, Sciex) and Biologics Explorer software (version 3.0.3, Sciex) were used to assign and evaluate the peaks in the MSMS spectra.

7.2 Sample preparation and data acquisition for STAT5B-NTD

After the labelling was completed, 50 µL of the sample and 10 µL 0.2% (w/v) RapiGest SF (Waters, Milford, USA) solution buffered with 50 mM ammonium bicarbonate were mixed (pH=7.8) and 3.5 µL of 45 mM dithio-treitol (DTT) in 100 mM NH4HCO3 were added and kept at 37.5 °C for 30 min. After cooling the sample to room temperature, 4.5 µL of 100 mM iodoacetamide in 100 mM NH4HCO3 were added and placed in the dark at room temperature for 30 min. The reduced and alkylated protein was then digested by 7 µL (1 mg/mL) trypsin (the enzyme-to-protein ratio was 1:10) (Sigma, St Louis, MO, USA). The sample was incubated at 37 °C for overnight. To degrade the surfactant, 6 µL of formic acid (500 mM) solution was added to the digested protein sample to obtain the final 40 mM concentration (pH ≈ 2) and was incubated at 37 °C for 45 min. For LC-MS analysis, the acid treated sample was centrifuged for 5 min at 13 000 rpm and the supernatant was pipetted into a microvial.

To get more precise information on the structure, samples were further analyzed by a Triple TOF 5600+ hybrid Quadrupole-TOF LC/MS/MS system (Sciex, MA, USA) equipped with a DuoSpray IonSource coupled with a Shimadzu Prominence LC20 UFLC (Shimadzu, Japan) system consisting of quaternary pump, an autosampler and a thermostated column compartment.

Data acquisition and processing were performed using Analyst TF software version 1.7.1 (AB Sciex Instruments, CA, USA). Chromatographic separation was achieved on the Discovery® BIO Wide Pore C-18-5 (250 mm × 2.1mm, 5 µm, 300 Å) HPLC column. Sample was eluted in gradient elution mode using solvent A (0.1% formic acid in water) and solvent B (0.1% formic

acid in ACN). The initial condition was 5% B for 7 min, followed by a linear gradient to 90% B by 48 min, from 55 to 63 min 90% B was retained; and from 63 to 65 min back to initial condition with 5 % eluent B and retained for 10 min. Flow rate was set to 0.2 ml/min. The column temperature was 40 °C and the injection volume was 15 µl. Nitrogen was used as the nebulizer gas (GS1), heater gas (GS2), and curtain gas with the optimum values set at 35, 35 and 35 (arbitrary units), respectively. The source temperature was 350 °C and the spray voltage was set to 5000 V.

Advanced **Information Dependent Acquisition (IDA**) mode was used on the TripleTOF 5600+ system to obtain MS/MS spectra on the 8 most abundant parent ions present in the TOF survey scan. In **IDA** LC-MS/MS experiment the mass spectra and tandem mass spectra were recorded in “high-sensitivity” mode with a resolution of ~35,000 full-width half-maximum.

In first period (positive TOF MS mode) the data were acquired in the mass range of m/z=300 to 2500, with 0.1 s accumulation time. Declustering potential value was set to 60 V. The intensity threshold for precursor ion selection in TOF survey scan mode was 1000 cps. In MS2 experiment (Product Ion scan mode): the mass range was m/z=50 to 3000, with an accumulation time of 0.1 sec.

PeakView® V.2.2 software (version 2.2, Sciex) and Biologics Explorer software (version 3.0.3, Sciex) were used to assign and evaluate the peaks in the MSMS spectra.

7.3 MS spectra of digested BRD4-BD1 after labeling by hit fragments

MS/MS of the peptide INELPTEE, modified by **PhP053** at: ^{168}Glu

Peptide	Start	End	RT (Min)	Calculated Peptide Mass (Da)
INELPTEE	161	168	22.49	1173.5656

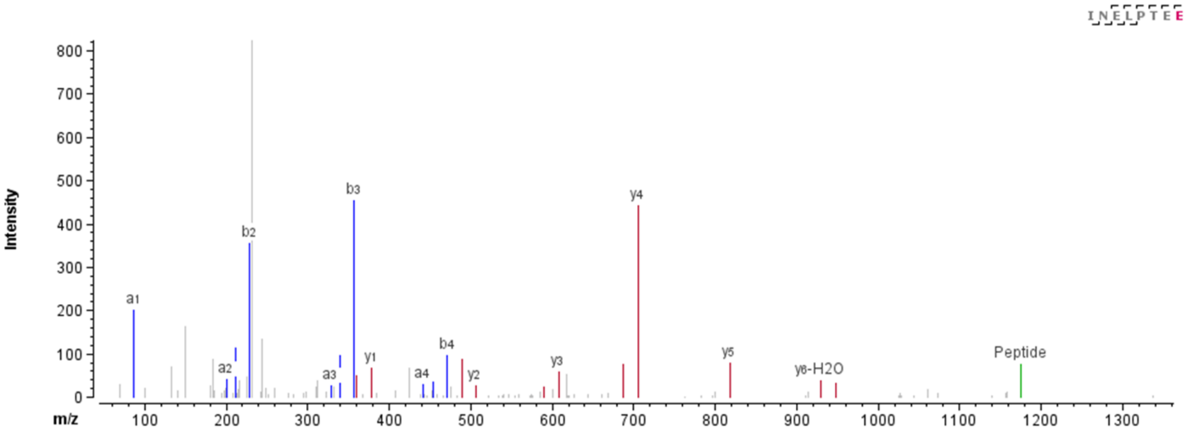

MS/MS of the peptide SMNPPPPETSNPNKPK, modified by **PhP072** at: ^{46}Pro

Peptide	Start	End	RT (Min)	Calculated Peptide Mass (Da)
SMNPPPPETSNPNKPK	42	57	10.92	1989.9487

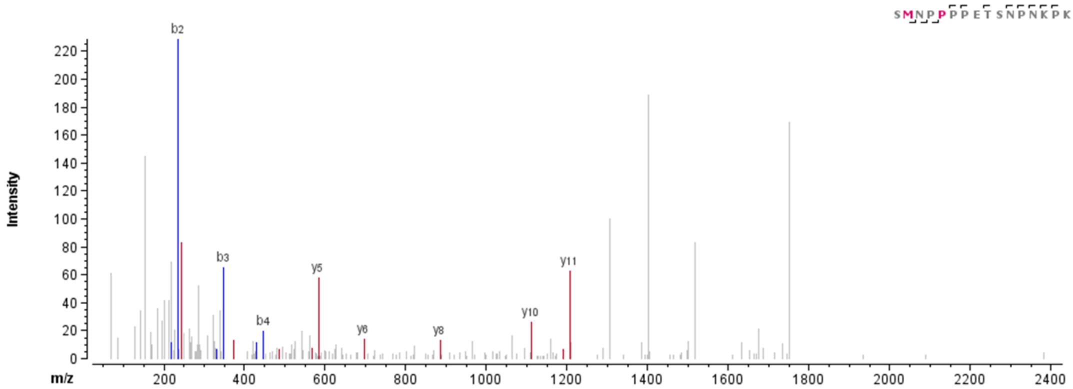

MS/MS of the peptide INELPTEE, modified by **PhP072** at: ^{166}Thr

Peptide	Start	End	RT (Min)	Calculated Peptide Mass (Da)
INELPTEE	161	168	22.55	1183.5632

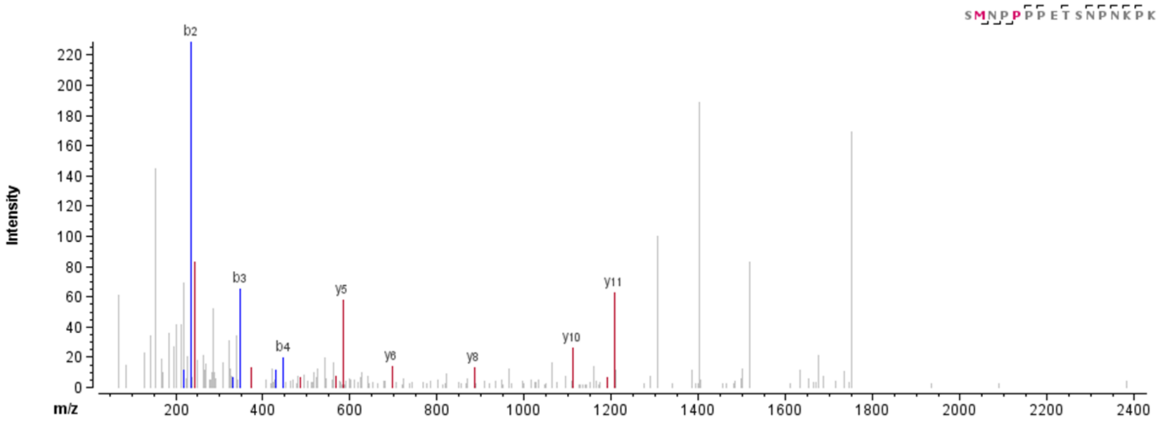

7.4 MS spectra of digested KRas^{G12D} after labeling by hit fragments

MS/MS of the peptide CDLPSR, modified by **PhP060** at: ^{118}Cys

Peptide	Start	End	RT (Min)	Calculated Peptide Mass (Da)	m/z	b/y Found	Mass Error (ppm)	Charge State	Mass (Da)	Intensity (Counts)
CDLPSR	118	123	12.2	786.3331	787.3413	4	0.4	1	786.3334	280646

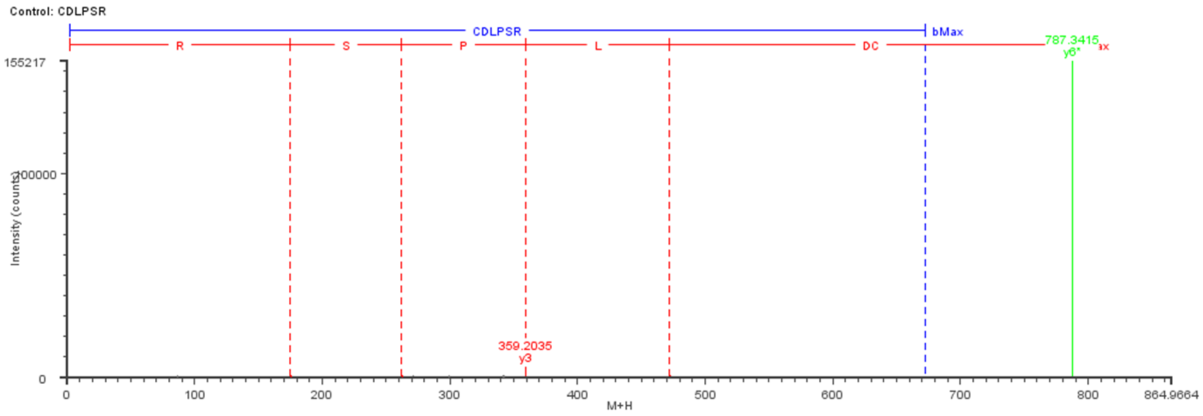

MS/MS of the peptide SALTIQLIQNHFVDEYDPTIEDSYRK, modified by **PhP060** at: ^{33}Asp

Peptide	Start	End	RT (Min)	Calculated Peptide Mass (Da)	m/z	b/y Found	Mass Error (ppm)	Charge State	Mass (Da)	Intensity (Counts)
SALTIQLIQNHFVDEYDPTIEDSYRK	17	42	36	3394.6357	849.6675	6	0.8	4	3394.638	4659

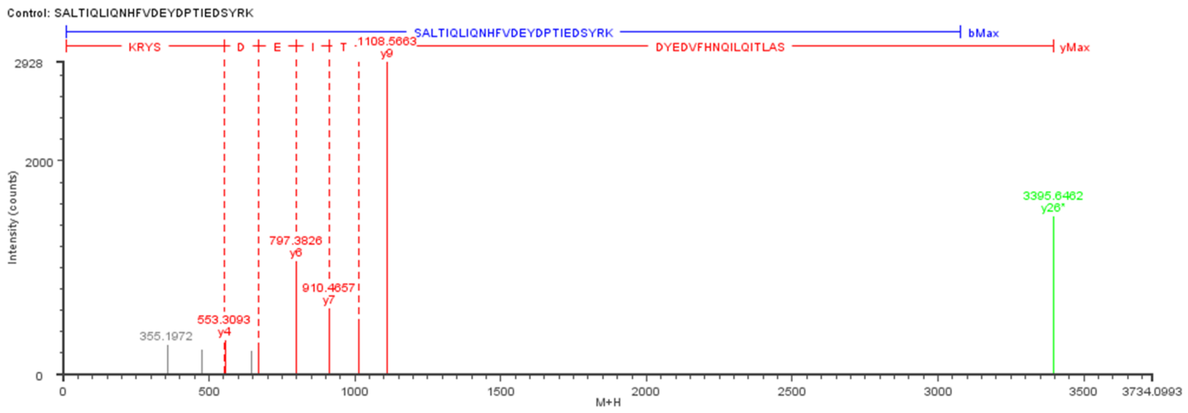

MS/MS of the peptide VKDSEDVPMVLVGNKCDLPSR, modified by **PhP071** at: ^{118}**Cys**

Peptide	Start	End	Control RT (Min)	Calculated Peptide Mass (Da)	Measured m/z	b/y Found	Mass Error (ppm)	Charge State	Measured Mass (Da)
VKDSEDVPMVLVGNKCDLPSR	103	123	27	2575.241	644.8201	31	2.9	4	2575.2485

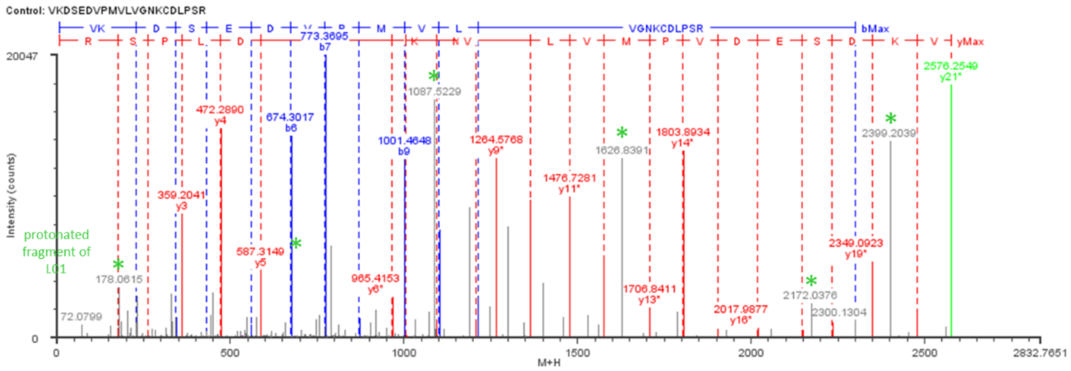

MS/MS of the peptide SFEDIHHYR, modified by **PhP072** and oxidized at: ^{95}His or ^{96}Tyr

Peptide	Start	End	RT (Min)	Calculated Peptide Mass (Da)	m/z	b/y Found	Mass Error (ppm)	Charge State	Mass (Da)	Intensity (Counts)
SFEDIHHYR	89	97	17.3	1468.6735	490.5669	5	2.3	3	1468.6769	72535

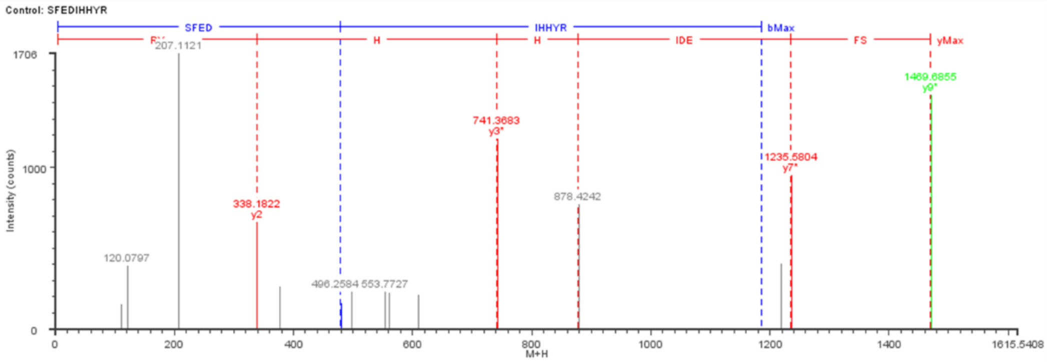

MS/MS of the peptide SALTIQLIQNHFVDEYDPTIEDSYRK, modified by **PhP072** and oxidized at: ^{33}Asp

Peptide	Start	End	RT (Min)	Calculated Peptide Mass (Da)	m/z	b/y Found	Mass Error (ppm)	Charge State	Mass (Da)	Intensity (Counts)
SALTIQLIQNHFVDEYDPTIEDSYRK	17	42	34	3360.6514	841.1728	8	2.4	4	3360.6594	10522

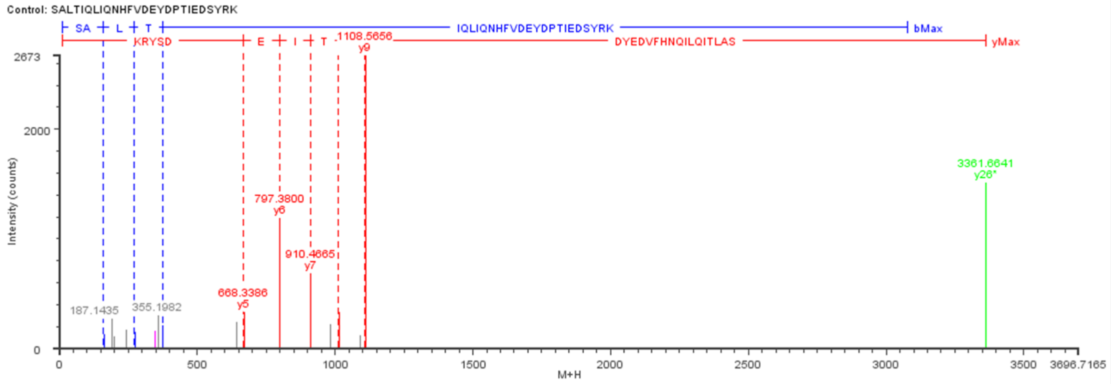

7.5 MS spectra of digested STAT5B-NTD after labeling by hit fragments

MS/MS of the peptide AEHQVGEDGFLLK, modified by **PhP065** at: ^{74}His

Peptide	Start	End	RT (Min)	Calculated Peptide Mass (Da)
AEHQVGEDGFLLK	72	84	28.73	1729.8498

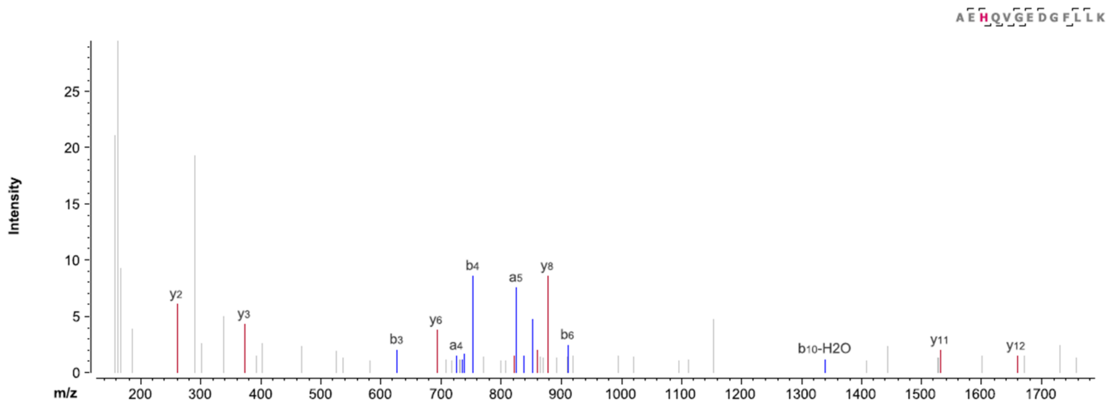

MS/MS of the peptide CPMELVR, modified by **PhP065** at: ^{101}Cys

Peptide	Start	End	RT (Min)	Calculated Peptide Mass (Da)
CPMELVR	101	107	30.79	1134.5388

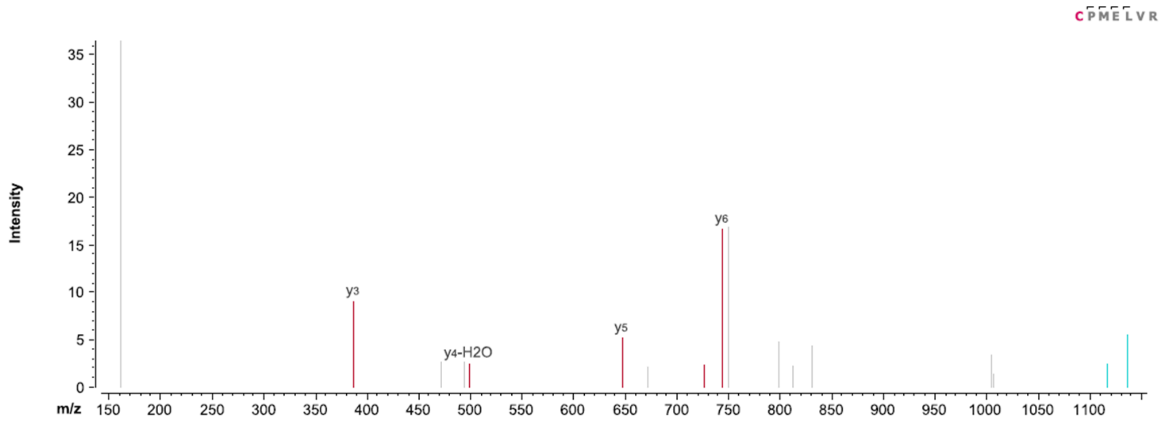

MS/MS of the peptide ATQLLEGLVQELQK, modified by **PhP097** at: ^{57}Ala

Peptide	Start	End	RT (Min)	Calculated Peptide Mass (Da)
ATQLLEGLVQELQK	57	70	32.55	1723.9769

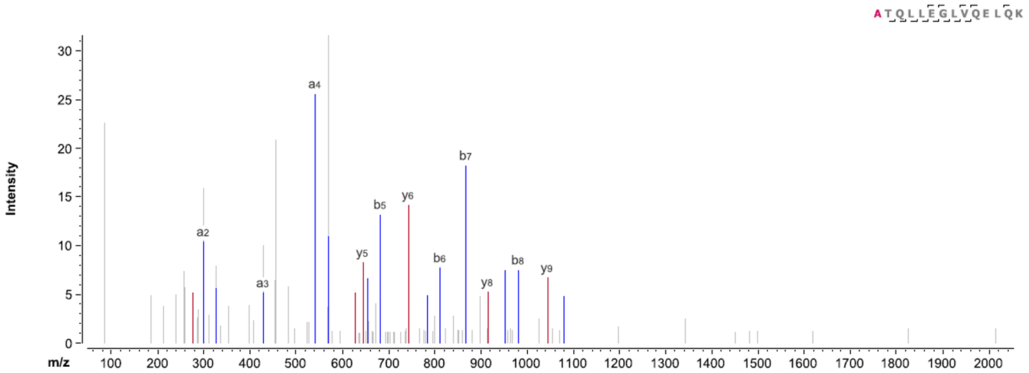

MS/MS of the peptide AEHQVGEDGFLLK, modified by **PhP097** at: ^{72}Ala

Peptide	Start	End	RT (Min)	Calculated Peptide Mass (Da)
AEHQVGEDGFLLK	72	84	24.89	1546.7252

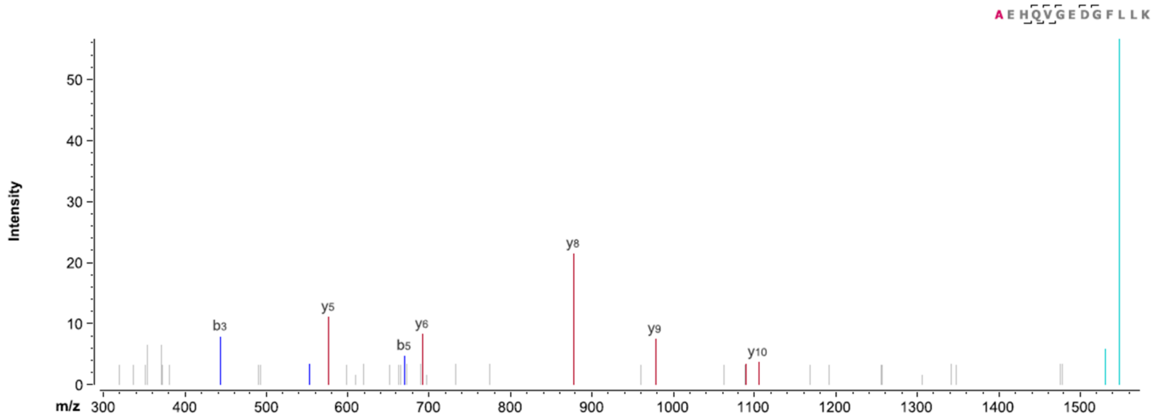

MS/MS of the peptide CPMELVR, modified by **PhP097** at: ^{101}Cys

Peptide	Start	End	RT (Min)	Calculated Peptide Mass (Da)
**CPMELVR**	101	107	27.1	1049.4573

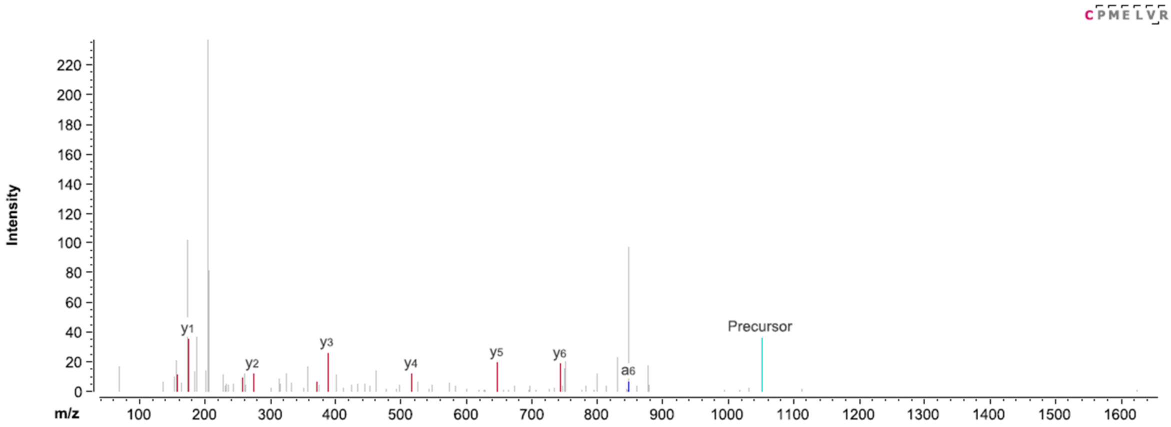

8. Supplementary Note 4. HSQC NMR spectra of fragment hits against KRas^{G12D}

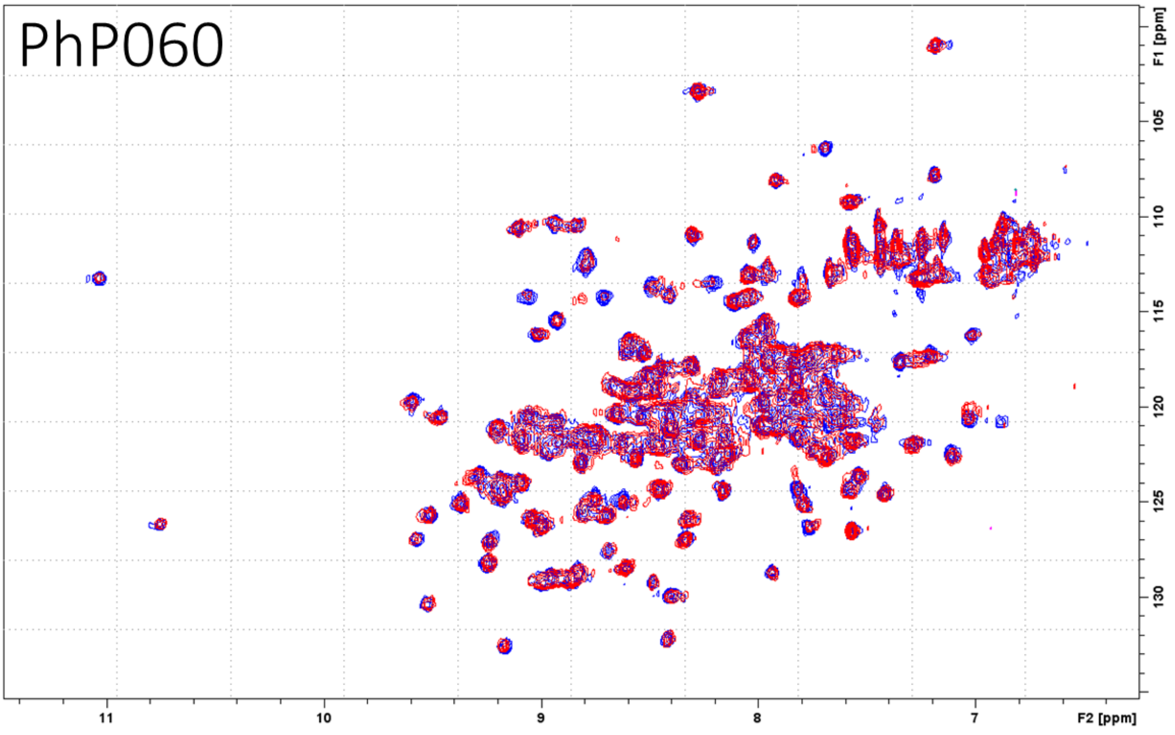

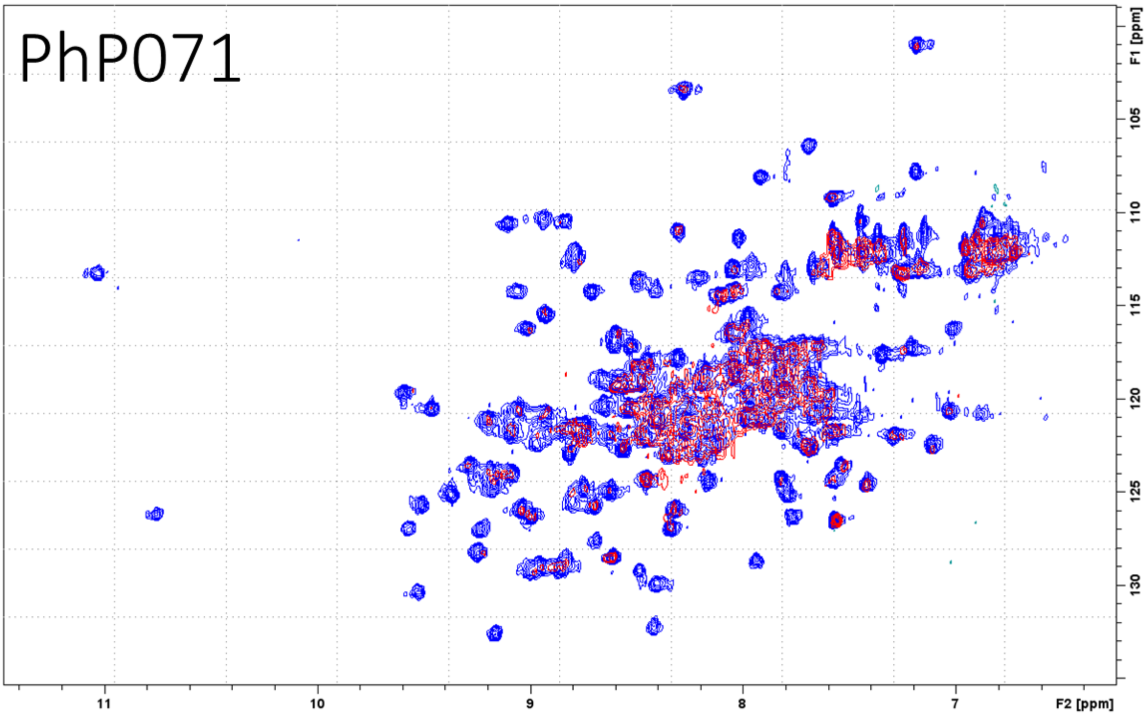

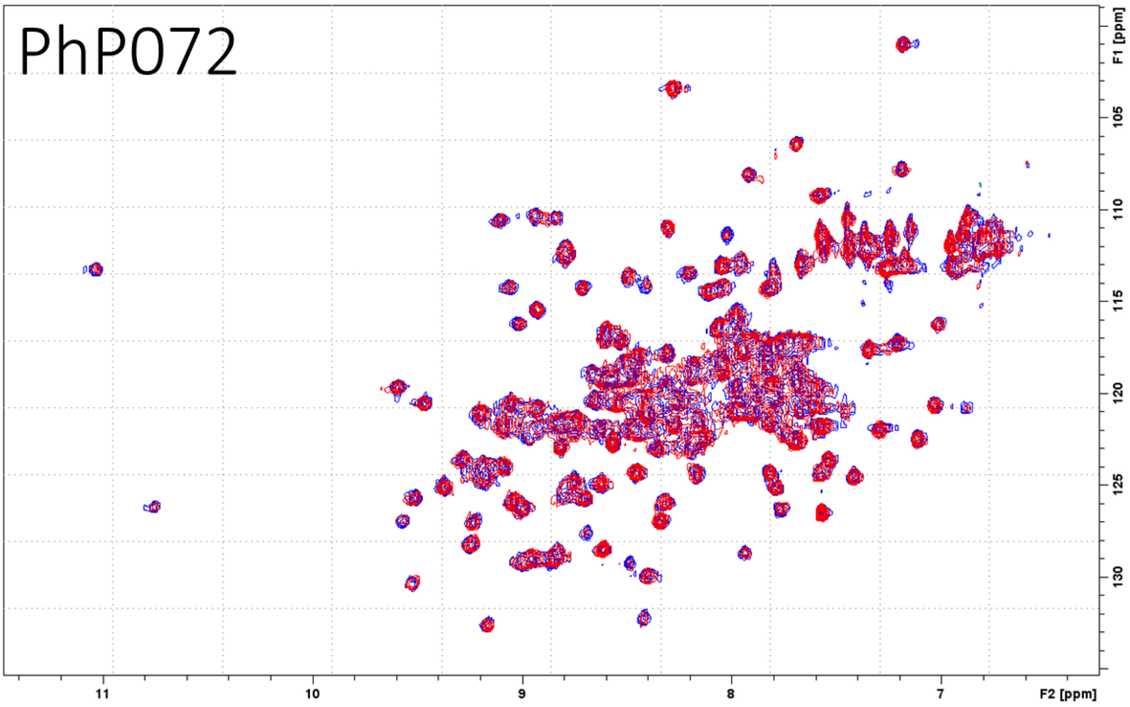

9. Supplementary References

1. Grant, E. K. *et al.* A Photoaffinity-Based Fragment-Screening Platform for Efficient Identification of Protein Ligands. *Angewandte Chemie International Edition* **59**, 21096–21105 (2020).

2. Trowbridge, A. D. *et al.* Small molecule photocatalysis enables drug target identification via energy transfer. *Proceedings of the National Academy of Sciences* **119**, e2208077119 (2022).
