# Supplementary material for: Mapping protein binding sites by photoreactive fragment pharmacophores
Source: Commun Chem. 2024 Jul 31;7:168. doi: 10.1038/s42004-024-01252-w (PMC11292009; doi:10.1038/s42004-024-01252-w)
Supplement: Supplementary file 2 — Description of Additional Supplementary Files [file 42004_2024_1252_MOESM2_ESM.pdf]

Description of Additional Supplementary Files

File name: Supplementary Data 1

Description: List of compounds in the PhP library with experimental hit status, pharmacophore fingerprints, SMILES strings and 2D structures. MS labelling percentages (%) dataset on protein targets. Refinement statistics of the X-ray structure 8Q34.

File name: Supplementary Data 2

Description: 1H NMR spectra of the PhP fragments.

File name: Source Data 1

Description: Source Data for Figures
