# Supplementary material for: Mapping protein binding sites by photoreactive fragment pharmacophores
Source: Commun Chem. 2024 Jul 31;7:168. doi: 10.1038/s42004-024-01252-w (PMC11292009; doi:10.1038/s42004-024-01252-w)
Supplement: Supplementary file 4 — Supplementary data 2 [file 42004_2024_1252_MOESM4_ESM.docx]

**Supplementary data** 2.

^1^H NMR spectra of the PhP fragments

Péter Ábrányi-Balogh^1,2,3§^, Dávid Bajusz^1,2§^, Zoltán Orgován^1,2§^, Aaron B. Keeley^1^, László Petri^1,2^, Nikolett Péczka^1,2,3^, Tibor Viktor Szalai^1,2,4^, Gyula Pálfy^5^, Márton Gadanecz^2,5^, Emma K. Grant^6^, Tímea Imre^2,7^, Tamás Takács^2,8,9^, Ivan Ranđelović^10,11^, Marcell Baranyi^11,12^, András Marton^13,14^, Gitta Schlosser^15^, Qirat F. Ashraf^16^, Elvin de Araujo^17^, Tamás Karancsi^13,14^, László Buday,^2,8^ József Tóvári^10^, András Perczel^2,5^, Jacob Bush^6^, György M. Keserű*^1,2,3^

*See full list of affiliations in the article.*

§These authors contributed equally to this work.

*Corresponding author: György M. Keserű, email: keseru.gyorgy@ttk.hu

grease

H2O

DMSO-d6

DMSO-d6

H2O

DMSO-d6

H2O

DMSO-d6
